# Leaf versus whole-canopy remote sensing methodologies for crop monitoring under conservation agriculture: a case of study with maize in Zimbabwe

**DOI:** 10.1038/s41598-020-73110-3

**Published:** 2020-09-29

**Authors:** Adrian Gracia-Romero, Shawn C. Kefauver, Omar Vergara-Díaz, Esnath Hamadziripi, Mainassara A. Zaman-Allah, Christian Thierfelder, Boddupalli M. Prassana, Jill E. Cairns, José L. Araus

**Affiliations:** 1 Integrative Crop Ecophysiology Group, Plant Physiology Section, Faculty of Biology, University of Barcelona, Barcelona, Spain; 2 AGROTECNIO (Center for Research in Agrotechnology), Av. Rovira Roure 191, 25198 Lleida, Spain; 3 International Maize and Wheat Improvement Center, CIMMYT Southern Africa Regional Office, Harare, Zimbabwe

**Keywords:** Agroecology, Imaging and sensing, Plant physiology, Plant stress responses

## Abstract

Enhancing nitrogen fertilization efficiency for improving yield is a major challenge for smallholder farming systems. Rapid and cost-effective methodologies with the capability to assess the effects of fertilization are required to facilitate smallholder farm management. This study compares maize leaf and canopy-based approaches for assessing N fertilization performance under different tillage, residue coverage and top-dressing conditions in Zimbabwe. Among the measurements made on individual leaves, chlorophyll readings were the best indicators for both N content in leaves (R < 0.700) and grain yield (GY) (R < 0.800). Canopy indices reported even higher correlation coefficients when assessing GY, especially those based on the measurements of the vegetation density as the green area indices (R < 0.850). Canopy measurements from both ground and aerial platforms performed very similar, but indices assessed from the UAV performed best in capturing the most relevant information from the whole plot and correlations with GY and leaf N content were slightly higher. Leaf-based measurements demonstrated utility in monitoring N leaf content, though canopy measurements outperformed the leaf readings in assessing GY parameters, while providing the additional value derived from the affordability and easiness of using a pheno-pole system or the high-throughput capacities of the UAVs.

## Introduction

Currently, Sub-Saharan Africa (SSA) has one of the lowest cereal self-sufficiency ratios of the world while also having one of the greatest projected increases in population^1^. By 2050, the population in SSA is expected to grow 2.5-fold, requiring a tripling of the actual cereal production in order to meet demand^2^. The staple crop in SSA is maize, but its production is being limited by a decline in soil fertility. Particularly, Zimbabwe has been considered a hotspot for both nutrient and water limitation in agricultural production^3^. Traditional practices of monoculture and soil tillage have led to a decline in soil fertility^4^, causing the use of N fertilizers to become essential. Yet, this situation cannot be considered sustainable given the economic and environmental impact associated with high fertilization rates^5^.

In this context, reducing N fertilizer rates without implicating major losses in grain yield (GY) is a way of preserving natural resources and the environment without compromising food security while facing the projected changes in temperature and precipitation patterns. To that end, apart from breeding for improved plant varieties, changes in agricultural management must be considered, too. Conservation agriculture (CA), characterized by minimum soil disturbance, permanent soil cover and diversified crop rotations, has being promoted as a pragmatic solution for increasing yields while conserving natural resources. Conventional tillage (CT) practices (i.e. conventional plough-based practices) improves the aeration of the soil but may result in detrimental effects to the environment and hence lead to yield decreases in long term^6^. Soil compaction is managed by deep tillage, but this mechanical disturbance has also been shown to lead to long-term declines in organic matter, an increase in water loss by runoff, and soil erosion^4^. Reducing or avoidance of soil erosion helps to retain soil moisture and reduces the use of fossil fuels, thus lowering costs and chances of total crop loss due to drought^7^. On the other hand, the application of plant residues usually leads to an increase in crop yields due to its benefits to water retention and improved soil fertility, but its success relies on the amount and quality of the residues and the initial fertility status of the soil^7^. The application of residue resources, such as crop stover, in combination with mineral fertilizers is being increasingly implemented to address declines in soil fertility^8^. However, an important drawback of the promotion of CA practices is the competing uses of crop residues (e.g. livestock feed, as fuel or for construction) that act against their use in CA mulch applications^9–12^. Also, poorer farmers often sell their residues to livestock keepers^13^. A better understanding of the minimum crop residue mulching thresholds that are required in order to provide CA benefits to farmers would allow farmers the flexibility to remove biomass for other purposes. Moreover, improvements in crop residue management practices may produce relevant changes towards enhancing the potential sequestration of organic carbon by farmlands, as an option for mitigation of greenhouse emissions.

Still, an appropriate N fertilizer use regimen under CA is crucial to promote microbiological activity^14^. For this reason, N management programs must be critically evaluated, including application rate and timing as well as the type of the N fertilizer used. On-field, fast and non-destructive indicators of crop nutritional status, such as leaf chlorophyll meters have been used for N fertilization monitoring, as chlorophyll concentration is strongly related to the N status of the plant^15^. The most often used leaf-clip device is the SPAD-502 from Minolta-Konica that assesses Chl concentration from leaf transmittance^16^. A newer alternative is the three-in-one instrument Dualex from Force-A, that, besides chlorophylls (Chl a + b), also measures leaf epidermal flavonoids (Flav) and anthocyanins (Anth)^17^. However, the main limitation of the leaf-clip-type instruments for large-scale studies is that these techniques are time consuming. One potential solution is the use of remote sensing methodologies for data collection at the canopy level, which have become valuable tools for precision agriculture and high-throughput plant phenotyping^18^. Besides multispectral sensors and imagers, further opportunities are found in the use of conventional digital Red–Green–Blue (RGB) cameras as low-cost tools for crop monitoring. Images are used to produce RGB indexes based on the color properties of the canopy, which have become very useful in forecasting yield and assessing crop variability^19^. The assessment of the photosynthetic area of the canopy as well as the stay-green capacity during the crop cycle are important factors for determining grain yield^20^. The successful implementation of aerial platforms with the assembly of imaging sensors has been extensive for assessing crop performance under different growing conditions, permitting the screening of a large number of plots precisely and efficiently. In terms of monitoring/phenotyping platforms, the use of unmanned aerial vehicles (UAVs, a.k.a. drones) represents an increasingly common option, particularly considering the popularization of drones^21^. Nevertheless, the adoption of drone technology can be limited by both lack of economic resources and restrictive laws associated with the use of aerial vehicles (manned and unmanned). In such cases, an innovative option for canopy assessments of tall crops like maize or fruit trees is the attachment of a camera to a pole that may reach several meters above the crop. This alternative might require more time for data acquisition compared to UAV measurements, but less technical skills are required by the staff for image acquisition and further processing, in terms of the image alignment in orthomosaics, the posterior extraction of the individual plots or other image processing that may be required when using UAVs. Thus, for example in the case of CA and maize, increases in the performance of vegetation indices for assessing crop yield has been reported when the images were subject to pre-processing, such as applying a soil cover mask for segregating the crop biomass from the soil residue cover^22^.

Besides remote sensing evaluations, laboratory (i.e. analytical) traits, may be also deployed for crop phenotyping and monitoring^23^. The stable carbon (δ^13^C) and nitrogen (δ^15^N) isotope compositions, when analyzed in plant matter, inform on the water regimen and nitrogen metabolism conditions, respectively, of the plant^24,25^. Even in the case of a C4 species like maize, δ^13^C may still differentiate between water growing conditions^26^. In fact, both isotopic signatures have been used before in maize for assessing the effect of tillage practices^22^ and nitrogen fertilization^25^ on the water and nitrogen growing conditions of the crop, even when treatments differences for both traits were only found when comparing different N fertilization levels within a common tillage system^25^.

The main focus of this study is to compare the performance of a set of single-leaf and canopy-based remote sensing indices for assessing the influence of the top-dressing levels and the combination of tillage and residue levels on maize yield and N leaf content. Two different specialized portable leaf pigment-meters, as well as leaf scans for measuring the color of the leaves were used to assess the leaf N content. Concerning the canopy scale assessments, RGB images were taken at the ground level from a height of 4 m a.g.l. (above the ground level) using a pheno-pole and from the aerial level at a height of 30 m a.g.l. using a UAV. As a complementary selection strategy, carbon and nitrogen isotope signatures were analyzed in the leaves, as a potential tool for evaluating water and nitrogen status or differences in N assimilation.

## Results

### Crop yield response to tillage, the residues and top-dressing application and the associated interactions

**Figure 1:**
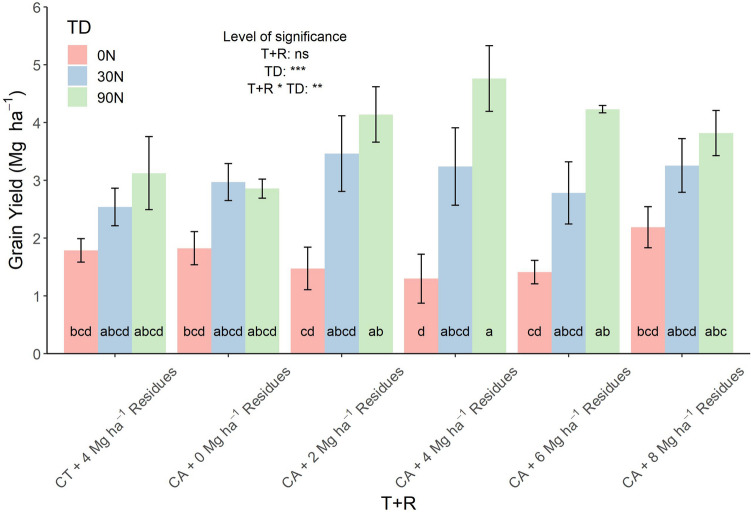
Average maize grain yield across the growing conditions. CA corresponds to plots grown under conservation agriculture management and CT to conventional tillage plots. T + R corresponds to the levels of the combination effect of tillage and residue application, TD to the Top-dressing levels and T + R * TD to the interaction of both factors. The error bars show the standard error of the five replicates. Different letters (a, b, c, d) indicate significant differences between the residue and top-dressing treatments according to Fisher’s LSD test. Significance levels of the ANOVAs: *p* < 0.05; **p < 0.01; ***p < 0.001; *ns* no significant.

Tillage, residue application and the top-dressing levels effects on the grain yield (GY) were evaluated (Fig. 1). The factor residue application did not report significant effects on GY (*p* value = 0.657), but no-tillage plots responded with increasing yield to the residue application up to 6 Mg ha^−1^, but GY decreased when the residue application was increased to 8 Mg ha^−1^. Within each residue treatment, the increase of topdressing applications resulted in a significant yield improvement (*p* value = 0.000***), except for in the application of 2 Mg ha^−1^ of residues, where the plots without N fertilization were still outperformed in terms of yield by those plots with 4 or 6 kg ha^−1^ AN in the top-dressing. The interaction of both, treatment and sub-treatments, which had a significant effect on GY (*p* value = 0.007**) was grouped in four homogeneous groups. Of these, the treatment combining the application of 6 Mg ha^−1^ of residues together with the highest top-dressing level was clearly identified alone as the highest-yielding condition (4.76 Mg ha^−1^ of GY). The lowest yield was achieved under the application of 6 Mg ha^−1^ of residues but without N fertilizer (1.29 Mg ha^−1^ of GY). When the levels of N were null or low at the top-dressing treats, the CT produced higher yields than the no-tillage (with the exception of the 30N conditions). However, the application of fertilizers with elevated N fertilization levels increased yields in the conditions with 4, 6 and 8 Mg ha^−1^ of residues in comparison to the CT conditions. The higher the residue application was, the higher the positive effect of the top-dressing N treatment on grain yield.

### Effects of growing conditions on leaf total nitrogen content and carbon and nitrogen stable isotope compositions

**Figure 2:**
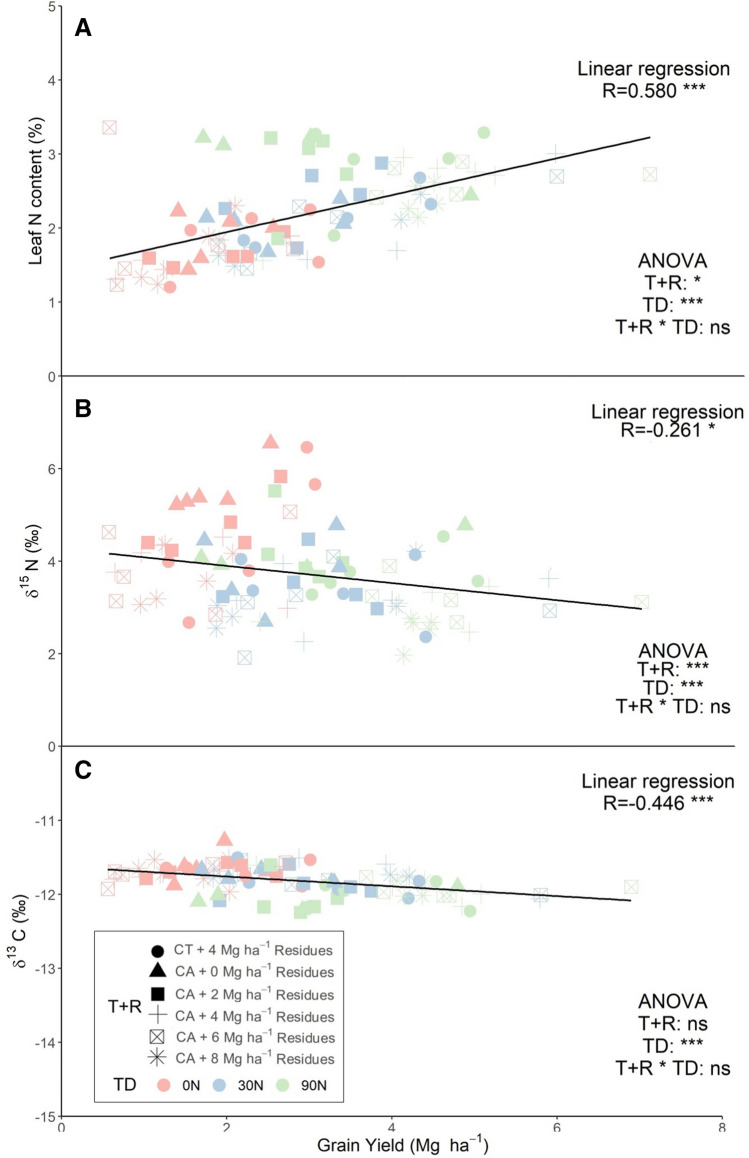
Relationship between the leaf N content (**A**), the N (**B**) and the C isotope (**C**) composition with grain yield. Correlations were studied across the 90 plots from all the growing conditions. CA corresponds to plots grown under conservation agriculture management and CT to conventional tillage plots. T + R corresponds to the levels of the combination effect of tillage and residue application, TD to the Top-dressing levels and T + R * TD to the interaction of both factors. Significance levels of the correlations and ANOVAs: ns, *p* > 0.05; *p < 0.05; **p < 0.01; ***p < 0.001.

Tillage and residue treatment as well as the top-dressing fertilization had a significant effect on the total N leaf content (Fig. 2A) and its isotope signature composition (Fig. 2B). The main differences in the leaf N content were caused by the top-dressing (*p* < 0.000***), with the highest values at 90N (2.83%) in comparison to the other two sub-treatments (1.17% for 0N and 1.98% for 30N) (Supplemental Table 1). Comparing the soil preparation conditions, when the residue application was the same, conventional tillage plots showed higher N content in their leaves. The leaf N content decreased significantly (*p* = 0.044*) across the residue application, with the highest values at 0 Mg ha^−1^ and the lowest at 8 Mg ha^−1^. A strong positive correlation between the N leaf content and the GY was found. The CT treatment presented higher values of δ^15^N than the no-tillage treatments at the same residue conditions. In contrast to the N content, the correlation of the δ^15^N with GY was weaker and negative. On the other hand, the δ^13^C exhibited significant differences across the application of top-dressing reporting more negative values with the increase of AN fertilizer, but no significant differences were attributed to the residue levels (Fig. 2C). More negative δ^13^C values corresponded to higher GY, reporting higher correlations.

### Implications of growing conditions on the leaf pigments readings and the RGB index derived from the scans

The conditions derived from both residue and the top-dressing applications significantly influenced the leaf pigment readings (Table 1). The variance analysis showed there were significant differences in the effects of growing conditions on all the leaf pigments. The chlorophyll values, from both devices, were clearly benefited by the top-dressing applications (*p* < 0.001***), reporting the highest values in the plots grown under 90N conditions (SPAD: 48.31 and Dualex: 38.27) (Supplemental Table 1). Regarding the differences between the two devices, the SPAD readings were slightly higher than the measurements with the Dualex, whereas the value difference between both sensors moved by the same percentage through the experimental conditions (Supplemental Figure 1).

**Table 1:** Effect of the combination of the tillage and residue application (T + R), the top-dressing (TD) and the combination of both factors (T + R * TD) on the leaf pigment readings.

	T + R	TD	T R * TD
**Dualex**			
SPAD	ns	1.017e−13***	ns
Chl	ns	1.426e−11***	ns
Flav	ns	4.384e−06***	ns
Anth	ns	1.241e−11***	ns
NBI	ns	2.612e−09***	ns

Significance levels of the ANOVAs: no significant (ns), *p* > 0.05; *p < 0.05; **p < 0.01; ***p < 0.001.

By contrast, the measurements of the Flav and Anth content responded significantly to top-dressing, showing a reduction with increasing N top-dressing (negative correlation). Finally, the Chl/Flav ratio represented as the NBI index increased with the nitrogen top-dressing (Supplemental Figure 1).

Leaf scan images were processed to measure different indices describing color parameters (Supplemental Table 2). Indices related to the greenness of the image (Hue, a*, u*, GA, GGA and NGRDI) were very close to their saturation limits (Table 2), but an increase in the green parameters pairing with the increase of the residue application could still be noticed in Hue, a* and v* indices. Conversely, most of the calculated indices reported significant differences across the top-dressing levels, except for the indices derived from the combination of the reflectance of the R, G and B bands, NGRDI and TGI. Darker shades of green could be observed at the scans of leaves grown under 90N conditions (Supplemental table 2) through the Hue (90.09° ± 0.43) or the a* values (− 20.67 ± 0.43), in comparison with the shades of green reported under the 0 N conditions (Hue: 86.23° ± 054 and a*: − 25.54 ± 0.39). The only index that responded significantly for both treatments and their interactive effect was Saturation. Saturation values increased with the residue application (*p* = 0.016*) but decreased with the top-dressing application (*p* < 0.000***). The indices GA and GGA were completely saturated showing values at their highest ranges beyond 0.95.

**Table 2:** Effect of the combined effect of the tillage conditions with the residue applications levels (T + RL) and the top-dressing (TD) on the RGB indices derived from the leaf scans, and the plot images taken from the ground and the aerial level.

	RGB scans			RGB ground			RGB aerial		
	T + RL	TD	T + RL * TD	T + RL	TD	T + RL * TD	T + RL	TD	T + RL * TD
Hue	ns	2.249e−07***	ns	0.02878*	5.304e−15***	ns	ns	1.878e−15***	ns
Intensity	ns	6.742e−11***	ns	ns	ns	ns	6.958e−05***	2.685e−16***	ns
Saturation	ns	3.308e−11***	ns	0.01413*	4.244e−12***	ns	0.033*	2e−16***	ns
GA	0.033*	0.0002957***	ns	ns	2e−16***	ns	ns	2e−16***	ns
GGA	ns	8.792e−08***	ns	ns	2e−16***	ns	ns	2.2e−16***	0.008**
CSI	ns	9.696e−08***	ns	0.044*	2.85e−11***	ns	ns	2e−16***	0.019*
Lightness	ns	9.798e−12***	ns	ns	ns	ns	1.337e−06***	2.2e−16***	ns
a*	ns	3.99e−11***	ns	0.039*	1.652e−15***	ns	ns	6.981e−09***	ns
b*	ns	3.504e−12***	ns	0.005**	4.472e−09***	ns	9.912e−05***	2.2e−16***	ns
u*	ns	2.268e−09***	ns	0.041*	5.324e−15***	ns	ns	3.996e−14***	ns
v*	ns	3.915e−12***	ns	0.004**	2.634e−05***	ns	5.568e−06***	2.2e−16***	ns
NGRDI	ns	ns	ns	ns	2e−16***	ns	ns	1.658e−14***	0.010*
TGI	ns	ns	ns	0.001**	3.825e−08***	0.006**	0.0001***	1.56e−06***	ns

These indices are defined in the “Methods” section. Significance levels of the correlations and ANOVAs: no significant (ns), *p* > 0.05; *p < 0.05; **p < 0.01; ***p < 0.001.

### Effects of growing conditions on the whole-canopy RGB indices measured from the ground and from the air

With regard to the ground RGB evaluation (Supplemental Table 3), all the indices coincided in informing that the treatment of conventional tillage with the application of 4 Mg ha^−1^ of residues exhibited the greenest canopies (Hue: 77.66° ± 3.22 and a*: − 13.49 ± 0.94). Concerning the indices derived from the aerial images, however, the greenest plots were reported under the no-tillage conditions with 6 Mg ha^−1^ of residues (Hue: 64.94 ± 2.57° and a*: − 10.83 ± 0.62). For both levels (ground and aerial) of measurement, the values of the indices that estimated the greenness of the canopy under CA increased with the application of residues till 6 Mg ha^−1^ and started to decrease with 8 Mg ha^−1^. Contrarily to the RGB indices derived from the scans on single leaves, the greenness measurements at canopy level decreased significantly as the top-dressing levels diminished, presenting the lowest values at the 0N conditions (from the ground level = Hue: 60.35° ± 1.62 and a*: − 8.16 ± 0.62; from the aerial level = Hue: 56.97° ± 1.77 and a*: − 8.53 ± 0.39). Besides, the shades of yellow, expressed by the b* component, increased significantly with the residue application but were significantly reduced with the increment of the top-dressing level, for both levels (ground and aerial) of measurement.

### Performance of the leaf and canopy-based measurements monitoring N and predicting GY

**Figure 3:**
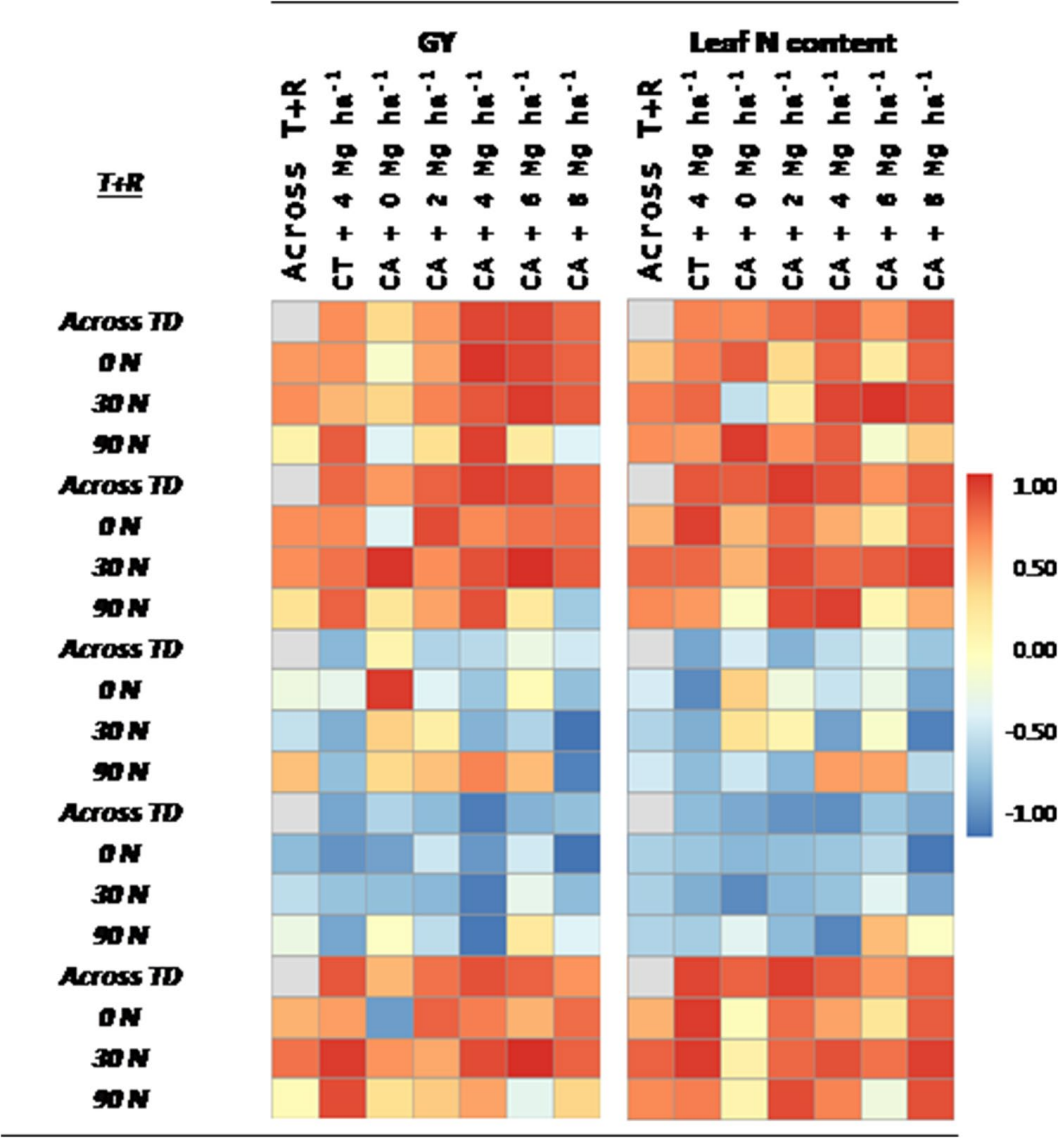
Heat map of Pearson correlation coefficients (R values) between the leaf-clip sensor readings with the grain yield (GY) and the N leaf content inside each growing condition, across treatments (Across T) and across the combination of reside levels and treatments (Across R + T). CA corresponds to plots grown under conservation agriculture management and CT to conventional tillage plots. Correlations colors are scaled according to the key above.

To assess the accuracy of the leaf and canopy-based measurements for the assessment of the leaf N content and the GY prediction, the determination coefficients across the growing conditions were performed (Fig. 3). Chlorophyll readings, regardless the leaf clip used, showed very similar behavior as the leaf N content, reporting high and positive correlations between them. The correlations between chlorophyll measurements and GY were slightly lower, but still strong and significant. Nevertheless, the chlorophyll measurements derived from the Dualex were slightly better correlated to the N content and GY than the SPAD readings. Flav and Anth readings correlated negatively to N content, but only Anth correlated negatively to GY. The NBI reading highly correlated positively with both N content and GY.

**Figure 4:**
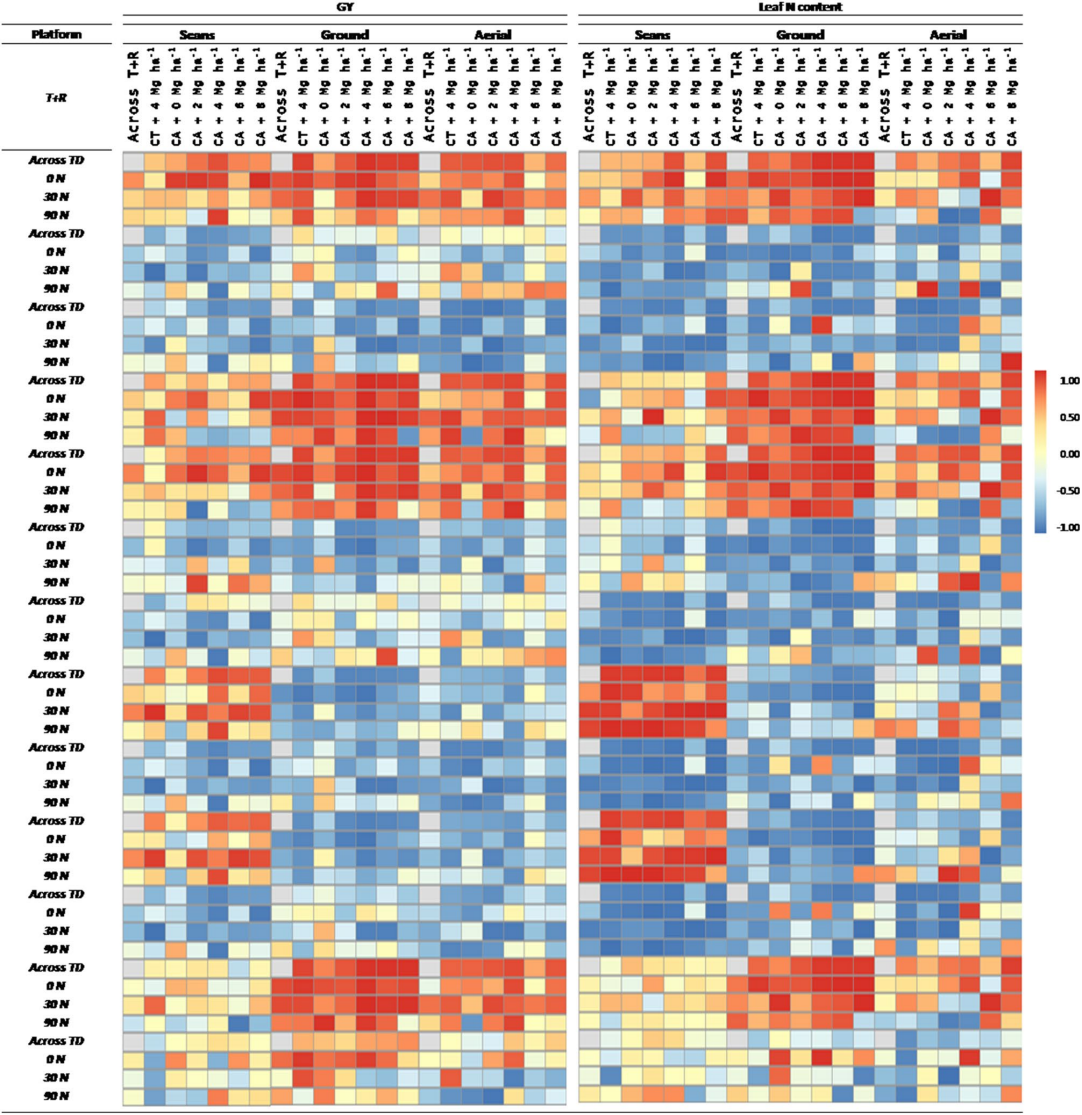
Heat map of Pearson correlation coefficients (R values) between the RGB indices derived from leaf scans, and from the ground and aerial canopy images against the GY and the leaf N content inside each growing condition, across treatments (Across T) and across the combination of reside levels and treatments (Across R + T). CA corresponds to plots grown under conservation agriculture management and CT to conventional tillage plots. Correlations colors are scaled according to the key above.

Correlation coefficients for the relationships of the leaf N content and GY with the RGB indices derived from the leaf scans and the ground and aerial canopy images are presented in Fig. 4. According to the RGB leaf scans, greenness measures corresponding to Hue, a* and u* indices correlated positively to N content. The measures more related to the yellow color of the leaf, as the b* and the v*, and the Intensity, Saturation and Lightness reported negative correlations against GY. Regardless of the platform (from the ground or from the UAV), GA and GGA were the best correlated with the leaf N content, followed by Hue and NGRDI. Besides, CSI, a* and u* also correlated well, but negatively, against leaf N content. Except for the CSI, the prediction of the N content was slightly higher when measured from the ground. With reference to predicting GY, the performance of the indices was stronger than in estimating leaf N content and for most of the indices, excluding the a* and the NGRDI indices, the aerial assessments outperformed the ground measurements. Among all, the best correlated indices measured at ground level were the GA and the NGRDI. On the other hand, the best GY predictors measured form the aerial level were all the indices derived from the HSI color model (the Hue, the GA, the GGA and the CSI). For both (ground and aerial) platforms, the index that performed the worst in terms of assessing GY was the TGI. The canopy greenness-related indices derived from the HSI, RGB CIELab* and CIELuv* color systems presented a very similar capacity for assessing GY differences across the residue and top-dressing treatments. Moreover, almost all the correlation coefficients calculated were very high and consistent for both ground and aerial platform levels but being generally slightly higher at aerial level. The highest correlations were achieved at the no-tillage conditions with a residue application between 4 and 6 Mg ha^−1^. Besides, the lowest correlations were achieved at the no-tillage plots without any residue applications and under 90N top-dressing conditions.

### Leaf, canopy and aerial measurement calibrations

**Figure 5:**
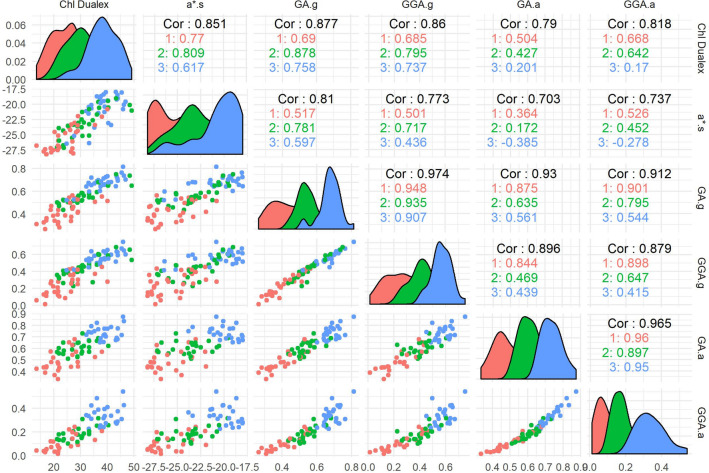
Diagnostic panel of each variable by itself and their relationship to each other categorized according the top-dressing treatments: 0N in red, 30N in green and 90N in blue. Bottom-left charts represent the scatter plot correlations and the upper-right represent the correlation coefficients. The Cor value corresponds the correlation across all treatments, the value 1 to the correlation inside the 0N plots, the value 2 to the correlation inside the 30N plots and the value 3 to the correlation inside the 90N plots. The diagonal shows a smoothed-out histogram of the values of the measures.

In order to validate the relationship between the parameters measured at different scales (leaf vs canopy) and placements (ground vs aerial) a pairwise comparison was performed (Fig. 5). The parameters selected were those measurements that best correlated to N leaf content and GY. The chlorophyll content measured with the Dualex was highly correlated to the greenness of the leaf derived from the a* index measured from the scans. When the GA and GGA canopy measurements were used, the correlations against Chl were still strong, regardless the observation height level (ground vs aerial). However, the relationship against the aerial measurements of GA and GGA were much weaker. The greenness indices of the leaf derived from the leaf scans paralleled the corresponding indices measured at canopy level; thus, strong correlations were found, particularly for the measurements at ground level, while for the aerial measurements the correlations inside each top-dressing treatment were low. Finally, the comparison between the canopy GA and GGA indices from the ground and aerial images resulted in very high correlation coefficients. Along with the scatter plot charts and correlation coefficients, density plots to assess the measurements distribution are also provided in the same panel.

## Discussion

### Influence of tillage, crop residues and top-dressing with non maize yield

Top-dressing with N fertilizer induced the most notable effect on the parameters assessed: GY, leaf N content, signature of stable C and N isotopes, leaf pigment content and the different vegetation indices measures at single leaf and canopy levels. Nitrogen is a major nutrient for crop production and our results showed a positive yield response to N application (Fig. 1). Albeit the effect of the residue level alone did not improve yield, the combination of top dressing with N resulted in a significant yield increase. Among the plots fertilized with the higher amounts of N, increasing the residue level had a positive effect on yield, reaching the top at 6 Mg ha^−1^ but decreasing with 8 Mg ha^−1^. Permanent residue soil cover helps to ensure better rainfall infiltration while reducing evaporative water losses^27^, therefore improving yields in low rainfall areas^28^. Moreover, the use of cereal stover increased short-term immobilization of N, having a potential positive effect on crop nutrient response^29^. However, an excess of residues can also be detrimental to crop emergence given the physical obstacles for seedlings or may provide a favorable habitat for plant pathogens^30^. Besides, the application of residues may also decrease, at least temporary, the availability of N for the plant, since it is used by microorganisms that decompose the residues into organic matter^31^ and thus limit GY. Fonte et al.^32^ presented similar results from the combination of fertilizer and residue effects on yield and also reported that the addition of N had the most consistent effect of increasing yield.

### Differences in leaf N content and N and C isotopic signatures

The top-dressing fertilizer rate increment resulted in an increase in the leaf N content, where the maximum leaf N content was obtained under the no-tillage conditions without residue application and the 90N fertilizer treatment (Fig. 2A). Once the leaves reach a threshold in N concentration, the plant aims to increase the biomass rather promoting the increase of the N concentration of the leaves, while the N concentration in the leaves further increase when the plant achieves its maximum growth^33^. The decrease reported in the N content of the leaves across the residue levels might be related to an increase of the microbiological activity at the top layers of the soil^31^. The nitrogen isotope composition has been used to study the dynamics of N in soil–plant systems^34^. Depending on the N source used as a N fertilizer the δ^15^N will vary, reporting values closer to zero when the origin of the N-fertilizer is synthetic^35^. As the top-dressing rate of N fertilization increased, the δ^15^N reported lower values, proving that δ^15^N can be used to characterize the level of N fertilization^36^. The decrease of the δ^15^N due the increase of the residue levels might be explained by the discrimination of the microorganism with the remaining soil N being impoverished in ^15^N^37^. The carbon isotope composition (δ^13^C) is an indicator of the water status of the plant; in the case of C4 species usually decreasing in response to water stress^26,38–40^. Even if at a much lesser extent than in C3 species, δ^13^C in the plant matter of C4 plants also depends on the intercellular to the atmospheric CO_2_ concentration of the leaf, which is affected by differences in water regime or in intrinsic photosynthetic capacity^26^. The lack of differences in δ^13^C discarded any improvement effect on the water status of the plants due to the residue level coverage. However, our results showed how a higher N concentration in leaves caused a decrease in δ^13^C. These results agree with Vergara-Díaz et al.^25^ This effect may be attributed to a boost in the photosynthetic capacity due to the increase of N concentration or alternatively to a greater associated transpiration area, causing some degree of water stress and a decrease in stomatal conductance. Both factors may lower the ratio of intercellular to atmospheric CO_2_, which, in the case of a C4 plant like maize, may cause a small decrease in δ^13^C^41^.

### Evaluation of leaf-based and whole-canopy measurements for monitoring leaf N content and predicting GY

Chlorophyll measurements exhibited the same trend as the leaf N content. Changes in leaf N content resulted in changes in the photosynthetic proteins, that represents a large portion of the total leaf N^42^. The close positive relationships between leaf chlorophyll values and N content demonstrated the potential to estimate in-season leaf N content of leaf tissues based on the SPAD or Dualex readings. As leaf chlorophyll content is very sensitive to variations in N supply, this parameter can be used for a quick detection of N deficiency^43,44^. Conversely, the response of Flav and Anth to leaf N content was negative (Fig. 3). Similar findings were presented in Zhang el al.^45^ where Flav and Anth were found to be particularly sensitive and consistent indicators of N fertilization conditions.

Grain yield comparisons to the leaf pigment readings also resulted in significant correlations. This agreed with the results presented in Cairns et al.^46^ where SPAD readings were significantly correlated with GY during grain filling. However, the potential of the relative leaf chlorophyll readings for predicting GY in maize could vary depending on the phenological stage when measurements are taken. Buchaillot et al.^47^ studied the variations in SPAD measures in assessing GY differences over two phenological stages before grain filling and reported higher correlations during the vegetative stage rather than during flowering. Monneveux et al.^48^ reported no significant correlations between SPAD and GY during neither middle nor late grain filling. Thus, it is very important to consider the timing of the measurement of leaf pigment contents for performing reliable GY predictions.

Because the color of the maize leaves is mainly determined by their content in chlorophylls and carotenoids^49^, digital color analysis might be also considered as a potential method for evaluating foliar nutrition. The leaf scans showed how the more N fertilizer was added, the greener the leaves were and the correlations of RGB indices (Hue, a* and u*) against N and chlorophyll were very high. The color tendency across the residue levels was lighter green tones (yellowish) as the amount of residue is increased. This is consistent with the above results, as the darker is the leaf’s green, higher is the amount of chlorophylls and the nitrogen content^50,51^. This can be clearly seen through the indices derived from the RGB scans. The CIE a* and u*components establish the color position between the red/magenta and the green, with negative values indicating green^52^, where inside this green range, more negative values indicate lighter green while less negative values indicate darker green. On the opposite way, the b* and v* positive values represent the yellow color spectrum^52^ and thus, the correlation of these indices against N content and GY is negative. Concerning the HSI parameters, lower Hue degrees correspond to more yellowish colors, and higher degrees correspond to darker green tones. The other two HSI parameters, Intensity and Saturation, inform about the brightness of the color^53,54^ and a decrease of their values matched with darker leaves (i.e. with higher chlorophyll contents and higher N content). Concerning the GA, GGA or CSI indices, while they are frequently used as good predictors of GY in field canopy measurements, which was confirmed in our study, they correlated poorly against GY when these indices were assessed at the single leaf level through the image scans. Similarly, the NGRDI and the TGI indices, as estimations of image greenness and indices formulated for canopy images, have been successfully applied for assessing GY at the canopy level^55,56^ but failed here at the leaf level due to saturated values.

**Figure 6:**
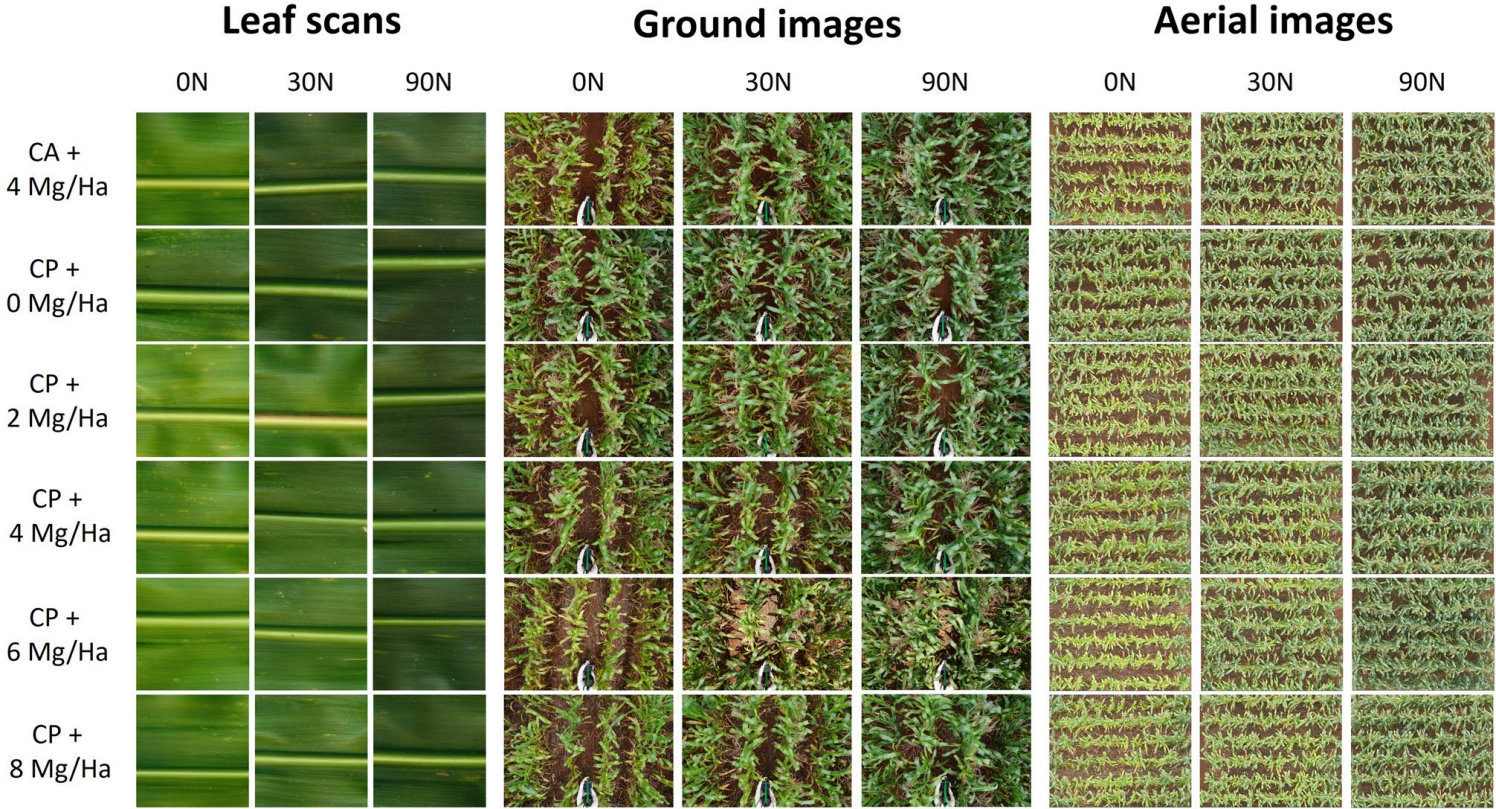
RGB leaf scans and canopy images taken from the ground and aerial level.

Canopy color related indices acquired from both the ground level and aerially performed worse for examining leaf N content than the single leaf-based indices, but, in terms of predicting GY, the canopy measurements performed better than those same indices at the leaf level. Conversely, at the canopy level, leaf color differences are less relevant and thus, the estimation of N content or chlorophylls resulted a bit more problematic (Fig. 6). However, considering that RGB canopy derived indices are known as effective measurements of green biomass, the strong correlations reported with the leaf N content might be more related to the N fertilization effects on growth rather than the leaf N content itself. Gracia-Romero et al.^57^ came to the same conclusion while studying the performance of RGB and multispectral indices assessing leaf phosphorous content in a maize trial. Nevertheless, one of the main benefits of the canopy images is to enable assessing the heterogeneity of the plot as a whole. Thus, canopy measurements have the potential to minimize the influences of the sampling location of the leaf better than the leaf-clip sensors, where the averaged values of 5–10 leaves and a sampling area of solely 6 mm^2^ (for both Dualex and SPAD) is assumed to be a representative measure of the plot. Attempts to improve the representativity of the single-leaf measurements, imply measuring always the same kind of leaves (flag leaves, top leaves…), while paying attention that leaves are fully intact, clean and free of signs of disease or damage^58^. However, the variability within the canopy is assumed to be captured with few measurements of individual leaves.

The indices that performed better assessing differences in GY were the ones related to vegetation cover, as the GA and the GGA. Both indices quantify the portion of green pixels, being GGA more restrictive by excluding the yellowish green fraction of vegetation, and therefore are considered reliable estimators of vegetation cover^59^. Another biomass-assessment index is the NGRDI, which is formulated similarly to the well-known Normalized Difference Vegetation Index (NDVI), but instead of using information from the near-infrared reflection bands, it incorporates information from the green band and thus it can be calculated with images from conventional RGB cameras^56^. The strength in assessing GY in the indices capturing green tonalities values (like the Hue or the a*) has a different explanation. Although these indices are not strict vegetation-density indicators as the GA or the GGA, the differences in crop cover between plots were the main source of variability rather than the canopy color itself. The measurements derived from those indices are indicators of the greenness of the image derived from the combination of effects of the chlorophyll concentration, the canopy green leaf area and the canopy architecture^60^. Otherwise, the Crop Senescence Index (CSI), as it is formulated from the combination of GA and GGA indices^61^, provides truthful information about the variation of the canopy color derived from the development of leaf senescence caused by growing conditions. The CSI reported a wide change across the growing conditions and highly correlated to GY. Earlier senescence due to low N fertilization conditions resulted in elevated CSI values, providing efficacy in plant stress detection. In fact, RGB canopy indices have been proven to perform far better in predicting GY than the NDVI or other multispectral indices in other maize and wheat studies^22,57,62^.

### Comparison of measurements scales (leaf vs canopy based, and ground vs aerial) in assessing maize performance

The canopy remote sensing methodologies, when applied from aerial platforms, can be considered as robust approaches for rapidly assessing a large number of plots, particularly for large scale field-based studies. In this study, the ground level images taken with a 4 m pole only permitted coverage of a portion of the plot and therefore did not account for the possible heterogeneity of the plot; the time spent on fieldwork to cover the 90 plots was approximately one hour. On the other hand, the aerial images permitted assessing the totality of the plot area and the flight duration for covering the whole trial was less than 10 min (including the pre-flight procedures). However, aerial images require preparation before image processing, including building the image mosaics and segmenting the plots, compared to the ground images, which can be processed directly. In terms of accuracy, ground evaluation images had a much higher spatial resolution (5456 × 3632 pixels), while aerial images had much lower resolution (478 × 379 pixels for a flight at 30 m a.g.l.). Despite these differences, the RGB indices from both platforms were highly correlated and their precision in assessing leaf N content was very similar and for GY prediction even higher in the case of the aerial measurements. Thus, UAV imagery is presented as a very promising methodology for mapping stress detection in crops. Nevertheless, the cost of the aerial platform and the requirement of qualified operators (or the existence of legal restrictions) might limit the adoption of these methodologies in development countries. For this reason, using ground-based approaches like attaching a camera to a pheno-pole might be considered a good alternative.

## Conclusions

Proper nitrogen management is crucial to conservation agriculture as evidenced by the significant yield increase recorded when the application of residues is combined with N fertilizer application as top-dressing. Quantifying the optimal quantity of stover that can be incorporated as a residue cover will beneficiate yield and will be of economic importance for the small holder farmers. This study demonstrated the potential of remote sensing tools at leaf and canopy scale to predict GY and assess leaf N content. This would enable the adjustment of N fertilizer inputs for optimizing GY, therefore making the N fertilizer applications more efficient.

In this study, leaf-based measurements proved to be good indicators of leaf N content, mostly because chlorophylls are tightly associated with leaf proteins, and thus to the N concentration. This is also reported as leaf color changes in the RGB scans. Despite performing robustly for leaf N content monitoring, operating at the leaf scale is time-consuming and its application in large scale studies and in assessing GY is limited. The other limitation to consider is that the selection of the leaves to be assessed can be subjective. On the other hand, canopy based RGB indices were shown to be effective measurements of crop density, as a direct effect of the soil N availability in the plot. As a low-cost tool in comparison to the more specialized leaf-clip sensors, digital photography is a promising approach for precision agriculture and crop management. Regarding the comparison between the ground and aerial platform-based measurements, both performed very similarly in terms of assessing leaf N content and GY. The selection of the platform would depend on its costs and the skills required, but with the use of drones there is certainly an improved high-throughput capacity. Stable nitrogen isotope composition, and, despite the C4 nature of the crop, carbon isotope composition, provided relevant information of the effect of crop management conditions in maize.

## Methods

### Site description and plant material

**Figure 7:**
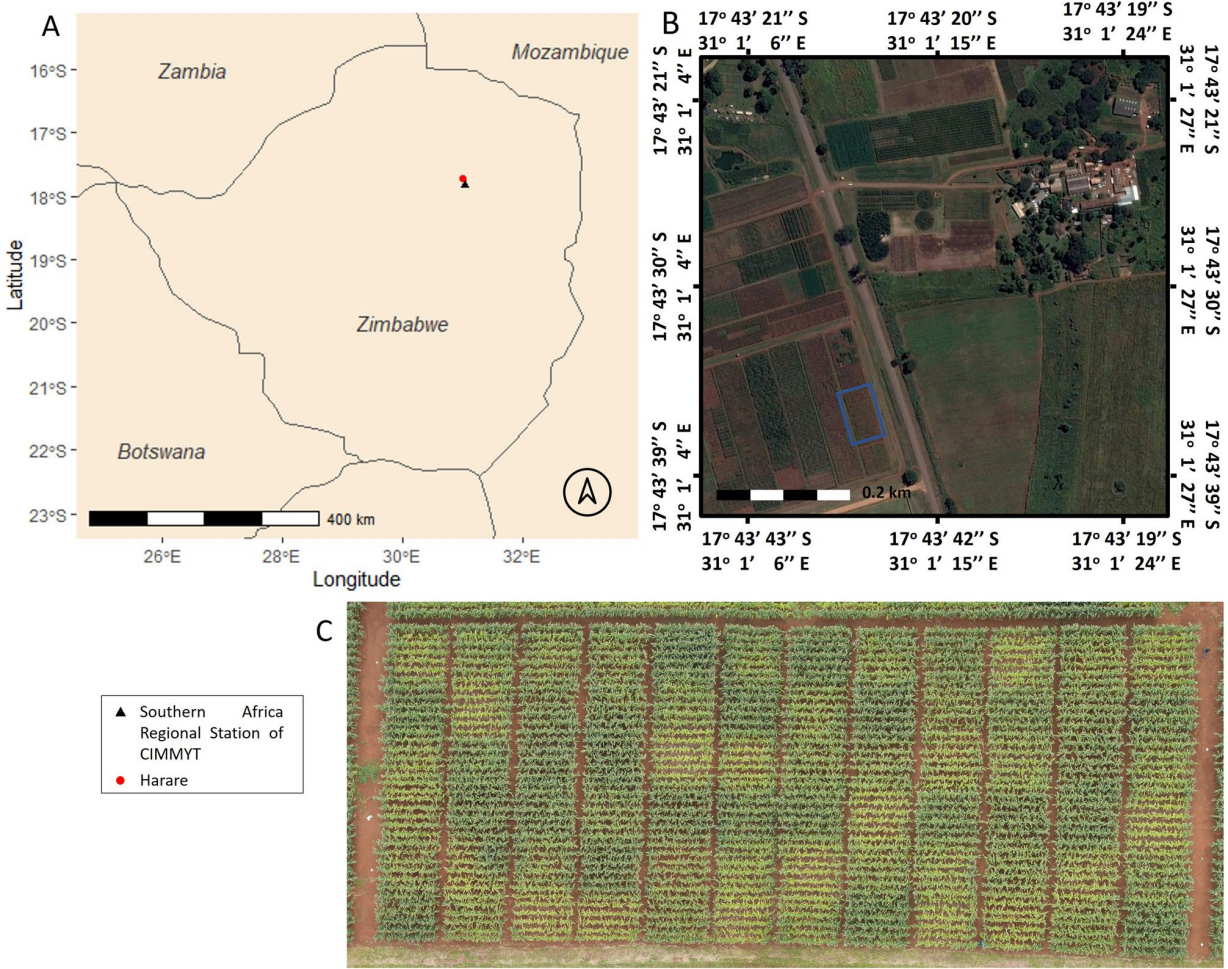
(**A**) Map of Zimbabwe with the location of Harare and the Southern Africa Regional Station of CIMMYT. (**B**) Landsat-8 satellite image of the study area acquired from DigitalGlobe using Google Earth Pro on the 28th of March 2017. (**C**) Aerial image Red–Green–Blue (RGB) orthomosaic at 30 m of the trial.

The experiment was located at the Southern Africa Regional Office of CIMMYT (International Maize and Wheat Improvement Center) located in Harare (17° 43′ 32″ S, 31° 00′ 59″ E, at an altitude of 1498 m above sea level), during the crop season 2016/2017 (Fig. 7). The soil type at the field site is characterized by a pH slightly below 6. The previously sown crop was maize with no tillage and without residue application and fertilized using compound D with 200 kg ha^−1^ ammonium nitrate (AN). The plant material used in this experiment was the commercial maize variety “PGSG3”.

### Experimental design and crop management

The experiment was arranged in a split-plot design with five replications. Maize residue management in combination with two tillage treatments and nitrogen levels were two factors of interest. The maize residue treatments were randomly assigned to main plots and nitrogen levels treatments were randomly assigned to sub-plots. Overall, 90 plots were studied (6 main treatments × 3 sub-treatments × 5 replicates). The plot size was 6 rows × 0.9 m × 6 m long (5.4 m × 6 m = 32 m^2^). No tillage was employed during the experiment, except for the first treatment plots, that were managed using conventional tillage and the application of 4 Mg ha^−1^ of residue. The other five treatments were managed without soil tillage and an increase of the residue application from 0 to 8 Mg ha^−1^ (0 Mg ha^−1^, 2 Mg ha^−1^, 4 Mg ha^−1^, 6 Mg ha^−1^, 8 Mg ha^−1^). Maize stover treatments produced by the previous crop was weighed using a hanging scale KERN® (Kern, Balingen, Germany), spread (flat) uniformly over the soil surface immediately after harvest in June at the respective rates. Three different fertilization regimens were established in order to generate a range of N soil levels in the growing conditions (Table 3).

**Table 3:** Top-dressing fertilizer treatments.

Subtreatments	Top-dressing fertilizer
0N	28 kg ha^−1^ P_2_O_5_ and 14 kg ha^−1^ K_2_0
30N	200 kg ha^−1^ Compound D (7:14:7) and 46 kg ha^−1^ AN
90N	200 kg ha^−1^ Compound D (7:14:7) and 220 kg ha^−1^ AN

Split application of top dressing was done using ammonium nitrate (AN) (34.5%N), first applied at 4 weeks after planting (WAP) and second at 7 WAP. Post emergence herbicides and hand pulling was used to control weeds. Complementary irrigation was provided when necessary to avoid unwanted drought stress.

The planting was done during the summer season 2016–2017 after receiving sufficient rainfall (20 mm received within two consecutive days). A ripper was used to open planting rows followed by hand planting. Seeds of PGS 63 were sown two seeds per station on 17th November 2016 and thinned to 1 plant per station at V3 targeting 44,444 plants ha^−1^.

### Data collection

The date of emergence was recorded when 50% of the crop emerged. At harvest maize grain and stover yield were recorded from final harvest area of 4 rows × 4 m in the middle of each plot. Maize cobs were removed manually from the stalks and weighed. Sub-samples of ten cobs were randomly selected from each plot and weighed, air-dried and shelled; moisture content was determined using a Dickey–John mini GAC moisture tester (Döscher Microwave Systems GmbH, Rellingen, Germany) and then dry weight determined (at 0.1 g precision). Maize grain yield was calculated, converted to mass ha^−1^ at 125 g kg^−1^ moisture content. Total maize stalks and leaves of each sample was weighed using a hanging scale KERN®. A sub-sample of three plants(stalks) were randomly selected from each sample and grinded using mulcher into small pieces and a representative sub-sample of approximately 500 g was collected and weighed immediate to obtain field weight then air dried. Stalk sub-sample was re-weighed after drying to determine dry weight (at 0.1 g precision).

The field data measurements with different methodologies and sensors were taken during the 5th February 2017. The crop was between the R1–R2 phenological stages.

### Leaf-clip sensors

Two different clip sensors were used in order to estimate the chlorophyll content. On the one hand, the SPAD-502 chlorophyll meter (Konica Minolta Inc., Japan) that measures the light transmitted by the plant leaf when the sensor provide light from a red LED (650 nm) and an infrared LED (940 nm). On the other, the Dualex Scientific (Force-A, Orsay, France) sensor operates with a red reference beam at 650 nm and a UV light at 375 nm. This latter sensor, besides chlorophylls a + b (Chl), it also produces relative measures of flavonoids (Flav) and anthocyanin (Anth) content and the nitrogen balance index (NBI), which is the ratio Chl/Flav related to the nitrogen and carbon allocation^17,63^. The plot measurements derived from both sensors correspond to the average of five measurements of five different leaves from five different plants. The measurements were taken from the middle portion of the leaves, a mix between the upper and the lower leaves around the cob.

### RGB images and RGB indices calculation

**Figure 8:**
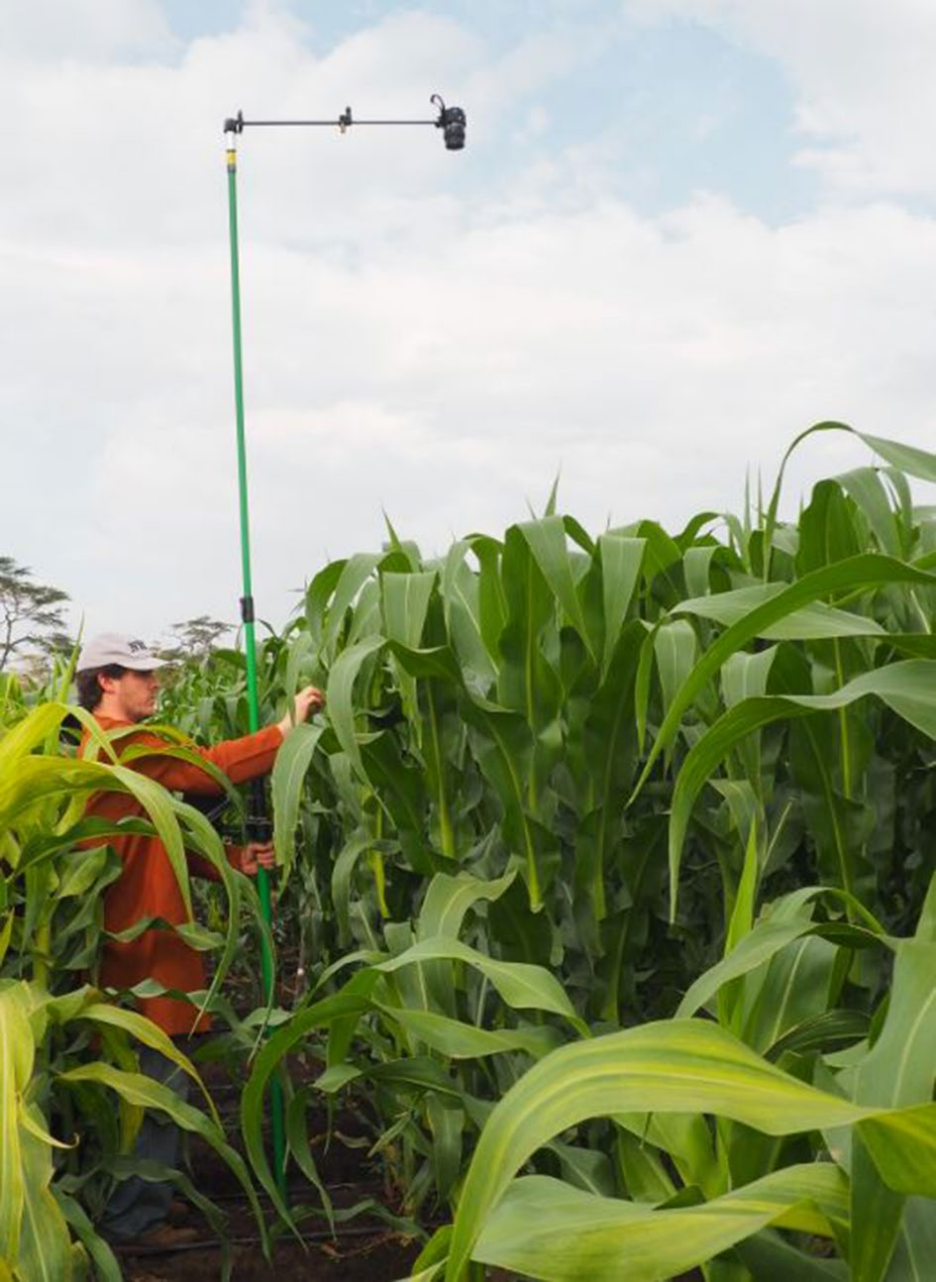
Ground level RGB canopy images system using the pheno-pole.

RGB indices were formulated from images taken at three different scales. On one side, the central part of the leaf placed just below the ear of six different plants per plot were scanned using a flatbed scanner CanonScan Lide 120 (Canon, Tokyo, Japan). At the ground level, one picture was taken per plot, holding the camera at 4 m above the plant canopy in a zenithal plane and focusing near the center of each plot using a “pheno-pole” (camera extension pole) Megaview Lite (Megaview Photomast Systems, Twello, Netherlands) made of glass fiber (Fig. 8). The conventional digital camera used was a 20.1-megapixel Sony ILCE-QX1 (Sony Corporation, Minato, Japan) with images saved in JPEG format at a resolution of 5456 × 3632 pixels. The camera was controlled remotely using a smartphone. At the aerial level, an eight rotor Mikrokopter Oktokopter XL 4S (HiSystems GmbH, Moomerland, Germany) equipped with a 16-megapixel Lumix GX7 (Panasonic, Osaka, Japan) was used and images were taken at 30 m above the ground level. Images were saved in JPEG format at a resolution of 4592 × 3448 pixels. In order to correct the effect of pitch and roll movements of the drone during the flight, an active two-servo gimbal was used to steady the camera.

The scanned images were cropped semi-automatically using the open source image analysis platform FIJI (Fiji is Just ImageJ; https://fiji.sc/Fiji) into six different images of 1176 × 1286 pixels corresponding to each section of the six leaves from six different plants. The measurements were taken from the middle portion of the leaves. For the orthomosaic reconstruction procedure with the aerial images, a 3D reconstruction model was produced using the Agisoft PhotoScan Professional software (Agisoft LLC, St. Petersburg, Russia, www.agisoft.com)^64^ by using aerial images with at least 80% overlap. Then, regions of interest corresponding to each plot were segmented and exported using the MosaicTool (Shawn C. Kefauver, https://integrativecropecophysiology.com/software-development/mosaictool/, https://gitlab.com/sckefauver/MosaicTool, University of Barcelona, Barcelona, Spain) integrated as a plugin for FIJI. Finally, segmented scans, ground images and segmented aerial images were subsequently analyzed using also the MosaicTool plugin^62^, that enables the extraction of RGB indices in relation to different color properties of potential interest^59^. Derived from the HSI (Hue–Saturation–Intensity) color space, the parameters Hue, referring to the color tint; Saturation, an indication of how much the pure color is diluted with white color; and Intensity, as an achromatic measurement of the reflected light, where extracted. In addition, the portion of pixels classified as green by their Hue values was determined by the Green Area (GA) and the Greener Area (GGA) indices. The GA corresponds to the percentage of pixels that have a Hue value between 60° and 180°. Meanwhile, the GGA is more restrictive, because it reduces the range from 80° to 180°, thus excluding the yellowish-green tones. Both indices are also used for the formulation of the crop senescence index (CSI)^61^, which provides a scaled ratio between yellow and green pixels to assess the percentage of senescent vegetation. The CSI index was calculated as follows:

1 $$ CSI = \frac{{\left( {GA - GGA} \right)}}{GA} \times 100 $$

From the CIELab and the CIELuv color space models (recommended by the International Commission on Illumination—CIE—for improved color chromaticity compared to HSI color space), the following parameters were calculated: L*, that represents lightness and is very similar than the intensity from the HSI color; the a* and u*, that represent the red green spectrum of chromaticity; and the b* and v* represent the yellow–blue color spectrum^52^. Further, besides those indices calculated with the Breedpix software, two additional indices derived from the RGB color model were calculated using the digital numbers (DN) of the red, green and blue bands. One, the normalized green–red difference index (NGRDI) is formulated very similarly than the well-known normalized difference vegetation index (NDVI), but instead of using the near-infrared information, it uses the information from the red and green bands^55^. It is formulated as follows:

2 $$ NGRDI = \frac{{\left( {Green\;DN - Red\;DN} \right)}}{{\left( {Green\;DN + Red\;DN} \right)}} $$

The other index is the triangular greenness index (TGI), that estimates chlorophyll content based on the area of a triangle with the three points corresponding to the red, green, and blue bands^56^, and it is formulated as follows:

3 $$ TGI = - 0.5 \cdot \left[ {190 \cdot \left( {Red\;DN - Green\;DN} \right) - 120 \cdot \left( {Red \;DN - Blue\; DN} \right)} \right] $$

Therefore, this set of indices was calculated at three different scales: scan, ground and aerial.

### Total nitrogen content and nitrogen and carbon stable isotope compositions

The same maize leaves scanned and used for leaf clip sensor measurements were oven dried at 70 °C for 24 h and were grounded to a fine powder using a ball mill. Then, samples of approximately 0.7 mg of dry matter were weighed into tin capsules, sealed, and then loaded into an elemental analyzer (Flash 1112 EA; ThermoFinnigan, Schwerte, Germany) coupled with an isotope ratio mass spectrometer (Delta C IRMS, ThermoFinnigan), operating in continuous flow mode. Measurements were carried out at the Scientific Facilities of the University of Barcelona. The ^13^C/^12^C ratios of plant material were expressed in composition (δ^13^C) notation^65^ as follows:

4 $$ \delta^{13} C (\permil) = \left[ {\left( {\frac{{R_{sample} }}{{R_{standard} }}} \right) - 1} \right] \times 1000 $$

in which R_sample_ refers to plant material and R_standard_ to Pee Dee Belemmite (PDB) calcium carbonate. International isotope secondary standards of a known ^13^C/^12^C ratio (IAEA CH7, polyethylene foil, IAEA CH6 sucrose and USGS 40 l-glutamic acid) were calibrated against Vienna Pee Dee Belemnite calcium carbonate (VPDB) with an analytical precision of 0.1‰. The 15N/14N ratios of plant material were also expressed in δ notation (δ^15^N) using international secondary standards of known ^15^N/^14^N ratios (IAEA N1 and IAEA N2 ammonium sulfate and IAEA NO_3_ potassium nitrate), with analytical precision of about 0.2‰.

During the same process, nitrogen content was determined through the combustion of dry matter. Nitrogen was expressed as a concentration per unit dry weight.

### Statistical analysis

Statistical analyses were conducted using the open source software, R and RStudio 1.0.44 (R Foundation for Statistical Computing, Vienna, Austria). Means and standard errors were calculated using the summarySE() function from the “Rmisc” package. Tukey’s HSD test was used to determine post hoc differences at each growing condition using the HSD.test() function from the “agricolae” package. Data for the set of physiological traits were subjected to factorial completely randomized analyses of variance (ANOVAs). to test the effects of growing conditions on the different traits studied using the anova() function with a linear model. A two-ways linear model ANOVA was used to examine the influence of the top-dressing levels (0, 30 and 90N) and the combination of tillage and residue level (CA + 4 Mg/Ha Residues and CP + 0, 2, 4, 6 and 8 Mg/Ha Residues). Differences were considered significant at *p* value ≤ 0.05. A bivariate correlation procedure was used to calculate the Pearson correlation coefficients of the different remote sensing indices against GY and leaf N content using the cor.test() function. All the chart figures were designed using the package “ggplot2”.

## Supplementary information

Supplementary Information.
