# Supplementary material for: Leaf versus whole-canopy remote sensing methodologies for crop monitoring under conservation agriculture: a case of study with maize in Zimbabwe
Source: Sci Rep. 2020 Sep 29;10:16008. doi: 10.1038/s41598-020-73110-3 (PMC7524805; doi:10.1038/s41598-020-73110-3)
Supplement: Supplementary file 1 — Supplementary Information. [file 41598_2020_73110_MOESM1_ESM.docx]

**Leaf vs. Whole-Canopy Remote Sensing Methodologies for Crop Monitoring Under Conservation Agriculture. A Case Study of Maize in Zimbabwe**

Adrian Gracia-Romero^1^, Shawn C. Kefauver^1^, Omar Vergara-Díaz1, Esnath Hamadziripi^2^, Mainassara A. Zaman-Allah^2^, Boddupalli M. Prassana^2^, Christian Thierfelder^2^, Jill E. Cairns^2^ and José L. Araus^1^*

^1^ Integrative Crop Ecophysiology Group, Plant Physiology Section, Faculty of Biology, University of Barcelona, Barcelona, Spain and AGROTECNIO (Center for Research in Agrotechnology), Av. Rovira Roure 191, 25198, Lleida, Spain, ^2^ International Maize and Wheat Improvement Center, CIMMYT Southern Africa Regional Office, Harare, Zimbabwe

| **Supplemental Table 1.** Effect of the combination of reside levels and treatments (Across R+T) and the top-dressing levels (Across TD) on the leaf pigments readings. Values are mean ± standard error of three replicates per cultivar. Different letters (a, b, c, d) indicate significant differences between cultivars within each growing condition according to Fisher’s LSD test. | | | | | | | | | | | | | | | | | | | | | | | | | | | | | |
| --- | --- | --- | --- | --- | --- | --- | --- | --- | --- | --- | --- | --- | --- | --- | --- | --- | --- | --- | --- | --- | --- | --- | --- | --- | --- | --- | --- | --- | --- |
| **Tillage** | | ***Across R+T*** | | | | ***Conventional Tillage*** | | | | ***Conservation Agriculture*** | | | | | | | | | | | | | | | | | | | |
| ***Residue level*** | |  |  |  |  | ***4 Mg ha^-1^*** | | | | ***0 Mg ha^-1^*** | | | | ***2 Mg ha^-1^*** | | | | ***4 Mg ha^-1^*** | | | | ***6 Mg ha^-1^*** | | | | ***8 Mg ha^-1^*** | | | |
| ***SPAD*** |  |  |  |  |  |  |  |  |  |  |  |  |  |  |  |  |  |  |  |  |  |  |  |  |  |  |  |  |  |
|  | ***Across TD*** |  |  |  |  | 45.89 | ± | 3.42 | a | 43.93 | ± | 1.84 | a | 44.19 | ± | 2.95 | a | 38.10 | ± | 2.56 | a | 39.98 | ± | 2.68 | a | 38.19 | ± | 1.99 | a |
|  | ***0 N*** | 34.43 | ± | 1.52 | c | 41.49 | ± | 7.00 | ab | 38.36 | ± | 2.35 | ab | 34.17 | ± | 1.12 | ab | 29.73 | ± | 1.30 | b | 29.65 | ± | 2.11 | b | 33.16 | ± | 3.28 | ab |
|  | ***30 N*** | 42.40 | ± | 1.59 | b | 44.44 | ± | 5.38 | ab | 43.43 | ± | 3.02 | ab | 46.55 | ± | 2.65 | ab | 42.27 | ± | 5.11 | ab | 39.59 | ± | 3.30 | ab | 38.14 | ± | 3.91 | ab |
|  | ***90 N*** | 48.31 | ± | 1.68 | a | 51.75 | ± | 5.54 | a | 49.99 | ± | 2.02 | a | 51.85 | ± | 6.48 | a | 42.32 | ± | 3.82 | ab | 50.71 | ± | 2.20 | a | 43.27 | ± | 1.70 | ab |
|  |  |  |  |  |  |  |  |  |  |  |  |  |  |  |  |  |  |  |  |  |  |  |  |  |  |  |  |  |  |
| ***Chl*** | ***Across TD*** |  |  |  |  | 33.61 | ± | 1.98 | a | 33.34 | ± | 1.63 | a | 31.97 | ± | 2.16 | a | 28.92 | ± | 2.19 | a | 31.82 | ± | 2.73 | a | 29.17 | ± | 2.41 | a |
|  | ***0 N*** | 24.48 | ± | 1.15 | c | 28.83 | ± | 3.69 | abcd | 27.15 | ± | 0.94 | abcd | 23.31 | ± | 1.88 | cd | 21.42 | ± | 2.13 | d | 22.08 | ± | 2.39 | d | 24.08 | ± | 4.36 | bcd |
|  | ***30 N*** | 31.66 | ± | 1.35 | b | 32.49 | ± | 2.18 | abcd | 32.12 | ± | 1.38 | abcd | 34.32 | ± | 2.58 | abcd | 30.51 | ± | 3.84 | abcd | 31.51 | ± | 4.40 | abcd | 29.01 | ± | 5.24 | abcd |
|  | ***90 N*** | 38.28 | ± | 0.99 | a | 39.50 | ± | 2.84 | ab | 40.74 | ± | 1.26 | a | 38.27 | ± | 2.96 | abc | 34.84 | ± | 2.72 | abcd | 41.87 | ± | 2.02 | a | 34.44 | ± | 0.97 | abcd |
|  |  |  |  |  |  |  |  |  |  |  |  |  |  |  |  |  |  |  |  |  |  |  |  |  |  |  |  |  |  |
| ***Flav*** | ***Across TD*** |  |  |  |  | 1.46 | ± | 0.03 | b | 1.48 | ± | 0.04 | ab | 1.51 | ± | 0.04 | ab | 1.61 | ± | 0.04 | a | 1.59 | ± | 0.04 | ab | 1.61 | ± | 0.04 | a |
|  | ***0 N*** | 1.59 | ± | 0.03 | b | 1.54 | ± | 0.05 | a | 1.50 | ± | 0.07 | a | 1.65 | ± | 0.06 | a | 1.66 | ± | 0.06 | a | 1.61 | ± | 0.08 | a | 1.59 | ± | 0.10 | a |
|  | ***30 N*** | 1.57 | ± | 0.03 | a | 1.44 | ± | 0.05 | a | 1.58 | ± | 0.06 | a | 1.45 | ± | 0.02 | a | 1.61 | ± | 0.08 | a | 1.66 | ± | 0.06 | a | 1.67 | ± | 0.08 | a |
|  | ***90 N*** | 1.47 | ± | 0.02 | a | 1.41 | ± | 0.04 | a | 1.37 | ± | 0.04 | a | 1.43 | ± | 0.05 | a | 1.55 | ± | 0.04 | a | 1.49 | ± | 0.07 | a | 1.59 | ± | 0.02 | a |
|  |  |  |  |  |  |  |  |  |  |  |  |  |  |  |  |  |  |  |  |  |  |  |  |  |  |  |  |  |  |
| ***Anth*** | ***Across TD*** |  |  |  |  | 0.15 | ± | 0.01 | a | 0.14 | ± | 0.01 | a | 0.16 | ± | 0.01 | a | 0.16 | ± | 0.01 | a | 0.16 | ± | 0.01 | a | 0.17 | ± | 0.01 | a |
|  | ***0 N*** | 0.18 | ± | 0.01 | c | 0.17 | ± | 0.01 | abc | 0.16 | ± | 0.01 | abc | 0.18 | ± | 0.00 | ab | 0.20 | ± | 0.01 | a | 0.20 | ± | 0.01 | a | 0.18 | ± | 0.02 | ab |
|  | ***30 N*** | 0.16 | ± | 0.00 | b | 0.14 | ± | 0.01 | abc | 0.14 | ± | 0.01 | abc | 0.16 | ± | 0.01 | abc | 0.15 | ± | 0.01 | abc | 0.17 | ± | 0.01 | abc | 0.17 | ± | 0.01 | abc |
|  | ***90 N*** | 0.13 | ± | 0.01 | a | 0.13 | ± | 0.02 | bc | 0.12 | ± | 0.01 | c | 0.13 | ± | 0.01 | bc | 0.13 | ± | 0.01 | bc | 0.12 | ± | 0.01 | c | 0.14 | ± | 0.02 | abc |
|  |  |  |  |  |  |  |  |  |  |  |  |  |  |  |  |  |  |  |  |  |  |  |  |  |  |  |  |  |  |
| ***NBI*** | ***Across TD*** |  |  |  |  | 24.15 | ± | 1.68 | a | 23.19 | ± | 1.44 | a | 21.94 | ± | 1.94 | a | 18.50 | ± | 1.71 | a | 20.92 | ± | 2.20 | a | 18.92 | ± | 2.07 | a |
|  | ***0 N*** | 15.97 | ± | 1.05 | c | 19.65 | ± | 2.93 | abc | 18.47 | ± | 1.30 | abc | 14.07 | ± | 1.35 | c | 13.31 | ± | 1.76 | c | 13.93 | ± | 1.70 | c | 16.37 | ± | 4.62 | bc |
|  | ***30 N*** | 20.88 | ± | 1.16 | b | 23.39 | ± | 1.82 | abc | 21.00 | ± | 0.91 | abc | 24.28 | ± | 2.04 | abc | 19.61 | ± | 3.57 | abc | 19.34 | ± | 3.48 | abc | 17.66 | ± | 4.22 | abc |
|  | ***90 N*** | 26.97 | ± | 0.94 | a | 29.42 | ± | 2.36 | ab | 30.12 | ± | 0.59 | a | 27.47 | ± | 2.97 | ab | 22.57 | ± | 1.93 | abc | 29.50 | ± | 2.10 | ab | 22.74 | ± | 0.61 | abc |
|  |  |  |  |  |  |  |  |  |  |  |  |  |  |  |  |  |  |  |  |  |  |  |  |  |  |  |  |  |  |


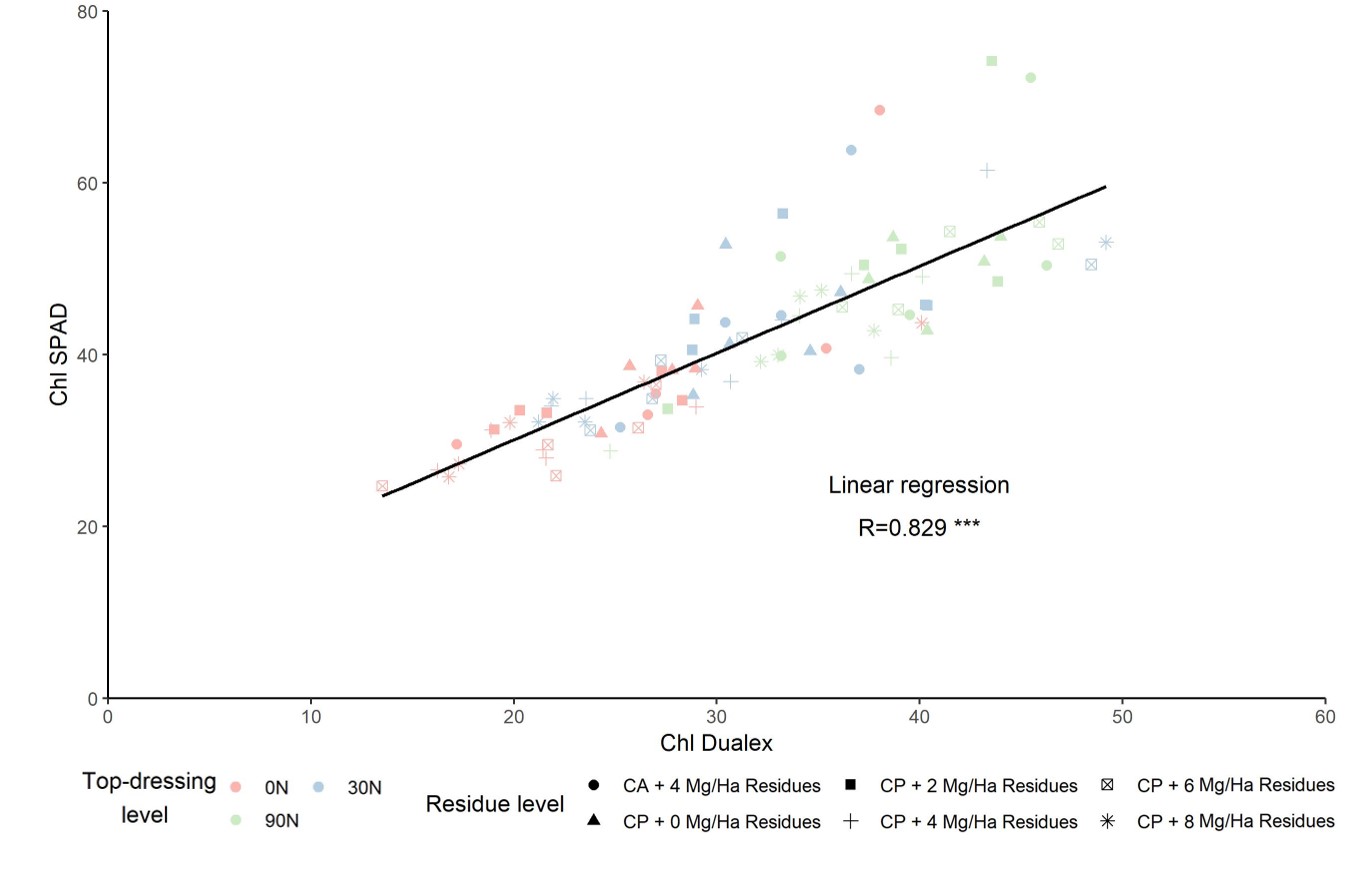


**Supplemental Figure 1.** Relationship between the chlorophyll readings measured by the SPAD and the Dualex sensors. Correlations were studied across the 90 plots from all the growing conditions. Significance levels of the correlations and ANOVAs: ns, P > 0.05; *, P < 0.05; **, P < 0.01; ***, P < 0.001.

**Supplemental Table 2.** Effect of the combination of reside levels and treatments (Across R+T) and the top-dressing levels (Across TD) on the RGB indexes derived from the leaf scans. Values are mean ± standard error of three replicates per cultivar. Different letters (a, b, c, d, e, f) indicate significant differences between cultivars within each growing condition according to Fisher’s LSD test.

| **Tillage** | | ***Across T+R*** | | | | ***Conventional Tillage*** | | | | ***Conservation Agriculture*** | | | | | | | | | | | | | | | | | | | |
| --- | --- | --- | --- | --- | --- | --- | --- | --- | --- | --- | --- | --- | --- | --- | --- | --- | --- | --- | --- | --- | --- | --- | --- | --- | --- | --- | --- | --- | --- |
| ***Residue level*** | |  |  |  |  | ***4 Mg/ha*** | | | | ***0 Mg/ha*** | | | | ***2 Mg/ha*** | | | | ***4 Mg/ha*** | | | | ***6 Mg/ha*** | | | | ***8 Mg/ha*** | | | |
|  |  |  |  |  |  |  |  |  |  |  |  |  |  |  |  |  |  |  |  |  |  |  |  |  |  |  |  |  |  |
| ***Hue*** | ***Across TD*** |  |  |  |  | 88.20 | ± | 0.52 | a | 87.69 | ± | 0.60 | a | 88.69 | ± | 0.57 | a | 88.49 | ± | 0.79 | a | 87.86 | ± | 0.88 | a | 89.05 | ± | 1.04 | a |
|  |  |  |  |  |  |  |  |  |  |  |  |  |  |  |  |  |  |  |  |  |  |  |  |  |  |  |  |  |  |
|  | ***0 N*** | 86.24 | ± | 0.55 | a | 87.29 | ± | 0.76 | abcd | 86.51 | ± | 0.98 | bcd | 86.78 | ± | 1.20 | bcd | 85.87 | ± | 1.09 | cd | 84.26 | ± | 1.29 | d | 86.73 | ± | 2.39 | bcd |
|  | ***30 N*** | 88.65 | ± | 0.32 | b | 88.50 | ± | 0.48 | abcd | 87.29 | ± | 0.83 | abcd | 90.04 | ± | 0.59 | abc | 88.30 | ± | 0.88 | abcd | 88.99 | ± | 0.84 | abc | 88.80 | ± | 0.85 | abc |
|  | ***90 N*** | 90.09 | ± | 0.43 | c | 88.81 | ± | 1.30 | abc | 89.26 | ± | 1.08 | abc | 89.26 | ± | 0.48 | abc | 91.29 | ± | 0.94 | a | 90.33 | ± | 0.82 | ab | 91.61 | ± | 1.34 | a |
|  |  |  |  |  |  |  |  |  |  |  |  |  |  |  |  |  |  |  |  |  |  |  |  |  |  |  |  |  |  |
| ***Intensity*** | ***Across TD*** |  |  |  |  | 0.32 | ± | 0.01 | a | 0.32 | ± | 0.01 | a | 0.32 | ± | 0.01 | a | 0.33 | ± | 0.01 | a | 0.33 | ± | 0.01 | a | 0.32 | ± | 0.01 | a |
|  |  |  |  |  |  |  |  |  |  |  |  |  |  |  |  |  |  |  |  |  |  |  |  |  |  |  |  |  |  |
|  | ***0 N*** | 0.35 | ± | 0.01 | a | 0.35 | ± | 0.01 | abcd | 0.34 | ± | 0.01 | abcde | 0.35 | ± | 0.02 | abc | 0.36 | ± | 0.01 | ab | 0.38 | ± | 0.01 | a | 0.35 | ± | 0.02 | abc |
|  | ***30 N*** | 0.32 | ± | 0.00 | b | 0.31 | ± | 0.01 | cdef | 0.32 | ± | 0.01 | bcdef | 0.30 | ± | 0.01 | def | 0.33 | ± | 0.01 | bcdef | 0.32 | ± | 0.01 | bcdef | 0.31 | ± | 0.01 | bcdef |
|  | ***90 N*** | 0.29 | ± | 0.00 | c | 0.30 | ± | 0.02 | ef | 0.29 | ± | 0.01 | ef | 0.30 | ± | 0.01 | ef | 0.29 | ± | 0.01 | f | 0.30 | ± | 0.01 | ef | 0.29 | ± | 0.01 | f |
|  |  |  |  |  |  |  |  |  |  |  |  |  |  |  |  |  |  |  |  |  |  |  |  |  |  |  |  |  |  |
| ***Saturation*** | ***Across TD*** |  |  |  |  | 0.40 | ± | 0.01 | a | 0.39 | ± | 0.01 | a | 0.40 | ± | 0.02 | a | 0.42 | ± | 0.02 | a | 0.42 | ± | 0.02 | a | 0.43 | ± | 0.02 | a |
|  |  |  |  |  |  |  |  |  |  |  |  |  |  |  |  |  |  |  |  |  |  |  |  |  |  |  |  |  |  |
|  | ***0 N*** | 0.46 | ± | 0.01 | a | 0.43 | ± | 0.02 | abcd | 0.42 | ± | 0.01 | abcd | 0.47 | ± | 0.02 | ab | 0.48 | ± | 0.02 | a | 0.47 | ± | 0.02 | ab | 0.49 | ± | 0.03 | a |
|  | ***30 N*** | 0.41 | ± | 0.01 | b | 0.40 | ± | 0.01 | bcde | 0.41 | ± | 0.01 | bcde | 0.38 | ± | 0.02 | cde | 0.41 | ± | 0.03 | bcde | 0.42 | ± | 0.02 | abcde | 0.44 | ± | 0.02 | abc |
|  | ***90 N*** | 0.36 | ± | 0.01 | c | 0.38 | ± | 0.02 | cde | 0.35 | ± | 0.01 | e | 0.37 | ± | 0.02 | de | 0.37 | ± | 0.03 | cde | 0.36 | ± | 0.01 | de | 0.36 | ± | 0.02 | de |
|  |  |  |  |  |  |  |  |  |  |  |  |  |  |  |  |  |  |  |  |  |  |  |  |  |  |  |  |  |  |
| ***GA*** | ***Across TD*** |  |  |  |  | 0.98 | ± | 0.00 | a | 0.98 | ± | 0.00 | a | 0.98 | ± | 0.00 | a | 0.99 | ± | 0.00 | a | 0.98 | ± | 0.01 | a | 0.98 | ± | 0.00 | a |
|  |  |  |  |  |  |  |  |  |  |  |  |  |  |  |  |  |  |  |  |  |  |  |  |  |  |  |  |  |  |
|  | ***0 N*** | 0.97 | ± | 0.00 | b | 0.98 | ± | 0.00 | ab | 0.97 | ± | 0.00 | bc | 0.98 | ± | 0.01 | ab | 0.99 | ± | 0.01 | ab | 0.95 | ± | 0.01 | c | 0.98 | ± | 0.01 | ab |
|  | ***30 N*** | 0.98 | ± | 0.00 | a | 0.98 | ± | 0.00 | ab | 0.97 | ± | 0.01 | abc | 0.98 | ± | 0.00 | ab | 0.99 | ± | 0.00 | ab | 0.99 | ± | 0.01 | ab | 0.98 | ± | 0.01 | ab |
|  | ***90 N*** | 0.99 | ± | 0.00 | a | 0.98 | ± | 0.00 | ab | 0.99 | ± | 0.00 | ab | 0.99 | ± | 0.00 | ab | 0.99 | ± | 0.00 | a | 0.99 | ± | 0.00 | ab | 0.99 | ± | 0.00 | ab |
|  |  |  |  |  |  |  |  |  |  |  |  |  |  |  |  |  |  |  |  |  |  |  |  |  |  |  |  |  |  |
| ***GGA*** | ***Across TD*** |  |  |  |  | 0.85 | ± | 0.01 | a | 0.84 | ± | 0.01 | a | 0.84 | ± | 0.02 | a | 0.84 | ± | 0.02 | a | 0.84 | ± | 0.02 | a | 0.85 | ± | 0.02 | a |
|  |  |  |  |  |  |  |  |  |  |  |  |  |  |  |  |  |  |  |  |  |  |  |  |  |  |  |  |  |  |
|  | ***0 N*** | 0.80 | ± | 0.01 | b | 0.86 | ± | 0.01 | abc | 0.82 | ± | 0.02 | abcd | 0.80 | ± | 0.04 | cd | 0.77 | ± | 0.04 | d | 0.77 | ± | 0.03 | d | 0.80 | ± | 0.04 | bcd |
|  | ***30 N*** | 0.86 | ± | 0.00 | a | 0.86 | ± | 0.01 | abc | 0.84 | ± | 0.01 | abcd | 0.87 | ± | 0.00 | ab | 0.87 | ± | 0.01 | abc | 0.87 | ± | 0.01 | abc | 0.87 | ± | 0.01 | abc |
|  | ***90 N*** | 0.87 | ± | 0.01 | a | 0.85 | ± | 0.02 | abcd | 0.87 | ± | 0.02 | ab | 0.86 | ± | 0.01 | abc | 0.89 | ± | 0.01 | a | 0.88 | ± | 0.01 | a | 0.88 | ± | 0.01 | a |
|  |  |  |  |  |  |  |  |  |  |  |  |  |  |  |  |  |  |  |  |  |  |  |  |  |  |  |  |  |  |
| ***CSI*** | ***Across TD*** |  |  |  |  | 12.98 | ± | 0.74 | a | 13.75 | ± | 0.97 | a | 14.18 | ± | 1.40 | a | 14.80 | ± | 1.89 | a | 13.75 | ± | 1.29 | a | 13.37 | ± | 1.49 | a |
|  |  |  |  |  |  |  |  |  |  |  |  |  |  |  |  |  |  |  |  |  |  |  |  |  |  |  |  |  |  |
|  | ***0 N*** | 17.77 | ± | 1.21 | a | 12.44 | ± | 1.08 | bcde | 15.94 | ± | 1.75 | abcde | 18.90 | ± | 3.35 | abc | 21.91 | ± | 4.09 | a | 19.23 | ± | 2.31 | ab | 18.23 | ± | 3.63 | abcd |
|  | ***30 N*** | 12.11 | ± | 0.35 | b | 12.49 | ± | 0.77 | bcde | 14.11 | ± | 1.33 | bcde | 11.09 | ± | 0.18 | e | 12.08 | ± | 1.00 | cde | 11.42 | ± | 0.50 | de | 11.48 | ± | 0.33 | de |
|  | ***90 N*** | 11.53 | ± | 0.51 | b | 14.02 | ± | 1.90 | bcde | 11.20 | ± | 1.48 | e | 12.55 | ± | 0.79 | bcde | 10.42 | ± | 0.73 | e | 10.60 | ± | 0.80 | e | 10.40 | ± | 0.96 | e |
|  |  |  |  |  |  |  |  |  |  |  |  |  |  |  |  |  |  |  |  |  |  |  |  |  |  |  |  |  |  |
| ***Lightness*** | ***Across TD*** |  |  |  |  | 43.62 | ± | 1.54 | a | 43.50 | ± | 1.11 | a | 43.66 | ± | 1.41 | a | 45.29 | ± | 1.63 | a | 45.60 | ± | 1.65 | a | 44.48 | ± | 1.72 | a |
|  |  |  |  |  |  |  |  |  |  |  |  |  |  |  |  |  |  |  |  |  |  |  |  |  |  |  |  |  |  |
|  | ***0 N*** | 49.55 | ± | 0.88 | a | 48.09 | ± | 1.82 | abc | 46.70 | ± | 1.51 | abcd | 49.76 | ± | 1.88 | ab | 50.68 | ± | 2.03 | ab | 52.50 | ± | 1.72 | a | 49.55 | ± | 3.48 | ab |
|  | ***30 N*** | 43.66 | ± | 0.72 | b | 42.37 | ± | 1.88 | cde | 44.41 | ± | 1.57 | bcde | 41.11 | ± | 1.21 | de | 45.61 | ± | 2.05 | abcde | 44.13 | ± | 1.86 | bcde | 44.30 | ± | 2.00 | bcde |
|  | ***90 N*** | 39.87 | ± | 0.72 | c | 40.40 | ± | 3.15 | de | 39.39 | ± | 1.13 | e | 40.12 | ± | 1.34 | de | 39.58 | ± | 2.06 | e | 40.16 | ± | 1.53 | de | 39.60 | ± | 1.65 | e |
|  |  |  |  |  |  |  |  |  |  |  |  |  |  |  |  |  |  |  |  |  |  |  |  |  |  |  |  |  |  |
| ***a**** | ***Across TD*** |  |  |  |  | -22.57 | ± | 0.82 | a | -21.94 | ± | 0.57 | a | -22.74 | ± | 0.84 | a | -23.84 | ± | 0.83 | a | -23.47 | ± | 0.73 | a | -23.92 | ± | 0.82 | a |
|  |  |  |  |  |  |  |  |  |  |  |  |  |  |  |  |  |  |  |  |  |  |  |  |  |  |  |  |  |  |
|  | ***0 N*** | -25.55 | ± | 0.39 | c | -24.83 | ± | 1.21 | defg | -23.57 | ± | 0.50 | bcdefg | -26.19 | ± | 0.76 | fg | -26.52 | ± | 0.78 | g | -25.85 | ± | 0.52 | efg | -26.31 | ± | 1.39 | g |
|  | ***30 N*** | -23.02 | ± | 0.41 | b | -22.27 | ± | 0.99 | abcde | -22.58 | ± | 0.50 | abcdef | -21.57 | ± | 0.83 | abcd | -23.61 | ± | 1.29 | bcdefg | -23.82 | ± | 1.19 | bcdefg | -24.27 | ± | 1.06 | cdefg |
|  | ***90 N*** | -20.67 | ± | 0.43 | a | -20.60 | ± | 1.52 | ab | -19.66 | ± | 0.91 | a | -20.46 | ± | 1.22 | ab | -21.39 | ± | 1.32 | abcd | -20.75 | ± | 0.80 | abc | -21.16 | ± | 0.82 | abc |
|  |  |  |  |  |  |  |  |  |  |  |  |  |  |  |  |  |  |  |  |  |  |  |  |  |  |  |  |  |  |
| ***b**** | ***Across TD*** |  |  |  |  | 35.53 | ± | 1.57 | a | 34.90 | ± | 1.19 | a | 35.56 | ± | 1.62 | a | 37.59 | ± | 1.88 | a | 37.55 | ± | 1.82 | a | 37.46 | ± | 2.03 | a |
|  |  |  |  |  |  |  |  |  |  |  |  |  |  |  |  |  |  |  |  |  |  |  |  |  |  |  |  |  |  |
|  | ***0 N*** | 42.23 | ± | 0.99 | a | 39.75 | ± | 2.32 | abcd | 38.48 | ± | 1.39 | abcde | 42.71 | ± | 1.72 | abc | 44.18 | ± | 2.05 | ab | 44.70 | ± | 2.08 | a | 43.54 | ± | 3.96 | abc |
|  | ***30 N*** | 35.83 | ± | 0.80 | b | 34.64 | ± | 1.64 | def | 36.13 | ± | 1.28 | cdef | 32.59 | ± | 1.48 | def | 37.09 | ± | 2.59 | abcdef | 36.76 | ± | 2.20 | bcdef | 37.74 | ± | 2.22 | abcdef |
|  | ***90 N*** | 31.24 | ± | 0.80 | c | 32.19 | ± | 3.22 | def | 30.09 | ± | 1.40 | f | 31.38 | ± | 1.75 | ef | 31.51 | ± | 2.47 | ef | 31.18 | ± | 1.53 | ef | 31.10 | ± | 1.79 | ef |
|  |  |  |  |  |  |  |  |  |  |  |  |  |  |  |  |  |  |  |  |  |  |  |  |  |  |  |  |  |  |
| ***u**** | ***Across TD*** |  |  |  |  | -15.70 | ± | 0.71 | a | -15.02 | ± | 0.48 | a | -15.88 | ± | 0.73 | a | -16.87 | ± | 0.67 | a | -16.36 | ± | 0.56 | a | -16.98 | ± | 0.64 | a |
|  |  |  |  |  |  |  |  |  |  |  |  |  |  |  |  |  |  |  |  |  |  |  |  |  |  |  |  |  |  |
|  | ***0 N*** | -17.99 | ± | 0.34 | c | -17.67 | ± | 1.08 | defg | -16.29 | ± | 0.45 | abcdefg | -18.66 | ± | 0.84 | efg | -18.81 | ± | 0.70 | g | -17.74 | ± | 0.32 | defg | -18.75 | ± | 1.03 | fg |
|  | ***30 N*** | -16.18 | ± | 0.37 | b | -15.48 | ± | 0.92 | abcde | -15.51 | ± | 0.41 | abcdef | -15.04 | ± | 0.74 | abcd | -16.72 | ± | 1.07 | bcdefg | -16.98 | ± | 1.08 | bcdefg | -17.34 | ± | 0.90 | cdefg |
|  | ***90 N*** | -14.23 | ± | 0.39 | a | -13.94 | ± | 1.26 | ab | -13.24 | ± | 0.87 | a | -13.95 | ± | 1.15 | ab | -15.07 | ± | 1.15 | abcd | -14.35 | ± | 0.69 | abc | -14.86 | ± | 0.67 | abcd |
|  |  |  |  |  |  |  |  |  |  |  |  |  |  |  |  |  |  |  |  |  |  |  |  |  |  |  |  |  |  |
| ***v**** | ***Across TD*** |  |  |  |  | 38.81 | ± | 1.85 | a | 38.18 | ± | 1.37 | a | 38.85 | ± | 1.83 | a | 41.09 | ± | 2.10 | a | 41.16 | ± | 2.05 | a | 40.66 | ± | 2.24 | a |
|  |  |  |  |  |  |  |  |  |  |  |  |  |  |  |  |  |  |  |  |  |  |  |  |  |  |  |  |  |  |
|  | ***0 N*** | 46.35 | ± | 1.09 | a | 43.96 | ± | 2.54 | abcd | 42.26 | ± | 1.65 | abcde | 46.90 | ± | 1.98 | abc | 48.31 | ± | 2.33 | ab | 49.36 | ± | 2.22 | a | 47.31 | ± | 4.40 | abc |
|  | ***30 N*** | 39.09 | ± | 0.91 | b | 37.62 | ± | 2.06 | def | 39.51 | ± | 1.60 | cdef | 35.57 | ± | 1.65 | def | 40.86 | ± | 2.85 | abcdef | 40.09 | ± | 2.48 | bcdef | 40.86 | ± | 2.53 | abcdef |
|  | ***90 N*** | 33.94 | ± | 0.91 | c | 34.84 | ± | 3.78 | ef | 32.76 | ± | 1.57 | f | 34.10 | ± | 1.96 | ef | 34.10 | ± | 2.75 | ef | 34.04 | ± | 1.82 | ef | 33.81 | ± | 2.03 | ef |
|  |  |  |  |  |  |  |  |  |  |  |  |  |  |  |  |  |  |  |  |  |  |  |  |  |  |  |  |  |  |
| ***NGRDI*** | ***Across TD*** |  |  |  |  | 0.17 | ± | 0.00 | a | 0.18 | ± | 0.01 | a | 0.17 | ± | 0.00 | a | 0.17 | ± | 0.00 | a | 0.17 | ± | 0.00 | a | 0.18 | ± | 0.00 | a |
|  |  |  |  |  |  |  |  |  |  |  |  |  |  |  |  |  |  |  |  |  |  |  |  |  |  |  |  |  |  |
|  | ***0 N*** | 0.17 | ± | 0.00 | a | 0.18 | ± | 0.01 | a | 0.17 | ± | 0.01 | a | 0.17 | ± | 0.01 | a | 0.16 | ± | 0.01 | a | 0.18 | ± | 0.01 | a | 0.18 | ± | 0.00 | a |
|  | ***30 N*** | 0.17 | ± | 0.00 | a | 0.17 | ± | 0.01 | a | 0.17 | ± | 0.01 | a | 0.17 | ± | 0.01 | a | 0.17 | ± | 0.01 | a | 0.17 | ± | 0.00 | a | 0.18 | ± | 0.00 | a |
|  | ***90 N*** | 0.18 | ± | 0.00 | a | 0.17 | ± | 0.01 | a | 0.19 | ± | 0.01 | a | 0.18 | ± | 0.01 | a | 0.17 | ± | 0.01 | a | 0.17 | ± | 0.01 | a | 0.18 | ± | 0.01 | a |
|  |  |  |  |  |  |  |  |  |  |  |  |  |  |  |  |  |  |  |  |  |  |  |  |  |  |  |  |  |  |
| ***TGI*** | ***Across TD*** |  |  |  |  | 5367.53 | ± | 251.93 | a | 5098.91 | ± | 301.76 | a | 4858.06 | ± | 267.68 | a | 4849.24 | ± | 236.37 | a | 4872.27 | ± | 276.06 | a | 5190.20 | ± | 274.66 | a |
|  |  |  |  |  |  |  |  |  |  |  |  |  |  |  |  |  |  |  |  |  |  |  |  |  |  |  |  |  |  |
|  | ***0 N*** | 4774.37 | ± | 189.69 | a | 5097.73 | ± | 261.89 | a | 5360.83 | ± | 741.91 | a | 4505.78 | ± | 509.64 | a | 4230.52 | ± | 219.94 | a | 4455.66 | ± | 340.74 | a | 4995.72 | ± | 529.38 | a |
|  | ***30 N*** | 5281.80 | ± | 198.96 | a | 5729.02 | ± | 581.19 | a | 4785.34 | ± | 504.53 | a | 5095.21 | ± | 455.71 | a | 5317.83 | ± | 520.17 | a | 5073.71 | ± | 519.82 | a | 5689.67 | ± | 456.19 | a |
|  | ***90 N*** | 5061.93 | ± | 169.22 | a | 5275.82 | ± | 455.08 | a | 5150.56 | ± | 329.73 | a | 4973.17 | ± | 486.36 | a | 4999.38 | ± | 333.89 | a | 5087.43 | ± | 590.56 | a | 4885.21 | ± | 456.83 | a |
|  |  |  |  |  |  |  |  |  |  |  |  |  |  |  |  |  |  |  |  |  |  |  |  |  |  |  |  |  |  |

**Supplemental Table 3.** Effect of the combination of reside levels and treatments (Across R+T) and the top-dressing levels (Across TD) on the RGB indexes derived from the ground and aerial RGB images. Values are mean ± standard error of five replicates per cultivar. Different letters (a, b, c, d, e, f) indicate significant differences between cultivars within each growing condition according to Tukey’s HSD test.

|  |  | |  |  | | |  |  | |  | **RGB ground** | | | | | | | | | | | | | | | | | | | | | | | | | | | | | | | | | | | | | | | | | | |  | |  |  |  |  |  |  |  |  |
| --- | --- | --- | --- | --- | --- | --- | --- | --- | --- | --- | --- | --- | --- | --- | --- | --- | --- | --- | --- | --- | --- | --- | --- | --- | --- | --- | --- | --- | --- | --- | --- | --- | --- | --- | --- | --- | --- | --- | --- | --- | --- | --- | --- | --- | --- | --- | --- | --- | --- | --- | --- | --- | --- | --- | --- | --- | --- | --- | --- | --- | --- | --- | --- |
| ***Index*** | | | **Tillage** | ***Across T + R*** | | | | | | | ***Conventional Tillage*** | | | | | | | | ***Conservation Agriculture*** | | | | | | | | | | | | | | | | | | | | | | | | | | | | | | | | | | |  | |  |  |  |  |  |  |  |  |
|  |  |  | ***Residue level*** |  |  |  |  |  |  |  | ***4 Mg/ha*** | | | | | | | | ***0 Mg/ha*** | | | | | | | ***2 Mg/ha*** | | | | | | | ***4 Mg/ha*** | | | | | | | | ***6 Mg/ha*** | | | | | | ***8 Mg/ha*** | | | | | | |  | |  |  |  |  |  |  |  |  |
|  |  | |  |  | | |  |  | |  |  | | |  |  | |  | |  | |  | |  |  | |  | |  |  | | |  |  | |  |  | |  | | |  |  | |  | |  |  | | |  |  | |  |  | |  |  |  |  |  |  |  |  |
| HIS | ***Hue*** | | ***Across TD*** |  | | |  |  | |  | 77.66 | | | ± | 3.22 | | a | | 70.51 | | ± | | 2.73 | a | | 75.65 | | ± | 3.22 | | | a | 73.93 | | ± | 3.73 | | a | | | 70.98 | ± | | 4.01 | | a | 67.59 | | | ± | 3.01 | | a |  | |  |  |  |  |  |  |  |  |
|  |  |  | ***0 N*** | 60.36 | | | ± | 1.63 | | c | 66.33 | | | ± | 4.75 | | a | | 60.93 | | ± | | 3.84 | ab | | 64.01 | | ± | 4.07 | | | abcd | 59.59 | | ± | 4.27 | | bcde | | | 56.85 | ± | | 3.43 | | defg | 54.44 | | | ± | 2.68 | | efg |  | |  |  |  |  |  |  |  |  |
|  |  |  | ***30 N*** | 74.04 | | | ± | 1.87 | | b | 78.89 | | | ± | 4.46 | | a | | 68.70 | | ± | | 2.09 | abc | | 79.08 | | ± | 4.57 | | | abcd | 77.13 | | ± | 5.81 | | cdef | | | 71.27 | ± | | 6.07 | | efg | 69.19 | | | ± | 2.50 | | fg |  | |  |  |  |  |  |  |  |  |
|  |  |  | ***90 N*** | 83.76 | | | ± | 1.32 | | a | 87.76 | | | ± | 2.90 | | a | | 81.90 | | ± | | 1.72 | abcd | | 83.87 | | ± | 4.18 | | | abcd | 85.07 | | ± | 2.98 | | def | | | 84.83 | ± | | 4.77 | | efg | 79.15 | | | ± | 2.07 | | g |  | |  |  |  |  |  |  |  |  |
|  |  | |  |  | | |  |  | |  |  | | |  |  | |  | |  | |  | |  |  | |  | |  |  | | |  |  | |  |  | |  | | |  |  | |  | |  |  | | |  |  | |  |  | |  |  |  |  |  |  |  |  |
|  | ***Intensity*** | | ***Across TD*** |  | | |  |  | |  | 0.31 | | | ± | 0.00 | | a | | 0.31 | | ± | | 0.00 | a | | 0.31 | | ± | 0.00 | | | a | 0.31 | | ± | 0.00 | | a | | | 0.32 | ± | | 0.00 | | a | 0.32 | | | ± | 0.00 | | a |  | |  |  |  |  |  |  |  |  |
|  |  |  | ***0 N*** | 0.32 | | | ± | 0.00 | | a | 0.30 | | | ± | 0.01 | | a | | 0.32 | | ± | | 0.01 | a | | 0.31 | | ± | 0.01 | | | a | 0.32 | | ± | 0.00 | | a | | | 0.31 | ± | | 0.01 | | a | 0.33 | | | ± | 0.01 | | a |  | |  |  |  |  |  |  |  |  |
|  |  |  | ***30 N*** | 0.32 | | | ± | 0.00 | | a | 0.32 | | | ± | 0.00 | | a | | 0.32 | | ± | | 0.01 | a | | 0.32 | | ± | 0.00 | | | a | 0.31 | | ± | 0.00 | | a | | | 0.32 | ± | | 0.01 | | a | 0.32 | | | ± | 0.01 | | a |  | |  |  |  |  |  |  |  |  |
|  |  |  | ***90 N*** | 0.31 | | | ± | 0.00 | | a | 0.31 | | | ± | 0.00 | | a | | 0.31 | | ± | | 0.00 | a | | 0.31 | | ± | 0.01 | | | a | 0.31 | | ± | 0.01 | | a | | | 0.33 | ± | | 0.01 | | a | 0.31 | | | ± | 0.01 | | a |  | |  |  |  |  |  |  |  |  |
|  |  | |  |  | | |  |  | |  |  | | |  |  | |  | |  | |  | |  |  | |  | |  |  | | |  |  | |  |  | |  | | |  |  | |  | |  |  | | |  |  | |  |  | |  |  |  |  |  |  |  |  |
|  | ***Saturation*** | | ***Across TD*** |  | | |  |  | |  | 0.32 | | | ± | 0.02 | | a | | 0.32 | | ± | | 0.02 | a | | 0.32 | | ± | 0.02 | | | a | 0.34 | | ± | 0.02 | | a | | | 0.35 | ± | | 0.02 | | a | 0.37 | | | ± | 0.01 | | a |  | |  |  |  |  |  |  |  |  |
|  |  |  | ***0 N*** | 0.39 | | | ± | 0.01 | | a | 0.37 | | | ± | 0.02 | | abc | | 0.37 | | ± | | 0.02 | abc | | 0.39 | | ± | 0.01 | | | ab | 0.40 | | ± | 0.01 | | ab | | | 0.39 | ± | | 0.01 | | ab | 0.40 | | | ± | 0.02 | | a |  | |  |  |  |  |  |  |  |  |
|  |  |  | ***30 N*** | 0.34 | | | ± | 0.01 | | b | 0.33 | | | ± | 0.02 | | bcdefg | | 0.34 | | ± | | 0.01 | abcde | | 0.31 | | ± | 0.03 | | | cdefg | 0.33 | | ± | 0.03 | | abcdef | | | 0.36 | ± | | 0.04 | | abcd | 0.38 | | | ± | 0.02 | | ab |  | |  |  |  |  |  |  |  |  |
|  |  |  | ***90 N*** | 0.28 | | | ± | 0.01 | | c | 0.26 | | | ± | 0.02 | | g | | 0.26 | | ± | | 0.01 | g | | 0.26 | | ± | 0.03 | | | fg | 0.30 | | ± | 0.02 | | defg | | | 0.28 | ± | | 0.02 | | efg | 0.34 | | | ± | 0.01 | | abcdef | | |  |  |  |  |  |  |  |  |
|  |  | |  |  | | |  |  | |  |  | | |  |  | |  | |  | |  | |  |  | |  | |  |  | | |  |  | |  |  | |  | | |  |  | |  | |  |  | | |  |  | |  |  | |  |  |  |  |  |  |  |  |
|  | ***GA*** | | ***Across TD*** |  | | |  |  | |  | 0.59 | | | ± | 0.03 | | a | | 0.55 | | ± | | 0.03 | a | | 0.56 | | ± | 0.03 | | | a | 0.55 | | ± | 0.04 | | a | | | 0.55 | ± | | 0.04 | | a | 0.52 | | | ± | 0.04 | | a |  | |  |  |  |  |  |  |  |  |
|  |  |  | ***0 N*** | 0.42 | | | ± | 0.02 | | c | 0.47 | | | ± | 0.05 | | ghi | | 0.46 | | ± | | 0.03 | hi | | 0.45 | | ± | 0.03 | | | hi | 0.40 | | ± | 0.04 | | i | | | 0.37 | ± | | 0.04 | | i | 0.37 | | | ± | 0.04 | | i |  | |  |  |  |  |  |  |  |  |
|  |  |  | ***30 N*** | 0.57 | | | ± | 0.01 | | b | 0.60 | | | ± | 0.03 | | abcdef | | 0.53 | | ± | | 0.02 | efgh | | 0.59 | | ± | 0.03 | | | bcdef | 0.58 | | ± | 0.05 | | cdefg | | | 0.56 | ± | | 0.05 | | defgh | 0.52 | | | ± | 0.04 | | fgh |  | |  |  |  |  |  |  |  |  |
|  |  |  | ***90 N*** | 0.68 | | | ± | 0.01 | | a | 0.70 | | | ± | 0.03 | | ab | | 0.66 | | ± | | 0.01 | abcd | | 0.65 | | ± | 0.04 | | | abcde | 0.68 | | ± | 0.04 | | abc | | | 0.71 | ± | | 0.03 | | a | 0.66 | | | ± | 0.01 | | abcd |  | |  |  |  |  |  |  |  |  |
|  |  | |  |  | | |  |  | |  |  | | |  |  | |  | |  | |  | |  |  | |  | |  |  | | |  |  | |  |  | |  | | |  |  | |  | |  |  | | |  |  | |  |  | |  |  |  |  |  |  |  |  |
|  | ***GGA*** | | ***Across TD*** |  | | |  |  | |  | 0.48 | | | ± | 0.04 | | a | | 0.41 | | ± | | 0.04 | a | | 0.44 | | ± | 0.04 | | | a | 0.43 | | ± | 0.05 | | a | | | 0.40 | ± | | 0.05 | | a | 0.36 | | | ± | 0.05 | | a |  | |  |  |  |  |  |  |  |  |
|  |  |  | ***0 N*** | 0.25 | | | ± | 0.02 | | c | 0.33 | | | ± | 0.06 | | def | | 0.29 | | ± | | 0.06 | efg | | 0.30 | | ± | 0.04 | | | efg | 0.23 | | ± | 0.05 | | fg | | | 0.20 | ± | | 0.06 | | fg | 0.17 | | | ± | 0.04 | | g |  | |  |  |  |  |  |  |  |  |
|  |  |  | ***30 N*** | 0.44 | | | ± | 0.02 | | b | 0.50 | | | ± | 0.04 | | abc | | 0.39 | | ± | | 0.03 | bcde | | 0.49 | | ± | 0.04 | | | abc | 0.47 | | ± | 0.06 | | abcd | | | 0.40 | ± | | 0.08 | | bcde | 0.38 | | | ± | 0.05 | | cde |  | |  |  |  |  |  |  |  |  |
|  |  |  | ***90 N*** | 0.57 | | | ± | 0.01 | | a | 0.60 | | | ± | 0.03 | | a | | 0.55 | | ± | | 0.01 | ab | | 0.54 | | ± | 0.04 | | | ab | 0.59 | | ± | 0.04 | | a | | | 0.60 | ± | | 0.04 | | a | 0.53 | | | ± | 0.02 | | abc |  | |  |  |  |  |  |  |  |  |
|  |  | |  |  | | |  |  | |  |  | | |  |  | |  | |  | |  | |  |  | |  | |  |  | | |  |  | |  |  | |  | | |  |  | |  | |  |  | | |  |  | |  |  | |  |  |  |  |  |  |  |  |
|  | ***CSI*** | | ***Across TD*** |  | | |  |  | |  | 21.53 | | | ± | 3.62 | | a | | 27.68 | | ± | | 4.30 | a | | 23.18 | | ± | 3.13 | | | a | 26.36 | | ± | 4.55 | | a | | | 32.39 | ± | | 5.58 | | a | 35.41 | | | ± | 5.58 | | a |  | |  |  |  |  |  |  |  |  |
|  |  |  | ***0 N*** | 43.68 | | | ± | 3.65 | | a | 33.42 | | | ± | 8.75 | | bcdef | | 38.67 | | ± | | 10.21 | abcd | | 34.95 | | ± | 5.99 | | | bcde | 45.56 | | ± | 7.50 | | abc | | | 50.80 | ± | | 9.81 | | ab | 58.65 | | | ± | 9.37 | | a |  | |  |  |  |  |  |  |  |  |
|  |  |  | ***30 N*** | 23.42 | | | ± | 2.07 | | b | 17.11 | | | ± | 2.42 | | ef | | 26.76 | | ± | | 5.75 | cdef | | 18.11 | | ± | 3.39 | | | def | 19.90 | | ± | 3.84 | | def | | | 30.88 | ± | | 7.93 | | bcdef | 27.76 | | | ± | 4.01 | | cdef |  | |  |  |  |  |  |  |  |  |
|  |  |  | ***90 N*** | 16.18 | | | ± | 0.94 | | c | 14.07 | | | ± | 1.09 | | ef | | 17.60 | | ± | | 0.88 | def | | 16.47 | | ± | 1.73 | | | ef | 13.61 | | ± | 1.85 | | f | | | 15.49 | ± | | 3.31 | | ef | 19.81 | | | ± | 3.53 | | def |  | |  |  |  |  |  |  |  |  |
|  |  | |  |  | | |  |  | |  |  | | |  |  | |  | |  | |  | |  |  | |  | |  |  | | |  |  | |  |  | |  | | |  |  | |  | |  |  | | |  |  | |  |  | |  |  |  |  |  |  |  |  |
| CIE | ***Lightness*** | | ***Across TD*** |  | | |  |  | |  | 39.71 | | | ± | 0.38 | | a | | 39.57 | | ± | | 0.45 | a | | 39.57 | | ± | 0.36 | | | a | 40.11 | | ± | 0.38 | | a | | | 40.64 | ± | | 0.45 | | a | 40.61 | | | ± | 0.45 | | a |  | |  |  |  |  |  |  |  |  |
|  |  |  | ***0 N*** | 39.87 | | | ± | 0.31 | | a | 38.79 | | | ± | 0.68 | | a | | 39.85 | | ± | | 1.10 | a | | 39.56 | | ± | 0.53 | | | a | 40.10 | | ± | 0.17 | | a | | | 39.50 | ± | | 0.69 | | a | 41.41 | | | ± | 0.91 | | a |  | |  |  |  |  |  |  |  |  |
|  |  |  | ***30 N*** | 40.45 | | | ± | 0.27 | | a | 40.90 | | | ± | 0.58 | | a | | 40.21 | | ± | | 0.59 | a | | 40.12 | | ± | 0.67 | | | a | 39.77 | | ± | 0.60 | | a | | | 41.07 | ± | | 0.80 | | a | 40.62 | | | ± | 0.88 | | a |  | |  |  |  |  |  |  |  |  |
|  |  |  | ***90 N*** | 39.79 | | | ± | 0.30 | | a | 39.44 | | | ± | 0.31 | | a | | 38.64 | | ± | | 0.46 | a | | 39.04 | | ± | 0.67 | | | a | 40.46 | | ± | 1.04 | | a | | | 41.34 | ± | | 0.71 | | a | 39.81 | | | ± | 0.49 | | a |  | |  |  |  |  |  |  |  |  |
|  |  | |  |  | | |  |  | |  |  | | |  |  | |  | |  | |  | |  |  | |  | |  |  | | |  |  | |  |  | |  | | |  |  | |  | |  |  | | |  |  | |  |  | |  |  |  |  |  |  |  |  |
|  | ***a**** | | ***Across TD*** |  | | |  |  | |  | -13.49 | | | ± | 0.94 | | a | | -10.93 | | ± | | 0.74 | a | | -12.59 | | ± | 0.80 | | | a | -12.66 | | ± | 1.12 | | a | | | -11.57 | ± | | 1.20 | | a | -10.96 | | | ± | 1.14 | | a |  | |  |  |  |  |  |  |  |  |
|  |  |  | ***0 N*** | -8.17 | | | ± | 0.62 | | a | -10.09 | | | ± | 1.77 | | bcdef | | -8.29 | | ± | | 1.42 | abcd | | -9.67 | | ± | 1.43 | | | abcde | -8.15 | | ± | 1.64 | | abc | | | -6.87 | ± | | 1.37 | | ab | -5.95 | | | ± | 1.40 | | a |  | |  |  |  |  |  |  |  |  |
|  |  |  | ***30 N*** | -12.89 | | | ± | 0.46 | | b | -14.72 | | | ± | 1.22 | | ghij | | -11.09 | | ± | | 0.55 | cdefg | | -13.98 | | ± | 1.06 | | | ghij | -13.54 | | ± | 1.08 | | fghij | | | -12.08 | ± | | 1.42 | | efghi | -11.93 | | | ± | 0.72 | | defgh |  | |  |  |  |  |  |  |  |  |
|  |  |  | ***90 N*** | -15.03 | | | ± | 0.33 | | c | -15.66 | | | ± | 0.49 | | hij | | -13.41 | | ± | | 0.30 | efghij | | -14.11 | | ± | 0.65 | | | ghij | -16.28 | | ± | 0.83 | | j | | | -15.76 | ± | | 1.12 | | ij | -14.98 | | | ± | 0.73 | | hij |  | |  |  |  |  |  |  |  |  |
|  |  | |  |  | | |  |  | |  |  | | |  |  | |  | |  | |  | |  |  | |  | |  |  | | |  |  | |  |  | |  | | |  |  | |  | |  |  | | |  |  | |  |  | |  |  |  |  |  |  |  |  |
|  | ***b**** | | ***Across TD*** |  | | |  |  | |  | 27.84 | | | ± | 0.79 | | a | | 27.74 | | ± | | 0.88 | a | | 27.67 | | ± | 0.94 | | | a | 29.20 | | ± | 0.78 | | a | | | 29.47 | ± | | 0.90 | | a | 30.87 | | | ± | 0.62 | | a |  | |  |  |  |  |  |  |  |  |
|  |  |  | ***0 N*** | 30.84 | | | ± | 0.40 | | a | 29.59 | | | ± | 1.23 | | abc | | 30.04 | | ± | | 1.34 | abc | | 30.76 | | ± | 0.80 | | | abc | 31.51 | | ± | 0.48 | | ab | | | 30.73 | ± | | 0.70 | | abc | 32.42 | | | ± | 1.04 | | a |  | |  |  |  |  |  |  |  |  |
|  |  |  | ***30 N*** | 29.38 | | | ± | 0.60 | | b | 29.02 | | | ± | 1.02 | | abcd | | 29.03 | | ± | | 0.88 | abcd | | 27.55 | | ± | 1.53 | | | bcdef | 28.65 | | ± | 1.80 | | abcde | | | 30.74 | ± | | 2.08 | | abc | 31.28 | | | ± | 1.14 | | ab |  | |  |  |  |  |  |  |  |  |
|  |  |  | ***90 N*** | 26.18 | | | ± | 0.47 | | c | 24.93 | | | ± | 0.87 | | def | | 24.15 | | ± | | 0.80 | f | | 24.70 | | ± | 1.33 | | | ef | 27.44 | | ± | 0.88 | | bcdef | | | 26.94 | ± | | 1.17 | | cdef | 28.91 | | | ± | 0.32 | | abcde | | |  |  |  |  |  |  |  |  |
|  |  | |  |  | | |  |  | |  |  | | |  |  | |  | |  | |  | |  |  | |  | |  |  | | |  |  | |  |  | |  | | |  |  | |  | |  |  | | |  |  | |  |  | |  |  |  |  |  |  |  |  |
|  | ***u**** | | ***Across TD*** |  | | |  |  | |  | -5.99 | | | ± | 1.41 | | a | | -2.68 | | ± | | 1.21 | a | | -4.87 | | ± | 1.28 | | | a | -4.48 | | ± | 1.65 | | a | | | -2.94 | ± | | 1.78 | | a | -1.69 | | | ± | 1.71 | | a |  | |  |  |  |  |  |  |  |  |
|  |  |  | ***0 N*** | 1.95 | | | ± | 0.94 | | a | -1.06 | | | ± | 2.62 | | bcde | | 1.54 | | ± | | 2.22 | abc | | -0.13 | | ± | 2.13 | | | abcd | 2.15 | | ± | 2.30 | | abc | | | 3.62 | ± | | 2.02 | | ab | 5.60 | | | ± | 2.37 | | a |  | |  |  |  |  |  |  |  |  |
|  |  |  | ***30 N*** | -4.77 | | | ± | 0.73 | | b | -7.27 | | | ± | 1.85 | | fg | | -2.53 | | ± | | 0.97 | cdef | | -6.71 | | ± | 1.72 | | | efg | -5.84 | | ± | 1.85 | | defg | | | -3.25 | ± | | 2.42 | | cdef | -3.00 | | | ± | 1.15 | | cdef |  | |  |  |  |  |  |  |  |  |
|  |  |  | ***90 N*** | -8.51 | | | ± | 0.45 | | c | -9.65 | | | ± | 0.78 | | g | | -7.04 | | ± | | 0.45 | fg | | -7.75 | | ± | 1.13 | | | fg | -9.74 | | ± | 1.16 | | g | | | -9.20 | ± | | 1.69 | | g | -7.66 | | | ± | 0.98 | | fg |  | |  |  |  |  |  |  |  |  |
|  |  | |  |  | | |  |  | |  |  | | |  |  | |  | |  | |  | |  |  | |  | |  |  | | |  |  | |  |  | |  | | |  |  | |  | |  |  | | |  |  | |  |  | |  |  |  |  |  |  |  |  |
|  | ***v**** | | ***Across TD*** |  | | |  |  | |  | 30.06 | | | ± | 0.57 | | ab | | 29.48 | | ± | | 0.70 | b | | 29.70 | | ± | 0.69 | | | b | 31.07 | | ± | 0.56 | | ab | | | 31.22 | ± | | 0.64 | | ab | 32.19 | | | ± | 0.43 | | a |  | |  |  |  |  |  |  |  |  |
|  |  |  | ***0 N*** | 31.39 | | | ± | 0.32 | | a | 30.46 | | | ± | 0.95 | | abc | | 30.84 | | ± | | 1.23 | abc | | 31.51 | | ± | 0.57 | | | ab | 31.96 | | ± | 0.33 | | a | | | 30.95 | ± | | 0.64 | | abc | 32.64 | | | ± | 0.72 | | a |  | |  |  |  |  |  |  |  |  |
|  |  |  | ***30 N*** | 31.38 | | | ± | 0.47 | | a | 31.61 | | | ± | 0.62 | | ab | | 30.76 | | ± | | 0.73 | abc | | 30.09 | | ± | 1.24 | | | abcd | 30.70 | | ± | 1.43 | | abc | | | 32.41 | ± | | 1.60 | | a | 32.70 | | | ± | 1.03 | | a |  | |  |  |  |  |  |  |  |  |
|  |  |  | ***90 N*** | 29.08 | | | ± | 0.42 | | b | 28.11 | | | ± | 0.75 | | bcd | | 26.85 | | ± | | 0.71 | d | | 27.48 | | ± | 1.04 | | | cd | 30.54 | | ± | 0.89 | | abc | | | 30.29 | ± | | 0.85 | | abcd | 31.23 | | | ± | 0.13 | | ab |  | |  |  |  |  |  |  |  |  |
|  |  | |  |  | | |  |  | |  |  | | |  |  | |  | |  | |  | |  |  | |  | |  |  | | |  |  | |  |  | |  | | |  |  | |  | |  |  | | |  |  | |  |  | |  |  |  |  |  |  |  |  |
| RGB | ***NGRDI*** | | ***Across TD*** |  | | |  |  | |  | 0.02 | | | ± | 0.02 | | a | | -0.01 | | ± | | 0.01 | a | | 0.01 | | ± | 0.01 | | | a | 0.01 | | ± | 0.02 | | a | | | 0.00 | ± | | 0.02 | | a | -0.01 | | | ± | 0.02 | | a |  | |  |  |  |  |  |  |  |  |
|  |  |  | ***0 N*** | -0.06 | | | ± | 0.01 | | c | -0.04 | | | ± | 0.02 | | fghi | | -0.06 | | ± | | 0.02 | ghi | | -0.05 | | ± | 0.02 | | | fghi | -0.07 | | ± | 0.02 | | hi | | | -0.07 | ± | | 0.02 | | hi | -0.09 | | | ± | 0.02 | | i |  | |  |  |  |  |  |  |  |  |
|  |  |  | ***30 N*** | 0.01 | | | ± | 0.01 | | b | 0.03 | | | ± | 0.02 | | abcde | | -0.02 | | ± | | 0.01 | efgh | | 0.03 | | ± | 0.02 | | | abcde | 0.02 | | ± | 0.03 | | bcde | | | 0.01 | ± | | 0.03 | | cdef | -0.01 | | | ± | 0.02 | | defg |  | |  |  |  |  |  |  |  |  |
|  |  |  | ***90 N*** | 0.06 | | | ± | 0.01 | | a | 0.07 | | | ± | 0.01 | | ab | | 0.05 | | ± | | 0.01 | abcd | | 0.05 | | ± | 0.02 | | | abcd | 0.07 | | ± | 0.02 | | ab | | | 0.08 | ± | | 0.02 | | a | 0.06 | | | ± | 0.01 | | abc |  | |  |  |  |  |  |  |  |  |
|  |  | |  |  | | |  |  | |  |  | | |  |  | |  | |  | |  | |  |  | |  | |  |  | | |  |  | |  |  | |  | | |  |  | |  | |  |  | | |  |  | |  |  | |  |  |  |  |  |  |  |  |
|  | ***TGI*** | | ***Across TD*** |  | | |  |  | |  | 3076.92 | | | ± | 71.01 | | a | | 2788.56 | | ± | | 57.63 | b | | 2960.75 | | ± | 59.65 | | | a | 3115.45 | | ± | 81.70 | | a | | | 3022.78 | ± | | 95.04 | | ab | 3092.48 | | | ± | 86.35 | | b |  | |  |  |  |  |  |  |  |  |
|  |  |  | ***0 N*** | 2790.62 | | | ± | 50.87 | | b | 2885.45 | | | ± | 146.16 | | cdef | | 2724.97 | | ± | | 134.66 | ef | | 2944.00 | | ± | 100.97 | | | bcdef | 2850.33 | | ± | 148.96 | | def | | | 2641.46 | ± | | 132.27 | | f | 2697.49 | | | ± | 73.11 | | ef |  | |  |  |  |  |  |  |  |  |
|  |  |  | ***30 N*** | 3158.17 | | | ± | 40.01 | | a | 3317.29 | | | ± | 50.35 | | a | | 2922.25 | | ± | | 57.54 | bcdef | | 3097.13 | | ± | 102.58 | | | abcd | 3162.95 | | ± | 88.41 | | abcd | | | 3203.77 | ± | | 117.08 | | abc | 3245.60 | | | ± | 90.27 | | ab |  | |  |  |  |  |  |  |  |  |
|  |  |  | ***90 N*** | 3079.69 | | | ± | 54.53 | | a | 3028.03 | | | ± | 69.59 | | abcde | | 2718.47 | | ± | | 81.97 | ef | | 2841.11 | | ± | 91.33 | | | def | 3333.08 | | ± | 100.56 | | a | | | 3223.11 | ± | | 95.25 | | abc | 3334.35 | | | ± | 72.89 | | a |  | |  |  |  |  |  |  |  |  |
|  |  | |  |  | | |  |  | |  |  | | |  |  | |  | |  | |  | |  |  | |  | |  |  | | |  |  | |  |  | |  | | |  |  | |  | |  |  | | |  |  | |  |  | |  |  |  |  |  |  |  |  |
|  |  | |  |  | | |  |  | |  | ***RGB aerial*** | | | | | | | | | | | | | | | | | | | | | | | | | | | | | | | | | | | | | | | | | | |  | |  |  |  |  |  |  |  |  |
| ***Index*** | | | **Tillage** | ***Across TD*** | | | | | | | ***Conventional Tillage*** | | | | | | | | ***No-tillage*** | | | | | | | | | | | | | | | | | | | | | | | | | | | | | | | | | | |  | |  |  |  |  |  |  |  |  |
|  |  |  | ***Residue level*** |  |  |  |  |  |  |  | ***4 Mg/ha*** | | | | | | | | ***0 Mg/ha*** | | | | | | | ***2 Mg/ha*** | | | | | | | ***4 Mg/ha*** | | | | | | | | ***6 Mg/ha*** | | | | | | ***8 Mg/ha*** | | | | | | |  | |  |  |  |  |  |  |  |  |
|  |  | |  |  | | |  |  | |  |  | | |  |  | |  | |  | |  | |  |  | |  | |  |  | | |  |  | |  |  | |  | | |  |  | |  | |  |  | | |  |  | |  |  | |  |  |  |  |  |  |  |  |
| HIS | ***Hue*** | | ***Across TD*** |  | | |  |  | |  | 64.00 | | | ± | 1.77 | | a | | 62.03 | | ± | | 1.66 | a | | 61.81 | | ± | 1.66 | | | a | 64.39 | | ± | 2.43 | | a | | | 64.94 | ± | | 2.57 | | a | 63.42 | | | ± | 2.04 | | a |  | |  |  |  |  |  |  |  |  |
|  |  |  | ***0 N*** | 56.98 | | | ± | 1.03 | | c | 59.17 | | | ± | 2.47 | | fg | | 60.13 | | ± | | 3.89 | fg | | 55.83 | | ± | 2.51 | | | g | 55.36 | | ± | 2.97 | | g | | | 55.66 | ± | | 1.54 | | g | 55.72 | | | ± | 1.58 | | g |  | |  |  |  |  |  |  |  |  |
|  |  |  | ***30 N*** | 63.16 | | | ± | 0.71 | | b | 63.45 | | | ± | 2.55 | | def | | 61.07 | | ± | | 2.21 | efg | | 62.67 | | ± | 1.34 | | | ef | 65.14 | | ± | 2.15 | | cdef | | | 63.63 | ± | | 0.98 | | def | 62.98 | | | ± | 1.17 | | def |  | |  |  |  |  |  |  |  |  |
|  |  |  | ***90 N*** | 70.51 | | | ± | 0.85 | | a | 69.38 | | | ± | 2.12 | | abcd | | 65.84 | | ± | | 0.52 | bcde | | 66.92 | | ± | 1.09 | | | bcde | 72.66 | | ± | 1.04 | | ab | | | 75.53 | ± | | 1.64 | | a | 71.55 | | | ± | 0.39 | | abc |  | |  |  |  |  |  |  |  |  |
|  |  | |  |  | | |  |  | |  |  | | |  |  | |  | |  | |  | |  |  | |  | |  |  | | |  |  | |  |  | |  | | |  |  | |  | |  |  | | |  |  | |  |  | |  |  |  |  |  |  |  |  |
|  | ***Intensity*** | | ***Across TD*** |  | | |  |  | |  | 0.46 | | | ± | 0.00 | | a | | 0.46 | | ± | | 0.00 | a | | 0.46 | | ± | 0.01 | | | a | 0.47 | | ± | 0.01 | | a | | | 0.47 | ± | | 0.01 | | a | 0.48 | | | ± | 0.01 | | a |  | |  |  |  |  |  |  |  |  |
|  |  |  | ***0 N*** | 0.48 | | | ± | 0.00 | | a | 0.46 | | | ± | 0.01 | | efg | | 0.47 | | ± | | 0.00 | cdef | | 0.48 | | ± | 0.01 | | | abc | 0.49 | | ± | 0.01 | | ab | | | 0.50 | ± | | 0.01 | | a | 0.50 | | | ± | 0.00 | | ab |  | |  |  |  |  |  |  |  |  |
|  |  |  | ***30 N*** | 0.47 | | | ± | 0.00 | | b | 0.47 | | | ± | 0.01 | | cdef | | 0.47 | | ± | | 0.01 | defg | | 0.46 | | ± | 0.00 | | | efg | 0.47 | | ± | 0.01 | | cdef | | | 0.48 | ± | | 0.00 | | bcde | 0.48 | | | ± | 0.01 | | abcd |  | |  |  |  |  |  |  |  |  |
|  |  |  | ***90 N*** | 0.44 | | | ± | 0.00 | | c | 0.44 | | | ± | 0.00 | | h | | 0.44 | | ± | | 0.00 | h | | 0.45 | | ± | 0.00 | | | gh | 0.44 | | ± | 0.01 | | h | | | 0.44 | ± | | 0.00 | | h | 0.46 | | | ± | 0.01 | | fgh |  | |  |  |  |  |  |  |  |  |
|  |  | |  |  | | |  |  | |  |  | | |  |  | |  | |  | |  | |  |  | |  | |  |  | | |  |  | |  |  | |  | | |  |  | |  | |  |  | | |  |  | |  |  | |  |  |  |  |  |  |  |  |
|  | ***Saturation*** | | ***Across TD*** |  | | |  |  | |  | 0.25 | | | ± | 0.01 | | a | | 0.25 | | ± | | 0.01 | a | | 0.25 | | ± | 0.01 | | | a | 0.26 | | ± | 0.01 | | a | | | 0.26 | ± | | 0.01 | | a | 0.27 | | | ± | 0.01 | | a |  | |  |  |  |  |  |  |  |  |
|  |  |  | ***0 N*** | 0.29 | | | ± | 0.00 | | a | 0.28 | | | ± | 0.01 | | abcde | | 0.27 | | ± | | 0.00 | bcde | | 0.29 | | ± | 0.01 | | | abc | 0.30 | | ± | 0.00 | | a | | | 0.29 | ± | | 0.01 | | ab | 0.30 | | | ± | 0.01 | | ab |  | |  |  |  |  |  |  |  |  |
|  |  |  | ***30 N*** | 0.27 | | | ± | 0.00 | | b | 0.26 | | | ± | 0.01 | | de | | 0.26 | | ± | | 0.00 | de | | 0.26 | | ± | 0.01 | | | e | 0.26 | | ± | 0.01 | | cde | | | 0.28 | ± | | 0.01 | | bcde | 0.28 | | | ± | 0.01 | | abcd |  | |  |  |  |  |  |  |  |  |
|  |  |  | ***90 N*** | 0.22 | | | ± | 0.00 | | c | 0.22 | | | ± | 0.01 | | f | | 0.22 | | ± | | 0.00 | f | | 0.22 | | ± | 0.01 | | | f | 0.22 | | ± | 0.00 | | f | | | 0.21 | ± | | 0.01 | | f | 0.23 | | | ± | 0.00 | | f |  | |  |  |  |  |  |  |  |  |
|  |  | |  |  | | |  |  | |  |  | | |  |  | |  | |  | |  | |  |  | |  | |  |  | | |  |  | |  |  | |  | | |  |  | |  | |  |  | | |  |  | |  |  | |  |  |  |  |  |  |  |  |
|  | ***GA*** | | ***Across TD*** |  | | |  |  | |  | 0.63 | | | ± | 0.03 | | a | | 0.60 | | ± | | 0.03 | a | | 0.59 | | ± | 0.03 | | | a | 0.62 | | ± | 0.04 | | a | | | 0.62 | ± | | 0.05 | | a | 0.59 | | | ± | 0.04 | | a |  | |  |  |  |  |  |  |  |  |
|  |  |  | ***0 N*** | 0.49 | | | ± | 0.02 | | c | 0.54 | | | ± | 0.04 | | efg | | 0.56 | | ± | | 0.06 | ef | | 0.47 | | ± | 0.04 | | | fg | 0.46 | | ± | 0.05 | | g | | | 0.45 | ± | | 0.03 | | g | 0.45 | | | ± | 0.03 | | g |  | |  |  |  |  |  |  |  |  |
|  |  |  | ***30 N*** | 0.60 | | | ± | 0.01 | | b | 0.62 | | | ± | 0.04 | | de | | 0.58 | | ± | | 0.03 | e | | 0.60 | | ± | 0.03 | | | de | 0.64 | | ± | 0.03 | | cde | | | 0.61 | ± | | 0.01 | | de | 0.58 | | | ± | 0.02 | | e |  | |  |  |  |  |  |  |  |  |
|  |  |  | ***90 N*** | 0.74 | | | ± | 0.01 | | a | 0.73 | | | ± | 0.04 | | abc | | 0.67 | | ± | | 0.01 | bcd | | 0.68 | | ± | 0.01 | | | bcd | 0.76 | | ± | 0.02 | | ab | | | 0.81 | ± | | 0.02 | | a | 0.74 | | | ± | 0.01 | | abc |  | |  |  |  |  |  |  |  |  |
|  |  | |  |  | | |  |  | |  |  | | |  |  | |  | |  | |  | |  |  | |  | |  |  | | |  |  | |  |  | |  | | |  |  | |  | |  |  | | |  |  | |  |  | |  |  |  |  |  |  |  |  |
|  | ***GGA*** | | ***Across TD*** |  | | |  |  | |  | 0.22 | | | ± | 0.03 | | a | | 0.19 | | ± | | 0.02 | a | | 0.19 | | ± | 0.03 | | | a | 0.22 | | ± | 0.04 | | a | | | 0.23 | ± | | 0.05 | | a | 0.19 | | | ± | 0.04 | | a |  | |  |  |  |  |  |  |  |  |
|  |  |  | ***0 N*** | 0.10 | | | ± | 0.01 | | c | 0.15 | | | ± | 0.04 | | ghijk | | 0.16 | | ± | | 0.04 | ghijk | | 0.09 | | ± | 0.03 | | | hijk | 0.06 | | ± | 0.02 | | k | | | 0.07 | ± | | 0.02 | | ijk | 0.07 | | | ± | 0.02 | | jk |  | |  |  |  |  |  |  |  |  |
|  |  |  | ***30 N*** | 0.18 | | | ± | 0.01 | | b | 0.20 | | | ± | 0.04 | | efg | | 0.17 | | ± | | 0.03 | ghi | | 0.19 | | ± | 0.03 | | | fgh | 0.21 | | ± | 0.03 | | defg | | | 0.17 | ± | | 0.02 | | ghij | 0.16 | | | ± | 0.02 | | ghijk |  | |  |  |  |  |  |  |  |  |
|  |  |  | ***90 N*** | 0.34 | | | ± | 0.02 | | a | 0.31 | | | ± | 0.04 | | bcd | | 0.26 | | ± | | 0.01 | bcde | | 0.28 | | ± | 0.02 | | | cdef | 0.38 | | ± | 0.02 | | ab | | | 0.44 | ± | | 0.05 | | a | 0.34 | | | ± | 0.01 | | bc |  | |  |  |  |  |  |  |  |  |
|  |  | |  |  | | |  |  | |  |  | | |  |  | |  | |  | |  | |  |  | |  | |  |  | | |  |  | |  |  | |  | | |  |  | |  | |  |  | | |  |  | |  |  | |  |  |  |  |  |  |  |  |
|  | ***CSI*** | | ***Across TD*** |  | | |  |  | |  | 67.20 | | | ± | 3.23 | | a | | 69.19 | | ± | | 2.39 | a | | 70.14 | | ± | 3.53 | | | a | 67.91 | | ± | 4.91 | | a | | | 67.96 | ± | | 5.23 | | a | 70.94 | | | ± | 4.08 | | a |  | |  |  |  |  |  |  |  |  |
|  |  |  | ***0 N*** | 81.01 | | | ± | 1.96 | | a | 73.98 | | | ± | 5.37 | | bcd | | 72.73 | | ± | | 4.39 | cd | | 82.09 | | ± | 4.89 | | | abc | 87.09 | | ± | 3.94 | | a | | | 85.07 | ± | | 4.01 | | ab | 85.08 | | | ± | 3.28 | | ab |  | |  |  |  |  |  |  |  |  |
|  |  |  | ***30 N*** | 70.51 | | | ± | 1.42 | | b | 69.26 | | | ± | 5.46 | | de | | 71.36 | | ± | | 3.39 | cd | | 69.33 | | ± | 4.36 | | | de | 67.03 | | ± | 3.15 | | de | | | 72.53 | ± | | 2.18 | | cd | 73.57 | | | ± | 2.75 | | bcd |  | |  |  |  |  |  |  |  |  |
|  |  |  | ***90 N*** | 54.54 | | | ± | 1.54 | | c | 58.36 | | | ± | 3.69 | | ef | | 61.59 | | ± | | 1.68 | ef | | 59.01 | | ± | 2.17 | | | ef | 49.59 | | ± | 2.17 | | fg | | | 46.28 | ± | | 4.29 | | g | 54.18 | | | ± | 1.48 | | fg |  | |  |  |  |  |  |  |  |  |
|  |  | |  |  | | |  |  | |  |  | | |  |  | |  | |  | |  | |  |  | |  | |  |  | | |  |  | |  |  | |  | | |  |  | |  | |  |  | | |  |  | |  |  | |  |  |  |  |  |  |  |  |
| CIE | ***Lightness*** | | ***Across TD*** |  | | |  |  | |  | 54.15 | | | ± | 0.59 | | a | | 54.25 | | ± | | 0.53 | a | | 54.91 | | ± | 0.66 | | | a | 55.51 | | ± | 0.85 | | a | | | 56.21 | ± | | 0.89 | | a | 56.83 | | | ± | 0.66 | | a |  | |  |  |  |  |  |  |  |  |
|  |  |  | ***0 N*** | 57.33 | | | ± | 0.41 | | a | 55.15 | | | ± | 0.89 | | ef | | 55.38 | | ± | | 0.20 | def | | 57.28 | | ± | 0.85 | | | abcd | 58.37 | | ± | 0.60 | | ab | | | 59.08 | ± | | 0.93 | | a | 58.70 | | | ± | 0.34 | | ab |  | |  |  |  |  |  |  |  |  |
|  |  |  | ***30 N*** | 55.95 | | | ± | 0.33 | | b | 55.36 | | | ± | 0.71 | | def | | 55.05 | | ± | | 0.46 | f | | 54.79 | | ± | 0.67 | | | f | 55.82 | | ± | 1.05 | | cdef | | | 57.03 | ± | | 0.32 | | bcde | 57.64 | | | ± | 0.65 | | abc |  | |  |  |  |  |  |  |  |  |
|  |  |  | ***90 N*** | 52.59 | | | ± | 0.24 | | c | 51.95 | | | ± | 0.21 | | h | | 51.69 | | ± | | 0.17 | h | | 52.66 | | ± | 0.33 | | | gh | 52.35 | | ± | 0.65 | | gh | | | 52.52 | ± | | 0.54 | | gh | 54.13 | | | ± | 0.66 | | fg |  | |  |  |  |  |  |  |  |  |
|  |  | |  |  | | |  |  | |  |  | | |  |  | |  | |  | |  | |  |  | |  | |  |  | | |  |  | |  |  | |  | | |  |  | |  | |  |  | | |  |  | |  |  | |  |  |  |  |  |  |  |  |
|  | ***a**** | | ***Across TD*** |  | | |  |  | |  | -10.13 | | | ± | 0.45 | | a | | -9.62 | | ± | | 0.54 | a | | -9.51 | | ± | 0.49 | | | a | -10.56 | | ± | 0.72 | | a | | | -10.83 | ± | | 0.62 | | a | -10.62 | | | ± | 0.57 | | a |  | |  |  |  |  |  |  |  |  |
|  |  |  | ***0 N*** | -8.54 | | | ± | 0.39 | | a | -9.05 | | | ± | 0.83 | | abc | | -9.43 | | ± | | 1.48 | abc | | -8.02 | | ± | 0.98 | | | a | -8.10 | | ± | 1.41 | | a | | | -8.29 | ± | | 0.54 | | ab | -8.33 | | | ± | 0.70 | | ab |  | |  |  |  |  |  |  |  |  |
|  |  |  | ***30 N*** | -10.60 | | | ± | 0.24 | | b | -10.39 | | | ± | 0.71 | | abcde | | -9.56 | | ± | | 0.66 | abc | | -10.10 | | ± | 0.62 | | | abcd | -11.31 | | ± | 0.67 | | cde | | | -11.18 | ± | | 0.31 | | cde | -11.06 | | | ± | 0.29 | | cde |  | |  |  |  |  |  |  |  |  |
|  |  |  | ***90 N*** | -11.58 | | | ± | 0.28 | | c | -10.95 | | | ± | 0.61 | | bcde | | -9.95 | | ± | | 0.20 | abcde | | -10.39 | | ± | 0.48 | | | abcde | -12.27 | | ± | 0.40 | | de | | | -13.03 | ± | | 0.22 | | e | -12.49 | | | ± | 0.27 | | de |  | |  |  |  |  |  |  |  |  |
|  |  | |  |  | | |  |  | |  |  | | |  |  | |  | |  | |  | |  |  | |  | |  |  | | |  |  | |  |  | |  | | |  |  | |  | |  |  | | |  |  | |  |  | |  |  |  |  |  |  |  |  |
|  | ***b**** | | ***Across TD*** |  | | |  |  | |  | 30.12 | | | ± | 0.93 | | a | | 30.31 | | ± | | 0.73 | a | | 30.65 | | ± | 1.00 | | | a | 31.66 | | ± | 1.07 | | a | | | 31.96 | ± | | 1.11 | | a | 32.75 | | | ± | 0.91 | | a |  | |  |  |  |  |  |  |  |  |
|  |  |  | ***0 N*** | 34.14 | | | ± | 0.39 | | a | 32.39 | | | ± | 1.23 | | bcd | | 32.28 | | ± | | 0.39 | bcd | | 34.04 | | ± | 0.82 | | | ab | 35.62 | | ± | 0.39 | | a | | | 35.16 | ± | | 0.71 | | a | 35.37 | | | ± | 0.59 | | a |  | |  |  |  |  |  |  |  |  |
|  |  |  | ***30 N*** | 32.12 | | | ± | 0.43 | | b | 31.30 | | | ± | 1.26 | | cd | | 30.96 | | ± | | 0.44 | de | | 30.78 | | ± | 1.32 | | | de | 32.01 | | ± | 0.94 | | bcd | | | 33.57 | ± | | 0.60 | | abc | 34.11 | | | ± | 0.57 | | ab |  | |  |  |  |  |  |  |  |  |
|  |  |  | ***90 N*** | 27.34 | | | ± | 0.27 | | c | 26.66 | | | ± | 0.58 | | f | | 26.82 | | ± | | 0.19 | f | | 27.14 | | ± | 0.74 | | | f | 27.36 | | ± | 0.49 | | f | | | 27.16 | ± | | 0.81 | | f | 28.77 | | | ± | 0.52 | | ef |  | |  |  |  |  |  |  |  |  |
|  |  | |  |  | | |  |  | |  |  | | |  |  | |  | |  | |  | |  |  | |  | |  |  | | |  |  | |  |  | |  | | |  |  | |  | |  |  | | |  |  | |  |  | |  |  |  |  |  |  |  |  |
|  | ***u**** | | ***Across TD*** |  | | |  |  | |  | -0.15 | | | ± | 0.91 | | a | | 0.66 | | ± | | 0.89 | a | | 0.99 | | ± | 0.98 | | | a | -0.10 | | ± | 1.37 | | a | | | -0.35 | ± | | 1.30 | | a | 0.24 | | | ± | 1.14 | | a |  | |  |  |  |  |  |  |  |  |
|  |  |  | ***0 N*** | 3.83 | | | ± | 0.66 | | a | 2.29 | | | ± | 1.52 | | abc | | 1.71 | | ± | | 2.16 | abcd | | 4.55 | | ± | 1.71 | | | ab | 5.07 | | ± | 2.07 | | a | | | 4.67 | ± | | 1.06 | | ab | 4.66 | | | ± | 1.10 | | ab |  | |  |  |  |  |  |  |  |  |
|  |  |  | ***30 N*** | -0.03 | | | ± | 0.37 | | b | -0.05 | | | ± | 1.36 | | cde | | 1.02 | | ± | | 1.12 | bcde | | 0.13 | | ± | 0.72 | | | cde | -1.10 | | ± | 1.06 | | cdefg | | | -0.31 | ± | | 0.55 | | cdef | 0.10 | | | ± | 0.66 | | cde |  | |  |  |  |  |  |  |  |  |
|  |  |  | ***90 N*** | -3.31 | | | ± | 0.37 | | c | -2.68 | | | ± | 0.88 | | efgh | | -1.23 | | ± | | 0.22 | defgh | | -1.71 | | ± | 0.51 | | | defgh | -4.28 | | ± | 0.49 | | gh | | | -5.41 | ± | | 0.52 | | h | -4.02 | | | ± | 0.23 | | fgh |  | |  |  |  |  |  |  |  |  |
|  |  | |  |  | | |  |  | |  |  | | |  |  | |  | |  | |  | |  |  | |  | |  |  | | |  |  | |  |  | |  | | |  |  | |  | |  |  | | |  |  | |  |  | |  |  |  |  |  |  |  |  |
|  | ***v**** | | ***Across TD*** |  | | |  |  | |  | 35.44 | | | ± | 0.93 | | a | | 35.55 | | ± | | 0.76 | a | | 35.97 | | ± | 1.01 | | | a | 37.23 | | ± | 1.07 | | a | | | 37.73 | ± | | 1.10 | | a | 38.55 | | | ± | 0.89 | | a |  | |  |  |  |  |  |  |  |  |
|  |  |  | ***0 N*** | 39.49 | | | ± | 0.42 | | a | 37.50 | | | ± | 1.22 | | cde | | 37.55 | | ± | | 0.46 | cde | | 39.29 | | ± | 0.82 | | | abcd | 40.96 | | ± | 0.55 | | a | | | 40.79 | ± | | 0.73 | | a | 40.88 | | | ± | 0.59 | | a |  | |  |  |  |  |  |  |  |  |
|  |  |  | ***30 N*** | 37.78 | | | ± | 0.47 | | b | 36.86 | | | ± | 1.24 | | def | | 36.32 | | ± | | 0.39 | ef | | 36.19 | | ± | 1.41 | | | ef | 37.80 | | ± | 1.08 | | bcde | | | 39.46 | ± | | 0.58 | | abc | 40.07 | | | ± | 0.58 | | ab |  | |  |  |  |  |  |  |  |  |
|  |  |  | ***90 N*** | 32.84 | | | ± | 0.32 | | c | 31.95 | | | ± | 0.59 | | h | | 31.85 | | ± | | 0.24 | h | | 32.44 | | ± | 0.81 | | | gh | 32.94 | | ± | 0.59 | | gh | | | 32.94 | ± | | 0.84 | | gh | 34.70 | | | ± | 0.64 | | fg |  | |  |  |  |  |  |  |  |  |
|  |  | |  |  | | |  |  | |  |  | | |  |  | |  | |  | |  | |  |  | |  | |  |  | | |  |  | |  |  | |  | | |  |  | |  | |  |  | | |  |  | |  |  | |  |  |  |  |  |  |  |  |
| RGB | ***NGRDI*** | | ***Across TD*** |  | | |  |  | |  | 0.00 | | | ± | 0.01 | | a | | -0.01 | | ± | | 0.01 | a | | -0.01 | | ± | 0.01 | | | a | 0.00 | | ± | 0.01 | | a | | | 0.00 | ± | | 0.01 | | a | 0.00 | | | ± | 0.01 | | a |  | |  |  |  |  |  |  |  |  |
|  |  |  | ***0 N*** | -0.03 | | | ± | 0.00 | | c | -0.02 | | | ± | 0.01 | | fgh | | -0.02 | | ± | | 0.01 | efgh | | -0.03 | | ± | 0.01 | | | h | -0.03 | | ± | 0.01 | | h | | | -0.03 | ± | | 0.01 | | gh | -0.03 | | | ± | 0.01 | | gh |  | |  |  |  |  |  |  |  |  |
|  |  |  | ***30 N*** | 0.00 | | | ± | 0.00 | | b | -0.01 | | | ± | 0.01 | | cdefg | | -0.01 | | ± | | 0.01 | defgh | | -0.01 | | ± | 0.00 | | | cdefg | 0.00 | | ± | 0.01 | | bcde | | | 0.00 | ± | | 0.00 | | cdef | 0.00 | | | ± | 0.00 | | cdef |  | |  |  |  |  |  |  |  |  |
|  |  |  | ***90 N*** | 0.02 | | | ± | 0.00 | | a | 0.01 | | | ± | 0.01 | | abc | | 0.00 | | ± | | 0.00 | bcd | | 0.01 | | ± | 0.00 | | | bcd | 0.03 | | ± | 0.00 | | ab | | | 0.04 | ± | | 0.01 | | a | 0.02 | | | ± | 0.00 | | ab |  | |  |  |  |  |  |  |  |  |
|  |  | |  |  | | |  |  | |  |  | | |  |  | |  | |  | |  | |  |  | |  | |  |  | | |  |  | |  |  | |  | | |  |  | |  | |  |  | | |  |  | |  |  | |  |  |  |  |  |  |  |  |
|  | ***TGI*** | | ***Across TD*** |  | | |  |  | |  | 2807.88 | | | ± | 71.19 | | bc | | 2765.75 | | ± | | 84.00 | c | | 2782.79 | | ± | 83.08 | | | bc | 2998.46 | | ± | 84.10 | | ab | | | 3052.72 | ± | | 56.23 | | a | 3102.80 | | | ± | 54.77 | | a |  | |  |  |  |  |  |  |  |  |
|  |  |  | ***0 N*** | 2995.37 | | | ± | 49.50 | | a | 2899.19 | | | ± | 109.50 | | abcde | | 2930.13 | | ± | | 178.72 | abcd | | 2923.46 | | ± | 87.96 | | | abcd | 3086.28 | | ± | 193.89 | | abc | | | 3051.01 | ± | | 53.91 | | abc | 3082.18 | | | ± | 89.51 | | abc |  | |  |  |  |  |  |  |  |  |
|  |  |  | ***30 N*** | 3041.84 | | | ± | 52.55 | | a | 2940.39 | | | ± | 102.60 | | abcd | | 2812.01 | | ± | | 49.62 | cde | | 2861.79 | | ± | 176.08 | | | bcde | 3113.80 | | ± | 113.36 | | abc | | | 3242.78 | ± | | 61.60 | | ab | 3280.27 | | | ± | 20.46 | | a |  | |  |  |  |  |  |  |  |  |
|  |  |  | ***90 N*** | 2715.91 | | | ± | 46.63 | | b | 2584.05 | | | ± | 88.90 | | de | | 2484.91 | | ± | | 40.79 | e | | 2563.13 | | ± | 111.47 | | | de | 2795.31 | | ± | 81.16 | | cde | | | 2864.37 | ± | | 64.92 | | bcde | 2945.94 | | | ± | 75.64 | | abcd |  | |  |  |  |  |  |  |  |  |
|  |  | |  |  | | |  |  | |  |  | | |  |  | |  | |  | |  | |  |  | |  | |  |  | | |  |  | |  |  | |  | | |  |  | |  | |  |  | | |  |  | |  |  | |  |  |  |  |  |  |  |  |
|  | |  | | |  |  | | |  | | |  |  | | |  | |  | |  | |  | | |  | |  | | |  |  | | |  | | |  | |  |  | | |  | |  | | |  |  | | |  | | |  | |  |  |  |  |  |  |  |
|  |  | ***GGA*** | | | ***Across TD*** | | | |  | | |  |  | | |  | | 0.85 | | ± | | 0.01 | | | a | | 0.84 | | | ± | 0.01 | | | a | | | 0.84 | | ± | 0.02 | | | a | | 0.84 | | | ± | 0.02 | | | a | | | 0.84 | | ± | 0.02 | a | 0.85 | ± | 0.02 | a |
|  |  |  |  |  |  |  | | |  | | |  |  | | |  | |  | |  | |  | | |  | |  | | |  |  | | |  | | |  | |  |  | | |  | |  | | |  |  | | |  | | |  | |  |  |  |  |  |  |  |
|  |  |  |  |  | ***0 N*** | | | | 0.80 | | | ± | 0.01 | | | b | | 0.86 | | ± | | 0.01 | | | abc | | 0.82 | | | ± | 0.02 | | | abcd | | | 0.80 | | ± | 0.04 | | | cd | | 0.77 | | | ± | 0.04 | | | d | | | 0.77 | | ± | 0.03 | d | 0.80 | ± | 0.04 | bcd |
|  |  |  |  |  | ***30 N*** | | | | 0.86 | | | ± | 0.00 | | | a | | 0.86 | | ± | | 0.01 | | | abc | | 0.84 | | | ± | 0.01 | | | abcd | | | 0.87 | | ± | 0.00 | | | ab | | 0.87 | | | ± | 0.01 | | | abc | | | 0.87 | | ± | 0.01 | abc | 0.87 | ± | 0.01 | abc |
|  |  |  |  |  | ***90 N*** | | | | 0.87 | | | ± | 0.01 | | | a | | 0.85 | | ± | | 0.02 | | | abcd | | 0.87 | | | ± | 0.02 | | | ab | | | 0.86 | | ± | 0.01 | | | abc | | 0.89 | | | ± | 0.01 | | | a | | | 0.88 | | ± | 0.01 | a | 0.88 | ± | 0.01 | a |
|  |  |  | | |  |  | | |  | | |  |  | | |  | |  | |  | |  | | |  | |  | | |  |  | | |  | | |  | |  |  | | |  | |  | | |  |  | | |  | | |  | |  |  |  |  |  |  |  |
|  |  | ***CSI*** | | | ***Across TD*** | | | |  | | |  |  | | |  | | 12.98 | | ± | | 0.74 | | | a | | 13.75 | | | ± | 0.97 | | | a | | | 14.18 | | ± | 1.40 | | | a | | 14.80 | | | ± | 1.89 | | | a | | | 13.75 | | ± | 1.29 | a | 13.37 | ± | 1.49 | a |
|  |  |  |  |  |  |  | | |  | | |  |  | | |  | |  | |  | |  | | |  | |  | | |  |  | | |  | | |  | |  |  | | |  | |  | | |  |  | | |  | | |  | |  |  |  |  |  |  |  |
|  |  |  |  |  | ***0 N*** | | | | 17.77 | | | ± | 1.21 | | | a | | 12.44 | | ± | | 1.08 | | | bcde | | 15.94 | | | ± | 1.75 | | | abcde | | | 18.90 | | ± | 3.35 | | | abc | | 21.91 | | | ± | 4.09 | | | a | | | 19.23 | | ± | 2.31 | ab | 18.23 | ± | 3.63 | abcd |
|  |  |  |  |  | ***30 N*** | | | | 12.11 | | | ± | 0.35 | | | b | | 12.49 | | ± | | 0.77 | | | bcde | | 14.11 | | | ± | 1.33 | | | bcde | | | 11.09 | | ± | 0.18 | | | e | | 12.08 | | | ± | 1.00 | | | cde | | | 11.42 | | ± | 0.50 | de | 11.48 | ± | 0.33 | de |
|  |  |  |  |  | ***90 N*** | | | | 11.53 | | | ± | 0.51 | | | b | | 14.02 | | ± | | 1.90 | | | bcde | | 11.20 | | | ± | 1.48 | | | e | | | 12.55 | | ± | 0.79 | | | bcde | | 10.42 | | | ± | 0.73 | | | e | | | 10.60 | | ± | 0.80 | e | 10.40 | ± | 0.96 | e |
|  | |  | | |  |  | | |  | | |  |  | | |  | |  | |  | |  | | |  | |  | | |  |  | | |  | | |  | |  |  | | |  | |  | | |  |  | | |  | | |  | |  |  |  |  |  |  |  |
| CIE | | ***Lightness*** | | | ***Across TD*** | | | |  | | |  |  | | |  | | 43.62 | | ± | | 1.54 | | | a | | 43.50 | | | ± | 1.11 | | | a | | | 43.66 | | ± | 1.41 | | | a | | 45.29 | | | ± | 1.63 | | | a | | | 45.60 | | ± | 1.65 | a | 44.48 | ± | 1.72 | a |
|  |  |  |  |  |  |  | | |  | | |  |  | | |  | |  | |  | |  | | |  | |  | | |  |  | | |  | | |  | |  |  | | |  | |  | | |  |  | | |  | | |  | |  |  |  |  |  |  |  |
|  |  |  |  |  | ***0 N*** | | | | 49.55 | | | ± | 0.88 | | | a | | 48.09 | | ± | | 1.82 | | | abc | | 46.70 | | | ± | 1.51 | | | abcd | | | 49.76 | | ± | 1.88 | | | ab | | 50.68 | | | ± | 2.03 | | | ab | | | 52.50 | | ± | 1.72 | a | 49.55 | ± | 3.48 | ab |
|  |  |  |  |  | ***30 N*** | | | | 43.66 | | | ± | 0.72 | | | b | | 42.37 | | ± | | 1.88 | | | cde | | 44.41 | | | ± | 1.57 | | | bcde | | | 41.11 | | ± | 1.21 | | | de | | 45.61 | | | ± | 2.05 | | | abcde | | | 44.13 | | ± | 1.86 | bcde | 44.30 | ± | 2.00 | bcde |
|  |  |  |  |  | ***90 N*** | | | | 39.87 | | | ± | 0.72 | | | c | | 40.40 | | ± | | 3.15 | | | de | | 39.39 | | | ± | 1.13 | | | e | | | 40.12 | | ± | 1.34 | | | de | | 39.58 | | | ± | 2.06 | | | e | | | 40.16 | | ± | 1.53 | de | 39.60 | ± | 1.65 | e |
|  |  |  | | |  |  | | |  | | |  |  | | |  | |  | |  | |  | | |  | |  | | |  |  | | |  | | |  | |  |  | | |  | |  | | |  |  | | |  | | |  | |  |  |  |  |  |  |  |
|  |  | ***a**** | | | ***Across TD*** | | | |  | | |  |  | | |  | | -22.57 | | ± | | 0.82 | | | a | | -21.94 | | | ± | 0.57 | | | a | | | -22.74 | | ± | 0.84 | | | a | | -23.84 | | | ± | 0.83 | | | a | | | -23.47 | | ± | 0.73 | a | -23.92 | ± | 0.82 | a |
|  |  |  |  |  |  |  | | |  | | |  |  | | |  | |  | |  | |  | | |  | |  | | |  |  | | |  | | |  | |  |  | | |  | |  | | |  |  | | |  | | |  | |  |  |  |  |  |  |  |
|  |  |  |  |  | ***0 N*** | | | | -25.55 | | | ± | 0.39 | | | c | | -24.83 | | ± | | 1.21 | | | defg | | -23.57 | | | ± | 0.50 | | | bcdefg | | | -26.19 | | ± | 0.76 | | | fg | | -26.52 | | | ± | 0.78 | | | g | | | -25.85 | | ± | 0.52 | efg | -26.31 | ± | 1.39 | g |
|  |  |  |  |  | ***30 N*** | | | | -23.02 | | | ± | 0.41 | | | b | | -22.27 | | ± | | 0.99 | | | abcde | | -22.58 | | | ± | 0.50 | | | abcdef | | | -21.57 | | ± | 0.83 | | | abcd | | -23.61 | | | ± | 1.29 | | | bcdefg | | | -23.82 | | ± | 1.19 | bcdefg | -24.27 | ± | 1.06 | cdefg |
|  |  |  |  |  | ***90 N*** | | | | -20.67 | | | ± | 0.43 | | | a | | -20.60 | | ± | | 1.52 | | | ab | | -19.66 | | | ± | 0.91 | | | a | | | -20.46 | | ± | 1.22 | | | ab | | -21.39 | | | ± | 1.32 | | | abcd | | | -20.75 | | ± | 0.80 | abc | -21.16 | ± | 0.82 | abc |
|  |  |  | | |  |  | | |  | | |  |  | | |  | |  | |  | |  | | |  | |  | | |  |  | | |  | | |  | |  |  | | |  | |  | | |  |  | | |  | | |  | |  |  |  |  |  |  |  |
|  |  | ***b**** | | | ***Across TD*** | | | |  | | |  |  | | |  | | 35.53 | | ± | | 1.57 | | | a | | 34.90 | | | ± | 1.19 | | | a | | | 35.56 | | ± | 1.62 | | | a | | 37.59 | | | ± | 1.88 | | | a | | | 37.55 | | ± | 1.82 | a | 37.46 | ± | 2.03 | a |
|  |  |  |  |  |  |  | | |  | | |  |  | | |  | |  | |  | |  | | |  | |  | | |  |  | | |  | | |  | |  |  | | |  | |  | | |  |  | | |  | | |  | |  |  |  |  |  |  |  |
|  |  |  |  |  | ***0 N*** | | | | 42.23 | | | ± | 0.99 | | | a | | 39.75 | | ± | | 2.32 | | | abcd | | 38.48 | | | ± | 1.39 | | | abcde | | | 42.71 | | ± | 1.72 | | | abc | | 44.18 | | | ± | 2.05 | | | ab | | | 44.70 | | ± | 2.08 | a | 43.54 | ± | 3.96 | abc |
|  |  |  |  |  | ***30 N*** | | | | 35.83 | | | ± | 0.80 | | | b | | 34.64 | | ± | | 1.64 | | | def | | 36.13 | | | ± | 1.28 | | | cdef | | | 32.59 | | ± | 1.48 | | | def | | 37.09 | | | ± | 2.59 | | | abcdef | | | 36.76 | | ± | 2.20 | bcdef | 37.74 | ± | 2.22 | abcdef |
|  |  |  |  |  | ***90 N*** | | | | 31.24 | | | ± | 0.80 | | | c | | 32.19 | | ± | | 3.22 | | | def | | 30.09 | | | ± | 1.40 | | | f | | | 31.38 | | ± | 1.75 | | | ef | | 31.51 | | | ± | 2.47 | | | ef | | | 31.18 | | ± | 1.53 | ef | 31.10 | ± | 1.79 | ef |
|  |  |  | | |  |  | | |  | | |  |  | | |  | |  | |  | |  | | |  | |  | | |  |  | | |  | | |  | |  |  | | |  | |  | | |  |  | | |  | | |  | |  |  |  |  |  |  |  |
|  |  | ***u**** | | | ***Across TD*** | | | |  | | |  |  | | |  | | -15.70 | | ± | | 0.71 | | | a | | -15.02 | | | ± | 0.48 | | | a | | | -15.88 | | ± | 0.73 | | | a | | -16.87 | | | ± | 0.67 | | | a | | | -16.36 | | ± | 0.56 | a | -16.98 | ± | 0.64 | a |
|  |  |  |  |  |  |  | | |  | | |  |  | | |  | |  | |  | |  | | |  | |  | | |  |  | | |  | | |  | |  |  | | |  | |  | | |  |  | | |  | | |  | |  |  |  |  |  |  |  |
|  |  |  |  |  | ***0 N*** | | | | -17.99 | | | ± | 0.34 | | | c | | -17.67 | | ± | | 1.08 | | | defg | | -16.29 | | | ± | 0.45 | | | abcdefg | | | -18.66 | | ± | 0.84 | | | efg | | -18.81 | | | ± | 0.70 | | | g | | | -17.74 | | ± | 0.32 | defg | -18.75 | ± | 1.03 | fg |
|  |  |  |  |  | ***30 N*** | | | | -16.18 | | | ± | 0.37 | | | b | | -15.48 | | ± | | 0.92 | | | abcde | | -15.51 | | | ± | 0.41 | | | abcdef | | | -15.04 | | ± | 0.74 | | | abcd | | -16.72 | | | ± | 1.07 | | | bcdefg | | | -16.98 | | ± | 1.08 | bcdefg | -17.34 | ± | 0.90 | cdefg |
|  |  |  |  |  | ***90 N*** | | | | -14.23 | | | ± | 0.39 | | | a | | -13.94 | | ± | | 1.26 | | | ab | | -13.24 | | | ± | 0.87 | | | a | | | -13.95 | | ± | 1.15 | | | ab | | -15.07 | | | ± | 1.15 | | | abcd | | | -14.35 | | ± | 0.69 | abc | -14.86 | ± | 0.67 | abcd |
|  |  |  | | |  |  | | |  | | |  |  | | |  | |  | |  | |  | | |  | |  | | |  |  | | |  | | |  | |  |  | | |  | |  | | |  |  | | |  | | |  | |  |  |  |  |  |  |  |
|  |  | ***v**** | | | ***Across TD*** | | | |  | | |  |  | | |  | | 38.81 | | ± | | 1.85 | | | a | | 38.18 | | | ± | 1.37 | | | a | | | 38.85 | | ± | 1.83 | | | a | | 41.09 | | | ± | 2.10 | | | a | | | 41.16 | | ± | 2.05 | a | 40.66 | ± | 2.24 | a |
|  |  |  |  |  |  |  | | |  | | |  |  | | |  | |  | |  | |  | | |  | |  | | |  |  | | |  | | |  | |  |  | | |  | |  | | |  |  | | |  | | |  | |  |  |  |  |  |  |  |
|  |  |  |  |  | ***0 N*** | | | | 46.35 | | | ± | 1.09 | | | a | | 43.96 | | ± | | 2.54 | | | abcd | | 42.26 | | | ± | 1.65 | | | abcde | | | 46.90 | | ± | 1.98 | | | abc | | 48.31 | | | ± | 2.33 | | | ab | | | 49.36 | | ± | 2.22 | a | 47.31 | ± | 4.40 | abc |
|  |  |  |  |  | ***30 N*** | | | | 39.09 | | | ± | 0.91 | | | b | | 37.62 | | ± | | 2.06 | | | def | | 39.51 | | | ± | 1.60 | | | cdef | | | 35.57 | | ± | 1.65 | | | def | | 40.86 | | | ± | 2.85 | | | abcdef | | | 40.09 | | ± | 2.48 | bcdef | 40.86 | ± | 2.53 | abcdef |
|  |  |  |  |  | ***90 N*** | | | | 33.94 | | | ± | 0.91 | | | c | | 34.84 | | ± | | 3.78 | | | ef | | 32.76 | | | ± | 1.57 | | | f | | | 34.10 | | ± | 1.96 | | | ef | | 34.10 | | | ± | 2.75 | | | ef | | | 34.04 | | ± | 1.82 | ef | 33.81 | ± | 2.03 | ef |
|  | |  | | |  |  | | |  | | |  |  | | |  | |  | |  | |  | | |  | |  | | |  |  | | |  | | |  | |  |  | | |  | |  | | |  |  | | |  | | |  | |  |  |  |  |  |  |  |
| RGB | | ***NGRDI*** | | | ***Across TD*** | | | |  | | |  |  | | |  | | 0.17 | | ± | | 0.00 | | | a | | 0.18 | | | ± | 0.01 | | | a | | | 0.17 | | ± | 0.00 | | | a | | 0.17 | | | ± | 0.00 | | | a | | | 0.17 | | ± | 0.00 | a | 0.18 | ± | 0.00 | a |
|  |  |  |  |  |  |  | | |  | | |  |  | | |  | |  | |  | |  | | |  | |  | | |  |  | | |  | | |  | |  |  | | |  | |  | | |  |  | | |  | | |  | |  |  |  |  |  |  |  |
|  |  |  |  |  | ***0 N*** | | | | 0.17 | | | ± | 0.00 | | | a | | 0.18 | | ± | | 0.01 | | | a | | 0.17 | | | ± | 0.01 | | | a | | | 0.17 | | ± | 0.01 | | | a | | 0.16 | | | ± | 0.01 | | | a | | | 0.18 | | ± | 0.01 | a | 0.18 | ± | 0.00 | a |
|  |  |  |  |  | ***30 N*** | | | | 0.17 | | | ± | 0.00 | | | a | | 0.17 | | ± | | 0.01 | | | a | | 0.17 | | | ± | 0.01 | | | a | | | 0.17 | | ± | 0.01 | | | a | | 0.17 | | | ± | 0.01 | | | a | | | 0.17 | | ± | 0.00 | a | 0.18 | ± | 0.00 | a |
|  |  |  |  |  | ***90 N*** | | | | 0.18 | | | ± | 0.00 | | | a | | 0.17 | | ± | | 0.01 | | | a | | 0.19 | | | ± | 0.01 | | | a | | | 0.18 | | ± | 0.01 | | | a | | 0.17 | | | ± | 0.01 | | | a | | | 0.17 | | ± | 0.01 | a | 0.18 | ± | 0.01 | a |
|  |  |  | | |  |  | | |  | | |  |  | | |  | |  | |  | |  | | |  | |  | | |  |  | | |  | | |  | |  |  | | |  | |  | | |  |  | | |  | | |  | |  |  |  |  |  |  |  |
|  |  | ***TGI*** | | | ***Across TD*** | | | |  | | |  |  | | |  | | 5367.53 | | ± | | 251.93 | | | a | | 5098.91 | | | ± | 301.76 | | | a | | | 4858.06 | | ± | 267.68 | | | a | | 4849.24 | | | ± | 236.37 | | | a | | | 4872.27 | | ± | 276.06 | a | 5190.20 | ± | 274.66 | a |
|  |  |  |  |  |  |  | | |  | | |  |  | | |  | |  | |  | |  | | |  | |  | | |  |  | | |  | | |  | |  |  | | |  | |  | | |  |  | | |  | | |  | |  |  |  |  |  |  |  |
|  |  |  |  |  | ***0 N*** | | | | 4774.37 | | | ± | 189.69 | | | a | | 5097.73 | | ± | | 261.89 | | | a | | 5360.83 | | | ± | 741.91 | | | a | | | 4505.78 | | ± | 509.64 | | | a | | 4230.52 | | | ± | 219.94 | | | a | | | 4455.66 | | ± | 340.74 | a | 4995.72 | ± | 529.38 | a |
|  |  |  |  |  | ***30 N*** | | | | 5281.80 | | | ± | 198.96 | | | a | | 5729.02 | | ± | | 581.19 | | | a | | 4785.34 | | | ± | 504.53 | | | a | | | 5095.21 | | ± | 455.71 | | | a | | 5317.83 | | | ± | 520.17 | | | a | | | 5073.71 | | ± | 519.82 | a | 5689.67 | ± | 456.19 | a |
|  |  |  |  |  | ***90 N*** | | | | 5061.93 | | | ± | 169.22 | | | a | | 5275.82 | | ± | | 455.08 | | | a | | 5150.56 | | | ± | 329.73 | | | a | | | 4973.17 | | ± | 486.36 | | | a | | 4999.38 | | | ± | 333.89 | | | a | | | 5087.43 | | ± | 590.56 | a | 4885.21 | ± | 456.83 | a |
|  | |  | | |  |  | | |  | | |  |  | | |  | |  | |  | |  | | |  | |  | | |  |  | | |  | | |  | |  |  | | |  | |  | | |  |  | | |  | | |  | |  |  |  |  |  |  |  |

Table 4. Effect of the tillage conditions, the top-dressing and the residue applications levels on the RGB indexes derived from the canopy images taken from the ground level with the “phenopole” and from the aerial level with the UAV. Different letters (a, b, c, d, e, f, g, h) indicate significant differences between cultivars within each growing conditions according to Fisher’s LSD test. Significance levels of the ANOVAs: ns, P > 0.05; *, P < 0.05; **, P < 0.01; ***, P < 0.001.

|  |  |  |  |  |  |  | **RGB ground** | | | | | | | | | | | | | | | | | | | | | | | |
| --- | --- | --- | --- | --- | --- | --- | --- | --- | --- | --- | --- | --- | --- | --- | --- | --- | --- | --- | --- | --- | --- | --- | --- | --- | --- | --- | --- | --- | --- | --- |
| ***Index*** | | **Tillage** | ***Across TD*** | | | | ***Conventional Tillage*** | | | | ***No-tillage*** | | | | | | | | | | | | | | | | | | | |
|  |  | ***Residue level*** |  |  |  |  | ***4 Mg/ha*** | | | | ***0 Mg/ha*** | | | | ***2 Mg/ha*** | | | | ***4 Mg/ha*** | | | | ***6 Mg/ha*** | | | | ***8 Mg/ha*** | | | |
|  |  |  |  |  |  |  |  |  |  |  |  |  |  |  |  |  |  |  |  |  |  |  |  |  |  |  |  |  |  |  |
| HIS | ***Hue*** | ***Across TD*** |  |  |  |  | 77.66 | ± | 3.22 | a | 70.51 | ± | 2.73 | a | 75.65 | ± | 3.22 | a | 73.93 | ± | 3.73 | a | 70.98 | ± | 4.01 | a | 67.59 | ± | 3.01 | a |
|  |  | ***0 N*** | 60.36 | ± | 1.63 | c | 66.33 | ± | 4.75 | a | 60.93 | ± | 3.84 | ab | 64.01 | ± | 4.07 | abcd | 59.59 | ± | 4.27 | bcde | 56.85 | ± | 3.43 | defg | 54.44 | ± | 2.68 | efg |
|  |  | ***30 N*** | 74.04 | ± | 1.87 | b | 78.89 | ± | 4.46 | a | 68.70 | ± | 2.09 | abc | 79.08 | ± | 4.57 | abcd | 77.13 | ± | 5.81 | cdef | 71.27 | ± | 6.07 | efg | 69.19 | ± | 2.50 | fg |
|  |  | ***90 N*** | 83.76 | ± | 1.32 | a | 87.76 | ± | 2.90 | a | 81.90 | ± | 1.72 | abcd | 83.87 | ± | 4.18 | abcd | 85.07 | ± | 2.98 | def | 84.83 | ± | 4.77 | efg | 79.15 | ± | 2.07 | g |
|  |  |  |  |  |  |  |  |  |  |  |  |  |  |  |  |  |  |  |  |  |  |  |  |  |  |  |  |  |  |  |
|  | ***Intensity*** | ***Across TD*** |  |  |  |  | 0.31 | ± | 0.00 | a | 0.31 | ± | 0.00 | a | 0.31 | ± | 0.00 | a | 0.31 | ± | 0.00 | a | 0.32 | ± | 0.00 | a | 0.32 | ± | 0.00 | a |
|  |  | ***0 N*** | 0.32 | ± | 0.00 | a | 0.30 | ± | 0.01 | a | 0.32 | ± | 0.01 | a | 0.31 | ± | 0.01 | a | 0.32 | ± | 0.00 | a | 0.31 | ± | 0.01 | a | 0.33 | ± | 0.01 | a |
|  |  | ***30 N*** | 0.32 | ± | 0.00 | a | 0.32 | ± | 0.00 | a | 0.32 | ± | 0.01 | a | 0.32 | ± | 0.00 | a | 0.31 | ± | 0.00 | a | 0.32 | ± | 0.01 | a | 0.32 | ± | 0.01 | a |
|  |  | ***90 N*** | 0.31 | ± | 0.00 | a | 0.31 | ± | 0.00 | a | 0.31 | ± | 0.00 | a | 0.31 | ± | 0.01 | a | 0.31 | ± | 0.01 | a | 0.33 | ± | 0.01 | a | 0.31 | ± | 0.01 | a |
|  |  |  |  |  |  |  |  |  |  |  |  |  |  |  |  |  |  |  |  |  |  |  |  |  |  |  |  |  |  |  |
|  | ***Saturation*** | ***Across TD*** |  |  |  |  | 0.32 | ± | 0.02 | a | 0.32 | ± | 0.02 | a | 0.32 | ± | 0.02 | a | 0.34 | ± | 0.02 | a | 0.35 | ± | 0.02 | a | 0.37 | ± | 0.01 | a |
|  |  | ***0 N*** | 0.39 | ± | 0.01 | a | 0.37 | ± | 0.02 | abc | 0.37 | ± | 0.02 | abc | 0.39 | ± | 0.01 | ab | 0.40 | ± | 0.01 | ab | 0.39 | ± | 0.01 | ab | 0.40 | ± | 0.02 | a |
|  |  | ***30 N*** | 0.34 | ± | 0.01 | b | 0.33 | ± | 0.02 | bcdefg | 0.34 | ± | 0.01 | abcde | 0.31 | ± | 0.03 | cdefg | 0.33 | ± | 0.03 | abcdef | 0.36 | ± | 0.04 | abcd | 0.38 | ± | 0.02 | ab |
|  |  | ***90 N*** | 0.28 | ± | 0.01 | c | 0.26 | ± | 0.02 | g | 0.26 | ± | 0.01 | g | 0.26 | ± | 0.03 | fg | 0.30 | ± | 0.02 | defg | 0.28 | ± | 0.02 | efg | 0.34 | ± | 0.01 | abcdef |
|  |  |  |  |  |  |  |  |  |  |  |  |  |  |  |  |  |  |  |  |  |  |  |  |  |  |  |  |  |  |  |
|  | ***GA*** | ***Across TD*** |  |  |  |  | 0.59 | ± | 0.03 | a | 0.55 | ± | 0.03 | a | 0.56 | ± | 0.03 | a | 0.55 | ± | 0.04 | a | 0.55 | ± | 0.04 | a | 0.52 | ± | 0.04 | a |
|  |  | ***0 N*** | 0.42 | ± | 0.02 | c | 0.47 | ± | 0.05 | ghi | 0.46 | ± | 0.03 | hi | 0.45 | ± | 0.03 | hi | 0.40 | ± | 0.04 | i | 0.37 | ± | 0.04 | i | 0.37 | ± | 0.04 | i |
|  |  | ***30 N*** | 0.57 | ± | 0.01 | b | 0.60 | ± | 0.03 | abcdef | 0.53 | ± | 0.02 | efgh | 0.59 | ± | 0.03 | bcdef | 0.58 | ± | 0.05 | cdefg | 0.56 | ± | 0.05 | defgh | 0.52 | ± | 0.04 | fgh |
|  |  | ***90 N*** | 0.68 | ± | 0.01 | a | 0.70 | ± | 0.03 | ab | 0.66 | ± | 0.01 | abcd | 0.65 | ± | 0.04 | abcde | 0.68 | ± | 0.04 | abc | 0.71 | ± | 0.03 | a | 0.66 | ± | 0.01 | abcd |
|  |  |  |  |  |  |  |  |  |  |  |  |  |  |  |  |  |  |  |  |  |  |  |  |  |  |  |  |  |  |  |
|  | ***GGA*** | ***Across TD*** |  |  |  |  | 0.48 | ± | 0.04 | a | 0.41 | ± | 0.04 | a | 0.44 | ± | 0.04 | a | 0.43 | ± | 0.05 | a | 0.40 | ± | 0.05 | a | 0.36 | ± | 0.05 | a |
|  |  | ***0 N*** | 0.25 | ± | 0.02 | c | 0.33 | ± | 0.06 | def | 0.29 | ± | 0.06 | efg | 0.30 | ± | 0.04 | efg | 0.23 | ± | 0.05 | fg | 0.20 | ± | 0.06 | fg | 0.17 | ± | 0.04 | g |
|  |  | ***30 N*** | 0.44 | ± | 0.02 | b | 0.50 | ± | 0.04 | abc | 0.39 | ± | 0.03 | bcde | 0.49 | ± | 0.04 | abc | 0.47 | ± | 0.06 | abcd | 0.40 | ± | 0.08 | bcde | 0.38 | ± | 0.05 | cde |
|  |  | ***90 N*** | 0.57 | ± | 0.01 | a | 0.60 | ± | 0.03 | a | 0.55 | ± | 0.01 | ab | 0.54 | ± | 0.04 | ab | 0.59 | ± | 0.04 | a | 0.60 | ± | 0.04 | a | 0.53 | ± | 0.02 | abc |
|  |  |  |  |  |  |  |  |  |  |  |  |  |  |  |  |  |  |  |  |  |  |  |  |  |  |  |  |  |  |  |
|  | ***CSI*** | ***Across TD*** |  |  |  |  | 21.53 | ± | 3.62 | a | 27.68 | ± | 4.30 | a | 23.18 | ± | 3.13 | a | 26.36 | ± | 4.55 | a | 32.39 | ± | 5.58 | a | 35.41 | ± | 5.58 | a |
|  |  | ***0 N*** | 43.68 | ± | 3.65 | a | 33.42 | ± | 8.75 | bcdef | 38.67 | ± | 10.21 | abcd | 34.95 | ± | 5.99 | bcde | 45.56 | ± | 7.50 | abc | 50.80 | ± | 9.81 | ab | 58.65 | ± | 9.37 | a |
|  |  | ***30 N*** | 23.42 | ± | 2.07 | b | 17.11 | ± | 2.42 | ef | 26.76 | ± | 5.75 | cdef | 18.11 | ± | 3.39 | def | 19.90 | ± | 3.84 | def | 30.88 | ± | 7.93 | bcdef | 27.76 | ± | 4.01 | cdef |
|  |  | ***90 N*** | 16.18 | ± | 0.94 | c | 14.07 | ± | 1.09 | ef | 17.60 | ± | 0.88 | def | 16.47 | ± | 1.73 | ef | 13.61 | ± | 1.85 | f | 15.49 | ± | 3.31 | ef | 19.81 | ± | 3.53 | def |
|  |  |  |  |  |  |  |  |  |  |  |  |  |  |  |  |  |  |  |  |  |  |  |  |  |  |  |  |  |  |  |
| CIE | ***Lightness*** | ***Across TD*** |  |  |  |  | 39.71 | ± | 0.38 | a | 39.57 | ± | 0.45 | a | 39.57 | ± | 0.36 | a | 40.11 | ± | 0.38 | a | 40.64 | ± | 0.45 | a | 40.61 | ± | 0.45 | a |
|  |  | ***0 N*** | 39.87 | ± | 0.31 | a | 38.79 | ± | 0.68 | a | 39.85 | ± | 1.10 | a | 39.56 | ± | 0.53 | a | 40.10 | ± | 0.17 | a | 39.50 | ± | 0.69 | a | 41.41 | ± | 0.91 | a |
|  |  | ***30 N*** | 40.45 | ± | 0.27 | a | 40.90 | ± | 0.58 | a | 40.21 | ± | 0.59 | a | 40.12 | ± | 0.67 | a | 39.77 | ± | 0.60 | a | 41.07 | ± | 0.80 | a | 40.62 | ± | 0.88 | a |
|  |  | ***90 N*** | 39.79 | ± | 0.30 | a | 39.44 | ± | 0.31 | a | 38.64 | ± | 0.46 | a | 39.04 | ± | 0.67 | a | 40.46 | ± | 1.04 | a | 41.34 | ± | 0.71 | a | 39.81 | ± | 0.49 | a |
|  |  |  |  |  |  |  |  |  |  |  |  |  |  |  |  |  |  |  |  |  |  |  |  |  |  |  |  |  |  |  |
|  | ***a**** | ***Across TD*** |  |  |  |  | -13.49 | ± | 0.94 | a | -10.93 | ± | 0.74 | a | -12.59 | ± | 0.80 | a | -12.66 | ± | 1.12 | a | -11.57 | ± | 1.20 | a | -10.96 | ± | 1.14 | a |
|  |  | ***0 N*** | -8.17 | ± | 0.62 | a | -10.09 | ± | 1.77 | bcdef | -8.29 | ± | 1.42 | abcd | -9.67 | ± | 1.43 | abcde | -8.15 | ± | 1.64 | abc | -6.87 | ± | 1.37 | ab | -5.95 | ± | 1.40 | a |
|  |  | ***30 N*** | -12.89 | ± | 0.46 | b | -14.72 | ± | 1.22 | ghij | -11.09 | ± | 0.55 | cdefg | -13.98 | ± | 1.06 | ghij | -13.54 | ± | 1.08 | fghij | -12.08 | ± | 1.42 | efghi | -11.93 | ± | 0.72 | defgh |
|  |  | ***90 N*** | -15.03 | ± | 0.33 | c | -15.66 | ± | 0.49 | hij | -13.41 | ± | 0.30 | efghij | -14.11 | ± | 0.65 | ghij | -16.28 | ± | 0.83 | j | -15.76 | ± | 1.12 | ij | -14.98 | ± | 0.73 | hij |
|  |  |  |  |  |  |  |  |  |  |  |  |  |  |  |  |  |  |  |  |  |  |  |  |  |  |  |  |  |  |  |
|  | ***b**** | ***Across TD*** |  |  |  |  | 27.84 | ± | 0.79 | a | 27.74 | ± | 0.88 | a | 27.67 | ± | 0.94 | a | 29.20 | ± | 0.78 | a | 29.47 | ± | 0.90 | a | 30.87 | ± | 0.62 | a |
|  |  | ***0 N*** | 30.84 | ± | 0.40 | a | 29.59 | ± | 1.23 | abc | 30.04 | ± | 1.34 | abc | 30.76 | ± | 0.80 | abc | 31.51 | ± | 0.48 | ab | 30.73 | ± | 0.70 | abc | 32.42 | ± | 1.04 | a |
|  |  | ***30 N*** | 29.38 | ± | 0.60 | b | 29.02 | ± | 1.02 | abcd | 29.03 | ± | 0.88 | abcd | 27.55 | ± | 1.53 | bcdef | 28.65 | ± | 1.80 | abcde | 30.74 | ± | 2.08 | abc | 31.28 | ± | 1.14 | ab |
|  |  | ***90 N*** | 26.18 | ± | 0.47 | c | 24.93 | ± | 0.87 | def | 24.15 | ± | 0.80 | f | 24.70 | ± | 1.33 | ef | 27.44 | ± | 0.88 | bcdef | 26.94 | ± | 1.17 | cdef | 28.91 | ± | 0.32 | abcde |
|  |  |  |  |  |  |  |  |  |  |  |  |  |  |  |  |  |  |  |  |  |  |  |  |  |  |  |  |  |  |  |
|  | ***u**** | ***Across TD*** |  |  |  |  | -5.99 | ± | 1.41 | a | -2.68 | ± | 1.21 | a | -4.87 | ± | 1.28 | a | -4.48 | ± | 1.65 | a | -2.94 | ± | 1.78 | a | -1.69 | ± | 1.71 | a |
|  |  | ***0 N*** | 1.95 | ± | 0.94 | a | -1.06 | ± | 2.62 | bcde | 1.54 | ± | 2.22 | abc | -0.13 | ± | 2.13 | abcd | 2.15 | ± | 2.30 | abc | 3.62 | ± | 2.02 | ab | 5.60 | ± | 2.37 | a |
|  |  | ***30 N*** | -4.77 | ± | 0.73 | b | -7.27 | ± | 1.85 | fg | -2.53 | ± | 0.97 | cdef | -6.71 | ± | 1.72 | efg | -5.84 | ± | 1.85 | defg | -3.25 | ± | 2.42 | cdef | -3.00 | ± | 1.15 | cdef |
|  |  | ***90 N*** | -8.51 | ± | 0.45 | c | -9.65 | ± | 0.78 | g | -7.04 | ± | 0.45 | fg | -7.75 | ± | 1.13 | fg | -9.74 | ± | 1.16 | g | -9.20 | ± | 1.69 | g | -7.66 | ± | 0.98 | fg |
|  |  |  |  |  |  |  |  |  |  |  |  |  |  |  |  |  |  |  |  |  |  |  |  |  |  |  |  |  |  |  |
|  | ***v**** | ***Across TD*** |  |  |  |  | 30.06 | ± | 0.57 | ab | 29.48 | ± | 0.70 | b | 29.70 | ± | 0.69 | b | 31.07 | ± | 0.56 | ab | 31.22 | ± | 0.64 | ab | 32.19 | ± | 0.43 | a |
|  |  | ***0 N*** | 31.39 | ± | 0.32 | a | 30.46 | ± | 0.95 | abc | 30.84 | ± | 1.23 | abc | 31.51 | ± | 0.57 | ab | 31.96 | ± | 0.33 | a | 30.95 | ± | 0.64 | abc | 32.64 | ± | 0.72 | a |
|  |  | ***30 N*** | 31.38 | ± | 0.47 | a | 31.61 | ± | 0.62 | ab | 30.76 | ± | 0.73 | abc | 30.09 | ± | 1.24 | abcd | 30.70 | ± | 1.43 | abc | 32.41 | ± | 1.60 | a | 32.70 | ± | 1.03 | a |
|  |  | ***90 N*** | 29.08 | ± | 0.42 | b | 28.11 | ± | 0.75 | bcd | 26.85 | ± | 0.71 | d | 27.48 | ± | 1.04 | cd | 30.54 | ± | 0.89 | abc | 30.29 | ± | 0.85 | abcd | 31.23 | ± | 0.13 | ab |
|  |  |  |  |  |  |  |  |  |  |  |  |  |  |  |  |  |  |  |  |  |  |  |  |  |  |  |  |  |  |  |
| RGB | ***NGRDI*** | ***Across TD*** |  |  |  |  | 0.02 | ± | 0.02 | a | -0.01 | ± | 0.01 | a | 0.01 | ± | 0.01 | a | 0.01 | ± | 0.02 | a | 0.00 | ± | 0.02 | a | -0.01 | ± | 0.02 | a |
|  |  | ***0 N*** | -0.06 | ± | 0.01 | c | -0.04 | ± | 0.02 | fghi | -0.06 | ± | 0.02 | ghi | -0.05 | ± | 0.02 | fghi | -0.07 | ± | 0.02 | hi | -0.07 | ± | 0.02 | hi | -0.09 | ± | 0.02 | i |
|  |  | ***30 N*** | 0.01 | ± | 0.01 | b | 0.03 | ± | 0.02 | abcde | -0.02 | ± | 0.01 | efgh | 0.03 | ± | 0.02 | abcde | 0.02 | ± | 0.03 | bcde | 0.01 | ± | 0.03 | cdef | -0.01 | ± | 0.02 | defg |
|  |  | ***90 N*** | 0.06 | ± | 0.01 | a | 0.07 | ± | 0.01 | ab | 0.05 | ± | 0.01 | abcd | 0.05 | ± | 0.02 | abcd | 0.07 | ± | 0.02 | ab | 0.08 | ± | 0.02 | a | 0.06 | ± | 0.01 | abc |
|  |  |  |  |  |  |  |  |  |  |  |  |  |  |  |  |  |  |  |  |  |  |  |  |  |  |  |  |  |  |  |
|  | ***TGI*** | ***Across TD*** |  |  |  |  | 3076.92 | ± | 71.01 | a | 2788.56 | ± | 57.63 | b | 2960.75 | ± | 59.65 | a | 3115.45 | ± | 81.70 | a | 3022.78 | ± | 95.04 | ab | 3092.48 | ± | 86.35 | b |
|  |  | ***0 N*** | 2790.62 | ± | 50.87 | b | 2885.45 | ± | 146.16 | cdef | 2724.97 | ± | 134.66 | ef | 2944.00 | ± | 100.97 | bcdef | 2850.33 | ± | 148.96 | def | 2641.46 | ± | 132.27 | f | 2697.49 | ± | 73.11 | ef |
|  |  | ***30 N*** | 3158.17 | ± | 40.01 | a | 3317.29 | ± | 50.35 | a | 2922.25 | ± | 57.54 | bcdef | 3097.13 | ± | 102.58 | abcd | 3162.95 | ± | 88.41 | abcd | 3203.77 | ± | 117.08 | abc | 3245.60 | ± | 90.27 | ab |
|  |  | ***90 N*** | 3079.69 | ± | 54.53 | a | 3028.03 | ± | 69.59 | abcde | 2718.47 | ± | 81.97 | ef | 2841.11 | ± | 91.33 | def | 3333.08 | ± | 100.56 | a | 3223.11 | ± | 95.25 | abc | 3334.35 | ± | 72.89 | a |
|  |  |  |  |  |  |  |  |  |  |  |  |  |  |  |  |  |  |  |  |  |  |  |  |  |  |  |  |  |  |  |
|  |  |  |  |  |  |  | ***RGB aerial*** | | | | | | | | | | | | | | | | | | | | | | | |
| ***Index*** | | **Tillage** | ***Across TD*** | | | | ***Conventional Tillage*** | | | | ***No-tillage*** | | | | | | | | | | | | | | | | | | | |
|  |  | ***Residue level*** |  |  |  |  | ***4 Mg/ha*** | | | | ***0 Mg/ha*** | | | | ***2 Mg/ha*** | | | | ***4 Mg/ha*** | | | | ***6 Mg/ha*** | | | | ***8 Mg/ha*** | | | |
|  |  |  |  |  |  |  |  |  |  |  |  |  |  |  |  |  |  |  |  |  |  |  |  |  |  |  |  |  |  |  |
| HIS | ***Hue*** | ***Across TD*** |  |  |  |  | 64.00 | ± | 1.77 | a | 62.03 | ± | 1.66 | a | 61.81 | ± | 1.66 | a | 64.39 | ± | 2.43 | a | 64.94 | ± | 2.57 | a | 63.42 | ± | 2.04 | a |
|  |  | ***0 N*** | 56.98 | ± | 1.03 | c | 59.17 | ± | 2.47 | fg | 60.13 | ± | 3.89 | fg | 55.83 | ± | 2.51 | g | 55.36 | ± | 2.97 | g | 55.66 | ± | 1.54 | g | 55.72 | ± | 1.58 | g |
|  |  | ***30 N*** | 63.16 | ± | 0.71 | b | 63.45 | ± | 2.55 | def | 61.07 | ± | 2.21 | efg | 62.67 | ± | 1.34 | ef | 65.14 | ± | 2.15 | cdef | 63.63 | ± | 0.98 | def | 62.98 | ± | 1.17 | def |
|  |  | ***90 N*** | 70.51 | ± | 0.85 | a | 69.38 | ± | 2.12 | abcd | 65.84 | ± | 0.52 | bcde | 66.92 | ± | 1.09 | bcde | 72.66 | ± | 1.04 | ab | 75.53 | ± | 1.64 | a | 71.55 | ± | 0.39 | abc |
|  |  |  |  |  |  |  |  |  |  |  |  |  |  |  |  |  |  |  |  |  |  |  |  |  |  |  |  |  |  |  |
|  | ***Intensity*** | ***Across TD*** |  |  |  |  | 0.46 | ± | 0.00 | a | 0.46 | ± | 0.00 | a | 0.46 | ± | 0.01 | a | 0.47 | ± | 0.01 | a | 0.47 | ± | 0.01 | a | 0.48 | ± | 0.01 | a |
|  |  | ***0 N*** | 0.48 | ± | 0.00 | a | 0.46 | ± | 0.01 | efg | 0.47 | ± | 0.00 | cdef | 0.48 | ± | 0.01 | abc | 0.49 | ± | 0.01 | ab | 0.50 | ± | 0.01 | a | 0.50 | ± | 0.00 | ab |
|  |  | ***30 N*** | 0.47 | ± | 0.00 | b | 0.47 | ± | 0.01 | cdef | 0.47 | ± | 0.01 | defg | 0.46 | ± | 0.00 | efg | 0.47 | ± | 0.01 | cdef | 0.48 | ± | 0.00 | bcde | 0.48 | ± | 0.01 | abcd |
|  |  | ***90 N*** | 0.44 | ± | 0.00 | c | 0.44 | ± | 0.00 | h | 0.44 | ± | 0.00 | h | 0.45 | ± | 0.00 | gh | 0.44 | ± | 0.01 | h | 0.44 | ± | 0.00 | h | 0.46 | ± | 0.01 | fgh |
|  |  |  |  |  |  |  |  |  |  |  |  |  |  |  |  |  |  |  |  |  |  |  |  |  |  |  |  |  |  |  |
|  | ***Saturation*** | ***Across TD*** |  |  |  |  | 0.25 | ± | 0.01 | a | 0.25 | ± | 0.01 | a | 0.25 | ± | 0.01 | a | 0.26 | ± | 0.01 | a | 0.26 | ± | 0.01 | a | 0.27 | ± | 0.01 | a |
|  |  | ***0 N*** | 0.29 | ± | 0.00 | a | 0.28 | ± | 0.01 | abcde | 0.27 | ± | 0.00 | bcde | 0.29 | ± | 0.01 | abc | 0.30 | ± | 0.00 | a | 0.29 | ± | 0.01 | ab | 0.30 | ± | 0.01 | ab |
|  |  | ***30 N*** | 0.27 | ± | 0.00 | b | 0.26 | ± | 0.01 | de | 0.26 | ± | 0.00 | de | 0.26 | ± | 0.01 | e | 0.26 | ± | 0.01 | cde | 0.28 | ± | 0.01 | bcde | 0.28 | ± | 0.01 | abcd |
|  |  | ***90 N*** | 0.22 | ± | 0.00 | c | 0.22 | ± | 0.01 | f | 0.22 | ± | 0.00 | f | 0.22 | ± | 0.01 | f | 0.22 | ± | 0.00 | f | 0.21 | ± | 0.01 | f | 0.23 | ± | 0.00 | f |
|  |  |  |  |  |  |  |  |  |  |  |  |  |  |  |  |  |  |  |  |  |  |  |  |  |  |  |  |  |  |  |
|  | ***GA*** | ***Across TD*** |  |  |  |  | 0.63 | ± | 0.03 | a | 0.60 | ± | 0.03 | a | 0.59 | ± | 0.03 | a | 0.62 | ± | 0.04 | a | 0.62 | ± | 0.05 | a | 0.59 | ± | 0.04 | a |
|  |  | ***0 N*** | 0.49 | ± | 0.02 | c | 0.54 | ± | 0.04 | efg | 0.56 | ± | 0.06 | ef | 0.47 | ± | 0.04 | fg | 0.46 | ± | 0.05 | g | 0.45 | ± | 0.03 | g | 0.45 | ± | 0.03 | g |
|  |  | ***30 N*** | 0.60 | ± | 0.01 | b | 0.62 | ± | 0.04 | de | 0.58 | ± | 0.03 | e | 0.60 | ± | 0.03 | de | 0.64 | ± | 0.03 | cde | 0.61 | ± | 0.01 | de | 0.58 | ± | 0.02 | e |
|  |  | ***90 N*** | 0.74 | ± | 0.01 | a | 0.73 | ± | 0.04 | abc | 0.67 | ± | 0.01 | bcd | 0.68 | ± | 0.01 | bcd | 0.76 | ± | 0.02 | ab | 0.81 | ± | 0.02 | a | 0.74 | ± | 0.01 | abc |
|  |  |  |  |  |  |  |  |  |  |  |  |  |  |  |  |  |  |  |  |  |  |  |  |  |  |  |  |  |  |  |
|  | ***GGA*** | ***Across TD*** |  |  |  |  | 0.22 | ± | 0.03 | a | 0.19 | ± | 0.02 | a | 0.19 | ± | 0.03 | a | 0.22 | ± | 0.04 | a | 0.23 | ± | 0.05 | a | 0.19 | ± | 0.04 | a |
|  |  | ***0 N*** | 0.10 | ± | 0.01 | c | 0.15 | ± | 0.04 | ghijk | 0.16 | ± | 0.04 | ghijk | 0.09 | ± | 0.03 | hijk | 0.06 | ± | 0.02 | k | 0.07 | ± | 0.02 | ijk | 0.07 | ± | 0.02 | jk |
|  |  | ***30 N*** | 0.18 | ± | 0.01 | b | 0.20 | ± | 0.04 | efg | 0.17 | ± | 0.03 | ghi | 0.19 | ± | 0.03 | fgh | 0.21 | ± | 0.03 | defg | 0.17 | ± | 0.02 | ghij | 0.16 | ± | 0.02 | ghijk |
|  |  | ***90 N*** | 0.34 | ± | 0.02 | a | 0.31 | ± | 0.04 | bcd | 0.26 | ± | 0.01 | bcde | 0.28 | ± | 0.02 | cdef | 0.38 | ± | 0.02 | ab | 0.44 | ± | 0.05 | a | 0.34 | ± | 0.01 | bc |
|  |  |  |  |  |  |  |  |  |  |  |  |  |  |  |  |  |  |  |  |  |  |  |  |  |  |  |  |  |  |  |
|  | ***CSI*** | ***Across TD*** |  |  |  |  | 67.20 | ± | 3.23 | a | 69.19 | ± | 2.39 | a | 70.14 | ± | 3.53 | a | 67.91 | ± | 4.91 | a | 67.96 | ± | 5.23 | a | 70.94 | ± | 4.08 | a |
|  |  | ***0 N*** | 81.01 | ± | 1.96 | a | 73.98 | ± | 5.37 | bcd | 72.73 | ± | 4.39 | cd | 82.09 | ± | 4.89 | abc | 87.09 | ± | 3.94 | a | 85.07 | ± | 4.01 | ab | 85.08 | ± | 3.28 | ab |
|  |  | ***30 N*** | 70.51 | ± | 1.42 | b | 69.26 | ± | 5.46 | de | 71.36 | ± | 3.39 | cd | 69.33 | ± | 4.36 | de | 67.03 | ± | 3.15 | de | 72.53 | ± | 2.18 | cd | 73.57 | ± | 2.75 | bcd |
|  |  | ***90 N*** | 54.54 | ± | 1.54 | c | 58.36 | ± | 3.69 | ef | 61.59 | ± | 1.68 | ef | 59.01 | ± | 2.17 | ef | 49.59 | ± | 2.17 | fg | 46.28 | ± | 4.29 | g | 54.18 | ± | 1.48 | fg |
|  |  |  |  |  |  |  |  |  |  |  |  |  |  |  |  |  |  |  |  |  |  |  |  |  |  |  |  |  |  |  |
| CIE | ***Lightness*** | ***Across TD*** |  |  |  |  | 54.15 | ± | 0.59 | a | 54.25 | ± | 0.53 | a | 54.91 | ± | 0.66 | a | 55.51 | ± | 0.85 | a | 56.21 | ± | 0.89 | a | 56.83 | ± | 0.66 | a |
|  |  | ***0 N*** | 57.33 | ± | 0.41 | a | 55.15 | ± | 0.89 | ef | 55.38 | ± | 0.20 | def | 57.28 | ± | 0.85 | abcd | 58.37 | ± | 0.60 | ab | 59.08 | ± | 0.93 | a | 58.70 | ± | 0.34 | ab |
|  |  | ***30 N*** | 55.95 | ± | 0.33 | b | 55.36 | ± | 0.71 | def | 55.05 | ± | 0.46 | f | 54.79 | ± | 0.67 | f | 55.82 | ± | 1.05 | cdef | 57.03 | ± | 0.32 | bcde | 57.64 | ± | 0.65 | abc |
|  |  | ***90 N*** | 52.59 | ± | 0.24 | c | 51.95 | ± | 0.21 | h | 51.69 | ± | 0.17 | h | 52.66 | ± | 0.33 | gh | 52.35 | ± | 0.65 | gh | 52.52 | ± | 0.54 | gh | 54.13 | ± | 0.66 | fg |
|  |  |  |  |  |  |  |  |  |  |  |  |  |  |  |  |  |  |  |  |  |  |  |  |  |  |  |  |  |  |  |
|  | ***a**** | ***Across TD*** |  |  |  |  | -10.13 | ± | 0.45 | a | -9.62 | ± | 0.54 | a | -9.51 | ± | 0.49 | a | -10.56 | ± | 0.72 | a | -10.83 | ± | 0.62 | a | -10.62 | ± | 0.57 | a |
|  |  | ***0 N*** | -8.54 | ± | 0.39 | a | -9.05 | ± | 0.83 | abc | -9.43 | ± | 1.48 | abc | -8.02 | ± | 0.98 | a | -8.10 | ± | 1.41 | a | -8.29 | ± | 0.54 | ab | -8.33 | ± | 0.70 | ab |
|  |  | ***30 N*** | -10.60 | ± | 0.24 | b | -10.39 | ± | 0.71 | abcde | -9.56 | ± | 0.66 | abc | -10.10 | ± | 0.62 | abcd | -11.31 | ± | 0.67 | cde | -11.18 | ± | 0.31 | cde | -11.06 | ± | 0.29 | cde |
|  |  | ***90 N*** | -11.58 | ± | 0.28 | c | -10.95 | ± | 0.61 | bcde | -9.95 | ± | 0.20 | abcde | -10.39 | ± | 0.48 | abcde | -12.27 | ± | 0.40 | de | -13.03 | ± | 0.22 | e | -12.49 | ± | 0.27 | de |
|  |  |  |  |  |  |  |  |  |  |  |  |  |  |  |  |  |  |  |  |  |  |  |  |  |  |  |  |  |  |  |
|  | ***b**** | ***Across TD*** |  |  |  |  | 30.12 | ± | 0.93 | a | 30.31 | ± | 0.73 | a | 30.65 | ± | 1.00 | a | 31.66 | ± | 1.07 | a | 31.96 | ± | 1.11 | a | 32.75 | ± | 0.91 | a |
|  |  | ***0 N*** | 34.14 | ± | 0.39 | a | 32.39 | ± | 1.23 | bcd | 32.28 | ± | 0.39 | bcd | 34.04 | ± | 0.82 | ab | 35.62 | ± | 0.39 | a | 35.16 | ± | 0.71 | a | 35.37 | ± | 0.59 | a |
|  |  | ***30 N*** | 32.12 | ± | 0.43 | b | 31.30 | ± | 1.26 | cd | 30.96 | ± | 0.44 | de | 30.78 | ± | 1.32 | de | 32.01 | ± | 0.94 | bcd | 33.57 | ± | 0.60 | abc | 34.11 | ± | 0.57 | ab |
|  |  | ***90 N*** | 27.34 | ± | 0.27 | c | 26.66 | ± | 0.58 | f | 26.82 | ± | 0.19 | f | 27.14 | ± | 0.74 | f | 27.36 | ± | 0.49 | f | 27.16 | ± | 0.81 | f | 28.77 | ± | 0.52 | ef |
|  |  |  |  |  |  |  |  |  |  |  |  |  |  |  |  |  |  |  |  |  |  |  |  |  |  |  |  |  |  |  |
|  | ***u**** | ***Across TD*** |  |  |  |  | -0.15 | ± | 0.91 | a | 0.66 | ± | 0.89 | a | 0.99 | ± | 0.98 | a | -0.10 | ± | 1.37 | a | -0.35 | ± | 1.30 | a | 0.24 | ± | 1.14 | a |
|  |  | ***0 N*** | 3.83 | ± | 0.66 | a | 2.29 | ± | 1.52 | abc | 1.71 | ± | 2.16 | abcd | 4.55 | ± | 1.71 | ab | 5.07 | ± | 2.07 | a | 4.67 | ± | 1.06 | ab | 4.66 | ± | 1.10 | ab |
|  |  | ***30 N*** | -0.03 | ± | 0.37 | b | -0.05 | ± | 1.36 | cde | 1.02 | ± | 1.12 | bcde | 0.13 | ± | 0.72 | cde | -1.10 | ± | 1.06 | cdefg | -0.31 | ± | 0.55 | cdef | 0.10 | ± | 0.66 | cde |
|  |  | ***90 N*** | -3.31 | ± | 0.37 | c | -2.68 | ± | 0.88 | efgh | -1.23 | ± | 0.22 | defgh | -1.71 | ± | 0.51 | defgh | -4.28 | ± | 0.49 | gh | -5.41 | ± | 0.52 | h | -4.02 | ± | 0.23 | fgh |
|  |  |  |  |  |  |  |  |  |  |  |  |  |  |  |  |  |  |  |  |  |  |  |  |  |  |  |  |  |  |  |
|  | ***v**** | ***Across TD*** |  |  |  |  | 35.44 | ± | 0.93 | a | 35.55 | ± | 0.76 | a | 35.97 | ± | 1.01 | a | 37.23 | ± | 1.07 | a | 37.73 | ± | 1.10 | a | 38.55 | ± | 0.89 | a |
|  |  | ***0 N*** | 39.49 | ± | 0.42 | a | 37.50 | ± | 1.22 | cde | 37.55 | ± | 0.46 | cde | 39.29 | ± | 0.82 | abcd | 40.96 | ± | 0.55 | a | 40.79 | ± | 0.73 | a | 40.88 | ± | 0.59 | a |
|  |  | ***30 N*** | 37.78 | ± | 0.47 | b | 36.86 | ± | 1.24 | def | 36.32 | ± | 0.39 | ef | 36.19 | ± | 1.41 | ef | 37.80 | ± | 1.08 | bcde | 39.46 | ± | 0.58 | abc | 40.07 | ± | 0.58 | ab |
|  |  | ***90 N*** | 32.84 | ± | 0.32 | c | 31.95 | ± | 0.59 | h | 31.85 | ± | 0.24 | h | 32.44 | ± | 0.81 | gh | 32.94 | ± | 0.59 | gh | 32.94 | ± | 0.84 | gh | 34.70 | ± | 0.64 | fg |
|  |  |  |  |  |  |  |  |  |  |  |  |  |  |  |  |  |  |  |  |  |  |  |  |  |  |  |  |  |  |  |
| RGB | ***NGRDI*** | ***Across TD*** |  |  |  |  | 0.00 | ± | 0.01 | a | -0.01 | ± | 0.01 | a | -0.01 | ± | 0.01 | a | 0.00 | ± | 0.01 | a | 0.00 | ± | 0.01 | a | 0.00 | ± | 0.01 | a |
|  |  | ***0 N*** | -0.03 | ± | 0.00 | c | -0.02 | ± | 0.01 | fgh | -0.02 | ± | 0.01 | efgh | -0.03 | ± | 0.01 | h | -0.03 | ± | 0.01 | h | -0.03 | ± | 0.01 | gh | -0.03 | ± | 0.01 | gh |
|  |  | ***30 N*** | 0.00 | ± | 0.00 | b | -0.01 | ± | 0.01 | cdefg | -0.01 | ± | 0.01 | defgh | -0.01 | ± | 0.00 | cdefg | 0.00 | ± | 0.01 | bcde | 0.00 | ± | 0.00 | cdef | 0.00 | ± | 0.00 | cdef |
|  |  | ***90 N*** | 0.02 | ± | 0.00 | a | 0.01 | ± | 0.01 | abc | 0.00 | ± | 0.00 | bcd | 0.01 | ± | 0.00 | bcd | 0.03 | ± | 0.00 | ab | 0.04 | ± | 0.01 | a | 0.02 | ± | 0.00 | ab |
|  |  |  |  |  |  |  |  |  |  |  |  |  |  |  |  |  |  |  |  |  |  |  |  |  |  |  |  |  |  |  |
|  | ***TGI*** | ***Across TD*** |  |  |  |  | 2807.88 | ± | 71.19 | bc | 2765.75 | ± | 84.00 | c | 2782.79 | ± | 83.08 | bc | 2998.46 | ± | 84.10 | ab | 3052.72 | ± | 56.23 | a | 3102.80 | ± | 54.77 | a |
|  |  | ***0 N*** | 2995.37 | ± | 49.50 | a | 2899.19 | ± | 109.50 | abcde | 2930.13 | ± | 178.72 | abcd | 2923.46 | ± | 87.96 | abcd | 3086.28 | ± | 193.89 | abc | 3051.01 | ± | 53.91 | abc | 3082.18 | ± | 89.51 | abc |
|  |  | ***30 N*** | 3041.84 | ± | 52.55 | a | 2940.39 | ± | 102.60 | abcd | 2812.01 | ± | 49.62 | cde | 2861.79 | ± | 176.08 | bcde | 3113.80 | ± | 113.36 | abc | 3242.78 | ± | 61.60 | ab | 3280.27 | ± | 20.46 | a |
|  |  | ***90 N*** | 2715.91 | ± | 46.63 | b | 2584.05 | ± | 88.90 | de | 2484.91 | ± | 40.79 | e | 2563.13 | ± | 111.47 | de | 2795.31 | ± | 81.16 | cde | 2864.37 | ± | 64.92 | bcde | 2945.94 | ± | 75.64 | abcd |
|  |  |  |  |  |  |  |  |  |  |  |  |  |  |  |  |  |  |  |  |  |  |  |  |  |  |  |  |  |  |  |
